# Backbone editing and deconstruction of polyethylene by Beckmann rearrangement and hydrogenolysis†

**DOI:** 10.1039/d5sc02684a

**Published:** 2025-06-02

**Authors:** Jake X. Shi, Diane D. Kim, Nicodemo R. Ciccia, Pierre Lahaie-Boivin, John F. Hartwig

**Affiliations:** a Department of Chemistry, University of California Berkeley California 94720 USA jhartwig@berkeley.edu; b Division of Chemical Sciences, Lawrence Berkeley National Laboratory Berkeley California 94720 USA

## Abstract

Polyethylene is the most widely produced commodity plastic and is used in many applications, including packaging, insulation, and medical devices. However, the inertness of polyethylene makes chemical recycling inefficient and challenging. We report the conversion of oxidized high-density and low-density polyethylene, formed by direct, catalytic oxidation, to polyamides by Beckmann rearrangement of the corresponding oximes. These polyamides have enhanced surface properties over those of unmodified polyethylene, while maintaining the same, favorable mechanical profiles. The amide sites were reductively cleaved by hydrogenolysis with ruthenium-based catalysts to furnish alcohol- and amine-terminated fragments, which were used for the synthesis of polyurea-urethanes with poly(tetrahydrofuran) and methylene diphenyl diisocyanate. These experiments show how to install cleavable moieties into the backbone of polyethylene to facilitate deconstruction and the generation of new materials to affect greater sustainability in polyolefins.

## Abstract 2

Conversion of oxime-modified polyethylenes to polyamides by Beckmann rearrangement creates materials with enhanced adhesion. Deconstruction by hydrogenolysis furnishes fragments that serve as precursors for new materials.

## Graphical abstract

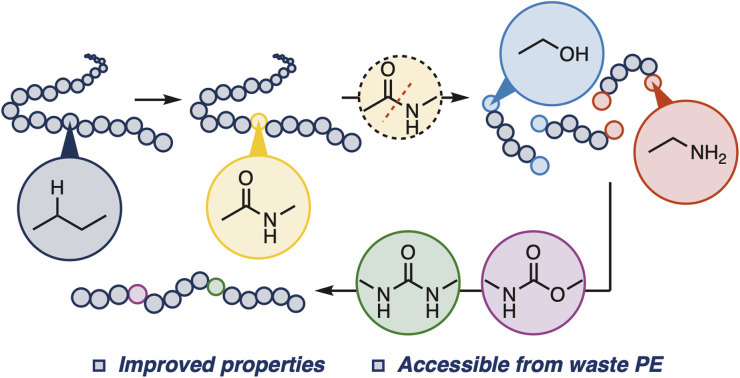

## Introduction

Polyethylene is formed in the largest quantity of any plastic, with global production currently exceeding 110 million metric tons annually.^1^ Its durability and inertness render it useful over a range of applications, from packaging to construction; however, its resistance to chemical transformations necessitates that it be blended or layered with polar polymers to broaden the range of properties that polyethylene can possess.^2,3^ The resulting polymer composites, the formation of which typically requires compatibilizers and other additives, cannot readily be separated into their components after use, and are, therefore, challenging to recycle.^3–6^ For this reason, many polyethylene products are produced for single-use applications and are major contributors to the accumulation of plastic waste.^1,3^

Functionalized polyethylenes could serve as a more sustainable alternative to these polyethylene composites. They can be tailored to possess enhanced properties, such as increased adhesion and solubility in polar solvents over their unmodified counterparts, whereas accessing such properties from polyethylene would require polymer blending. The functionalized polymers have been shown to be more amenable to deconstruction and recycling than composite materials.^7–13^ Typically, these functionalized polyolefins are synthesized by copolymerization of ethylene and polar comonomers. However, copolymerization methods are limited; free-radical copolymerization does not allow for a significant degree of control over monomer ratios or polymer architecture, and copolymerization catalyzed by transition-metal systems is prone to catalyst deactivation by polar functional groups.^8,9^ Therefore, these processes can generate only a limited range of functional materials.

Post-polymerization functionalization can circumvent some of these disadvantages by enabling the structure of the polymer to be established prior to the introduction of polar groups.^8,9^ In addition, post-polymerization functionalization can occur with existing polyolefins as feedstock to upcycle post-consumer polyolefins directly into functional materials suitable for a range of applications.^14–17^

Our group reported a ruthenium-catalyzed oxidation of the C–H bonds in polyethylenes of varying architectures and in waste polyethylenes to generate oxo-polyethylenes containing a mixture of pendant alcohol and ketone units.^18^ These oxo-polyethylenes displayed enhanced bulk properties and were amenable to further modification by conversion of all installed polar moieties to either the alcohols or the ketones, followed by grafting of substituents containing functional groups. One such transformation generated oxime-containing polyethylenes by reaction of ketone-functionalized polyethylene with a variety of *O*-substituted hydroxylammonium chloride salts. The resulting oxime-polyethylenes displayed enhanced adhesion over the unmodified polyethylene and could be reverted to their ketone-containing precursors by simple hydrolysis. Because most previously reported functionalizations of polyolefins modify only the pendent C–H bonds, methods for in-chain modification that could further broaden the range of polymer structures and properties accessible by post-polymerization functionalization are needed.^19–21^

**Fig. 1:**
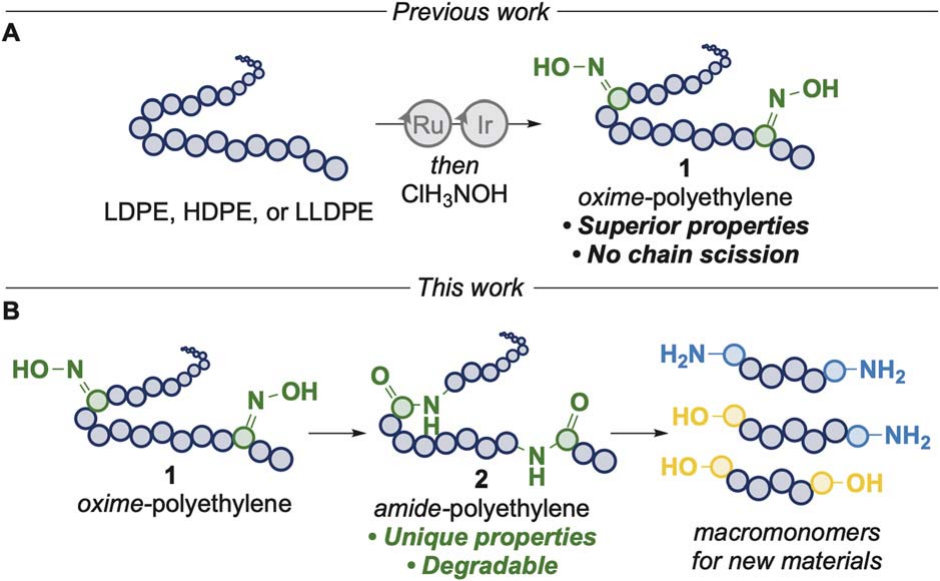
(A) Prior work: synthesis of oxime-polyethylene. (B) This work: Beckmann rearrangement of oxime-polyethylene to form in-chain amide linkages and hydrogenolysis to create precursors for new materials.

We envisioned that further derivatization of oxime-polyethylenes could generate polymers with in-chain amide linkages by a Beckmann rearrangement and that the resulting polymers could have properties different from those of polyethylene or that they could be cleaved to form telechelic units to create new materials with greater circularity, or both (Fig. 1). During the preparation of this manuscript, Nozaki and coworkers published the formation of polyamides from linear polyketones generated from palladium-catalyzed copolymerization of ethylene and diiron nonacarbonyl, by sequential oxime formation and Beckmann rearrangement using a large excess of diethylamino sulfur trifluoride (DAST) and assessed the thermal and mechanical profiles of the polyamides.^22^ However, this work was not extended to commercial polyethylenes or those derived from waste. De Vos and coworkers reported the synthesis of polyamides from the direct oxidation of low molecular weight polyethylenes (*M*_n_ < 2000 Da), by sequential oxime formation and Beckmann rearrangement with sulfuric acid at high temperatures, but the latter step reverted some of the oximes back to ketones.^23^ Aube, Zhukhovitskiy and coworkers demonstrated the rearrangement of alcohol and ketone units on oxidized low-molecular weight polyethylene (*M*_n_ < 2000 Da) with alkyl azides and scandium(iii) triflate to furnish polyamides with tertiary amide linkages, but only moderate yields of amide were achieved and significant amounts of chain scission was observed.^24^

To address these limitations, we envisioned that access to these polyamides from the direct oxidation of high molecular weight polyethylenes, using mild, effective catalysts and reagents with minimal side reactions is needed. Here, we report the synthesis of amide-containing polyethylenes from multiple forms of oxo-polyethylene from commercial and waste polyethylenes, testing of thermal, tensile and surface properties of the materials, and the cleavage of the amide linkages to form telechelic macromonomers that serve as chain extenders for the synthesis of polyurea urethanes.

## Results and discussion

### Beckmann rearrangement of oxime-polyethylene

The conversion of polyketones to polyamides can be achieved in one step by a Schmidt reaction.^25,26^ Our group has previously reported a method to access polyketones directly from polyethylene by sequential C–H functionalization and oxidation.^27^ However, because keto-polyethylenes derived from our strategy require higher temperatures to dissolve than polyketones synthesized by radical polymerization, the high operating temperatures required to dissolve keto-polyethylene discourages the use of hydrazoic acid for this transformation.^28^

Thus, we started our work from oxime-containing low-density polyethylene (LDPE) 1a derived from oxidation and condensation of LDPE to furnish in-chain amide linkages through Beckmann rearrangement. In 1961, the Beckmann rearrangement of keto-polyethylene into polyamides through sequential oxime installation and reaction with phosphorus pentachloride (PCl_5_) was reported; however, when applied to our system from commercial polyethylenes, generation of alkyl chlorides by Beckmann fragmentation occurred, as assessed by ^1^H NMR spectroscopy (Fig. S41†).^26^

Thus, we investigated for the reactions of our oxidized polyolefins alternative conditions that readily convert small-molecule oximes to amides (Table 1).^29^ Organocatalysts, such as cyanuric chloride (CNC) and the combination of triphenylphosphine with carbon tetrabromide,^30,31^ did not catalyze the formation of amides from the oximes (Table 1 entries 1 and 2). Furthermore, strong acids, such as *p*-toluenesulfonic acid (PTSA) and trifluoroacetic acid (TFA), formed the amides in just trace amounts (Table 1 entries 3 and 4). Low conversions of the oxime occurred from reactions with trifluoroacetic anhydride (TFAA) (Table 1 entry 5). The low conversions of the reactions with these reagents highlight the challenges of applying transformations of small molecules to polyethylene.

**Table 1:** Optimization of the Beckmann rearrangement of oxime-LDPE 1a to amide-LDPE 2a

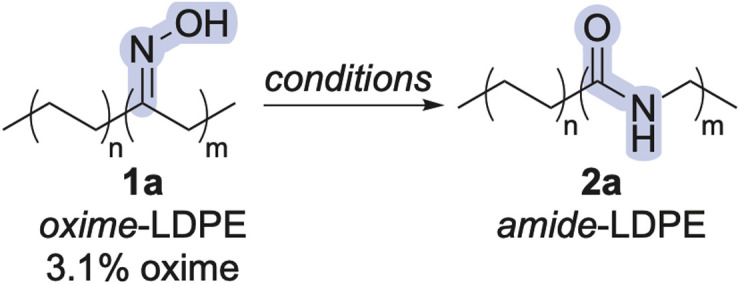				
Entry^a^	Conditions^g^	Conversion^h^	*M*_n_ (kDa)^i^^,^^j^	*Đ*
1^b^^,^^e^	1 equiv. PPh_3_, 1 equiv. CBr_4_	n.r.	—	—
2^b^^,^^e^	1 equiv. CNC	n.r.	—	—
3^b^^,^^e^	5 equiv. PTSA	Trace	—	—
4^c^^,^^f^	50 equiv. TFA	Trace	—	—
5^c^^,^^f^	30 equiv. TFAA	15%	—	—
6^b^^,^^e^	5 equiv. TsCl	>99%	2.7	2.3
7^d^^,^^f^	3 equiv. T3P	>99%	8.1	2.4

a All reactions run for 24 h.

b Reaction run in PhMe.

c Reaction run in DCM.

d Reaction run in THF.

e Reaction run at 120 °C.

f Reaction run at 80 °C.

g Equivalents with respect to the number of oximes.

h Conversion of oximes to amides as determined by ^1^H NMR spectroscopy in CDCl_3_ at room temperature.

i Molecular weight with respect to polyethylene standards.

j *M*_n,LDPE_ = 9.6 kDa and *Đ*_LDPE_ = 6.7. n.r. = no reaction. CNC = cyanuric chloride. PTSA = *p*-toluenesulfonic acid. TFA = trifluoroacetic acid. TFAA = trifluoroacetic anhydride. T3P = propylphosphonic anhydride.

Instead, reactions conducted with *para*-toluenesulfonyl chloride (TsCl) and propylphosphonic anhydride (T3P) converted the oximes quantitatively, as assessed by ^1^H NMR spectroscopy (Table 1 entries 6 and 7); however, hydrolysis of the oxime to the ketone was also observed after reactions with TsCl (presumably catalyzed by the HCl that is formed as a byproduct). In addition, a substantial decrease in *M*_n_ from unmodified LDPE to amide-LDPE 2a was observed after reactions with TsCl, presumably caused by competing Beckmann fragmentation (Table 1 entry 6).^32^ A lower extent of hydrolysis of the oxime to the ketone was observed after reactions with T3P, and less chain scission was observed from reactions with T3P than from reactions with TsCl (Table 1, entry 7). Notably, the dispersity of amide-LDPE 2a (*Đ* = 2.0) decreased from the starting LDPE (*Đ* = 6.7). We hypothesized that the decrease in dispersity of the polymer could be caused by the transamidation of the polymer chains over the course of the reaction. To probe this hypothesis, we synthesized small-molecule oximes and subjected them to the Beckmann rearrangement with T3P (see Scheme S1†). However, no scrambling of the alkyl chains was observed by GC-MS, indicating that no transamidation occurs over the course of the reaction and that the decrease in dispersity of the polymers after rearrangement is not due to transamidation. Instead, we propose that this decrease could be due to a change in polymer conformation that affects its behavior in size-exclusion chromatography.

**Scheme 1:**
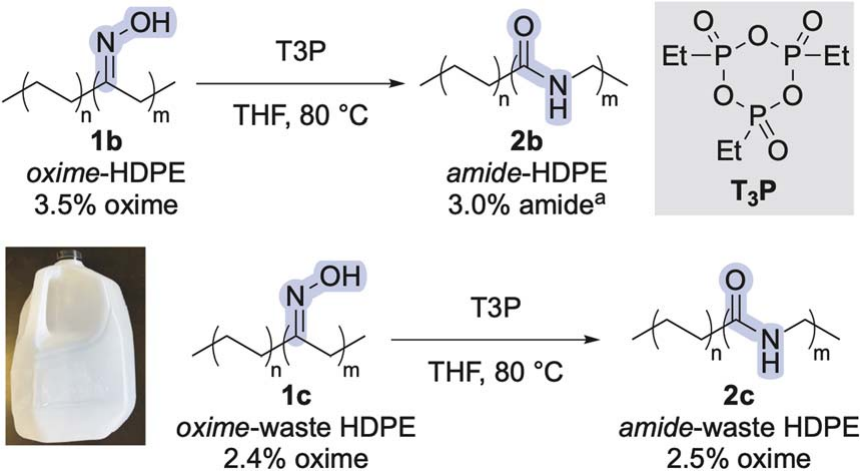
Beckmann rearrangement of additional oxime-polyethylenes with T3P. ^*a*^The small difference in percent functionality could be attributed to the inherent error of determining percent functionalization of this level of groups in a polymer by ^1^H NMR spectroscopy.

Based on these results, we reasoned that T3P could be a safer, cheaper, and more selective reagent than DAST, H_2_SO_4_, or Sc(OTf)_3_ for the synthesis of amide-polyethylenes from other polyethylenes. To this end, we applied the reaction conditions to oxime-polyethylenes derived from high-density polyethylene (HDPE) and post-consumer HDPE from a milk jug (Scheme 1). The conversion of oximes to amides in all these polymers was quantitative, as judged by ^1^H NMR spectroscopy (see ESI†), indicating installation of amide linkages into the main chains of polyethylene.

### Hydrogenolysis of amide linkages

To cleave amide-LDPE 2a into end-functionalized fragments, we sought to develop methods for hydrogenolysis of this material at the amide linkages. Because amide-LDPE 2a (*M*_n_ = 8.1 kDa) contains approximately 2% of amide linkages (average of six amides per chain), cleavage at these linkages would generate an average of five telechelic segments and two monofunctional segments.

**Fig. 2:**
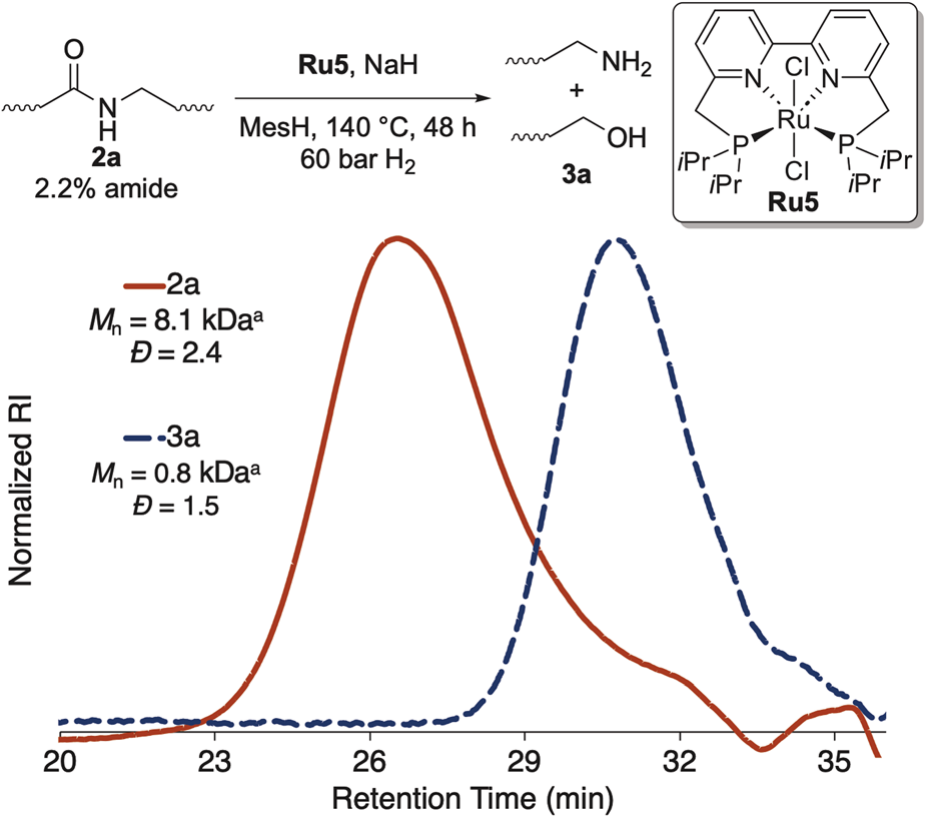
Hydrogenolysis of amide linkages in amide-LDPE. ^*a*^Molecular weight with respect to polyethylene standards.

We tested the ability of several ruthenium complexes that have been reported to catalyze the hydrogenolysis of aliphatic amides to catalyze hydrogenolysis of the amide units in polymer 2a (see Table S1†).^33–37^ We found that air-stable catalyst Ru5, which contains a tetradentate bipyridyl bisphosphine ligand, converted the amide linkages in polymer 2a quantitatively to alcohol- and amine-terminated oligomers, as assessed by ^1^H NMR spectroscopy. A decrease in *M*_n_ was also observed by high-temperature, size-exclusion chromatography (HTSEC), indicating that polymer 2a was cleaved at the amide linkages (Fig. 2). We hypothesized that the activity of catalyst Ru5 for hydrogenolysis of polymer 2a can be attributed to the resistance of the tetradentate bipyridyl bisphosphine ligand to dissociate from the ruthenium because of its high binding affinity. Application of the optimized conditions to amide-polyethylenes 2b and 2c afforded complete hydrogenolysis of the amide linkages, as assessed by ^1^H NMR spectroscopy (see ESI†). These results show that this system for catalytic hydrogenolysis operates on polyamides with various architectures and tolerates additives that may remain in amide-polyethene 2c derived from post-consumer waste HDPE.

### Polymerization of alcohol- and amine-terminated fragments

**Scheme 2:**
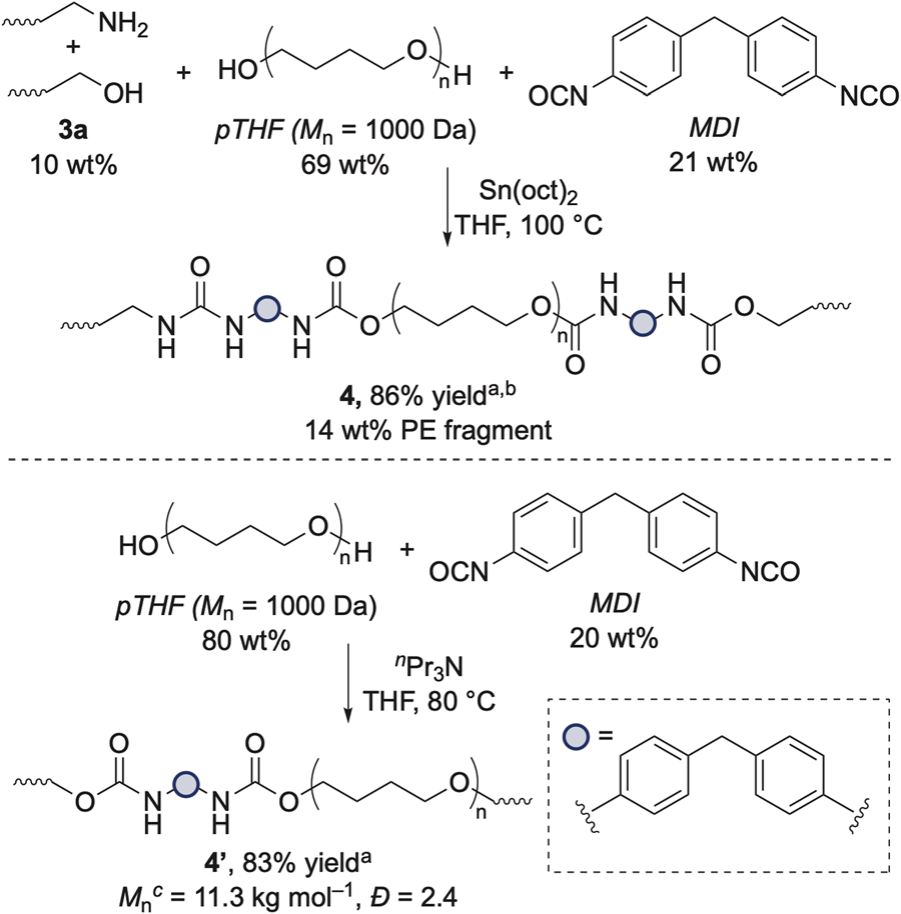
Synthesis of PUUs through polymerization of alcohol- and amine-terminated LDPE fragments with MDI and *p*THF. ^*a*^Yield with respect to mass. ^*b*^We were unable to characterize the resulting material by gel-permeation chromatography (GPC) because of its insolubility in THF at 35 °C, DMF at 55 °C, or 1,2,4-trichlorobenzene at 135 °C. ^*c*^Absolute molecular weight was calculated following detector calibration with a single narrow poly(styrene) standard.

The oligomers resulting from the hydrogenolysis of polymer 2a at the amide linkages could serve as precursors to new materials. For example, these long-chain alcohol- and amine-terminated segments could be used as chain extenders for the synthesis of polyurea-urethane (PUU) elastomers derived from waste polyethylene. PUUs are valuable materials that are durable, self-healable, and reprocessable.^38,39^ To this end, the alcohol- and amine-terminated segments 3a were polymerized with methylene diphenyl diisocyanate (MDI) and poly(tetrahydrofuran) (*p*THF, *M*_n_ = 1000 Da) as the soft segment catalyzed by tin(ii) 2-ethylhexanoate (Sn(oct)_2_) to form PUU 4 (Scheme 2). ^1^H NMR spectroscopy at 100 °C showed that polymerization of the soft and hard segments occurred with MDI (see Fig. S35†). We also synthesized pTHF/MDI polyurethane (PU) 4′ in the absence of polyethylene fragments to serve as an additional comparison for the properties of these materials.

### Materials testing

**Fig. 3:**
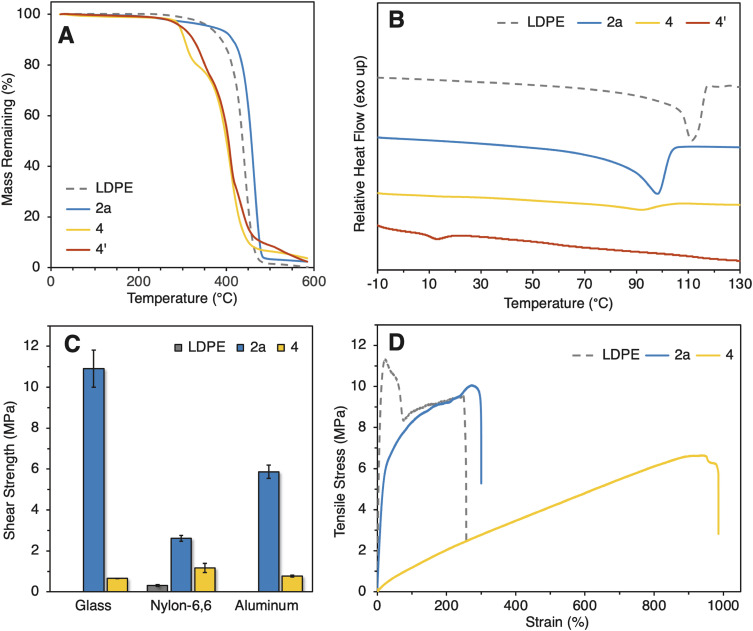
Summary of materials properties. (A) TGA curves of unmodified LDPE, polymer 2a, PUU 4, and PU 4′. (B) DSC traces of unmodified LDPE, polymer 2a, PUU 4, and PU 4′. (C) Lap-shear tests of unmodified LDPE, polymer 2a, and PUU 4 as interlayers with aluminum, nylon-6,6, and glass substrates. The mode of failure for all lap-shear tests was determined to be adhesive failure. (D) Stress–strain curves of unmodified LDPE, polymer 2a, and PUU 4. Strain rate = 50 mm min^−1^.

With these polymers in hand, we investigated their properties. To gauge the thermal properties of polyamide 2a and PUU 4, we performed thermal gravimetric analysis (TGA) and differential scanning calorimetry (DSC). TGA revealed that the decomposition temperature (*T*_d_) of amide-LDPE 2a (368.5 °C) was comparable to the *T*_d_ of unmodified LDPE (360.8 °C), indicating that the thermal stability of the polymer does not change appreciably by the transformations to form the polyamide (Fig. 3A). TGA revealed a two-stage decomposition of PUU 4, with the first stage occurring at 290 °C and the second stage occurring at 371 °C. We speculate that the first stage corresponds to the degradation of the polyolefin segments because the mass lost is approximately equal to the weight percent of polyolefin segments incorporated in PUU 4 (see ESI†) and because the decomposition profile of PU 4′, which does not contain polyolefin segments, parallels the second decomposition stage of PUU 4. DSC showed that the melting temperature (*T*_m_) of unmodified LDPE is 111 °C, whereas the *T*_m_ of amide-LDPE 2a was a lower 98.1 °C (Fig. 3B). This difference in melting temperature is consistent with a decrease in crystallinity caused by the installation of amides into the polymer backbone. The *T*_m_ of PUU 4 was determined to be 91.5 °C by DSC, indicating that it could be melt processed.

Because polymer 2a contains amide linkages, we envisioned that it would be adhesive to nylon-6,6. To this end, we analyzed the adhesion of amide-PE 2a to nylon-6,6 by lap-shear tests (Fig. 3C). The lap-shear tests indicated that polymer 2a was more adhesive to nylon-6,6 (2.62 ± 0.15 MPa) than was unmodified LDPE (0.31 ± 0.04 MPa). As a comparison to widely used commercial glues, the adhesion strength of Gorilla Glue to nylon-6,6 (0.65 ± 0.24 MPa) was evaluated under identical conditions and found to be less adhesive to the nylon substrate than was polymer 2a (Fig. S43†).

We also measured the adhesion of amide-LDPE 2a to aluminum and glass. When used as an interlayer between aluminum or glass substrates, unmodified LDPE was not sufficiently adhesive to create a testable sample, whereas polymer 2a adhered to both aluminum and glass (5.87 ± 0.33 MPa and 10.9 ± 0.90 MPa respectively). The adhesion of polymer 2a to both aluminum and glass is stronger than the adhesion of commercial Gorilla Glue to both substrates (4.30 ± 0.90 MPa and 6.54 ± 1.95 MPa respectively) under identical conditions (Fig. S43†). In addition, the adhesion of polymer 2a to glass is stronger than the adhesion of some epoxy resins.^40,41^ These adhesion data suggest that the installation of amide linkages into the backbone of polyethylene can create polymers with adhesive properties that are enhanced over those of unmodified LDPE. PUU 4 was moderately adhesive to nylon-6,6, aluminum, and glass (1.17 ± 0.22 MPa, 0.76 ± 0.05 MPa, 0.65 ± 0.01 and MPa respectively), indicating that the surface properties of materials derived from LDPE also can be more favorable than those of unmodified LDPE.

We also investigated the mechanical properties of amide-PE 2a and PUU 4 by tensile tests (Fig. 3D). The elongation at break (*ε*_B_), tensile strength (*σ*_B_), toughness (*U*_T_), and Young's modulus (*E*) of 2a were 303.9 ± 75.0%, 11.2 ± 1.4 MPa, 27.2 ± 7.2 MJ m^−3^, and 57.0 ± 10.2 MPa respectively. These values for *ε*_B,_*U*_T_, and *σ*_B_ are similar to the values of unmodified LDPE (227.8 ± 96.8%, 20.9 ± 9.8 MJ m^−3^, 11.4 ± 1.1 MPa respectively). However, the *E* of polymer 2a (57.0 ± 10.2 MPa) was significantly lower than that of unmodified LDPE (148.5 ± 16.8 MPa), presumably because of the defects in the crystalline regions caused by the amide linkages in 2a. Overall, the bulk properties of the polymer are largely retained after incorporation of an in-chain amide linkage. However, when PUU 4 was subjected to tensile testing, the *E* and *σ*_B_ of PUU 4a (0.9 ± 0.2 MPa and 5.5 ± 0.9 MPa respectively) were found to be significantly different from the values of unmodified LDPE (148.5 ± 16.8 MPa and 11.4 ± 1.1 MPa respectively) and polymer 2a (57.0 ± 10.2 MPa and 11.2 ± 1.4 MPa respectively). The *ε*_B_ of PUU 4 (939.2 ± 89.9%) was found to be much greater than the *ε*_B_ of LDPE (227.8 ± 96.8%) and polymer 2a (303.9 ± 75.0%), while the *U*_T_ of PUU 4 (29.9 ± 6.6 MJ m^−3^) was similar to the *U*_T_ of LDPE (20.9 ± 9.8 MJ m^−3^) and of polymer 2a (27.2 ± 7.2 MJ m^−3^). Elastic hysteresis curves of PUU 4 verified the elastic nature of this material containing the harder polyethylene and softer polyether units (see Fig. S50†). In contrast, PU 4′, which was synthesized in the absence of polyethylene fragments, was isolated as an amorphous solid and was not suitable for mechanical testing. This result indicates that the telechelic polyethylene fragments serve as hard segments to add rigidity to PUU 4. The wide range of bulk and surface properties possessed by these new polymers highlight the ability to create valuable materials by selective chemical transformations of polyolefins.

## Conclusions

In conclusion, we have demonstrated a strategy to integrate the pendent functional groups installed onto polyethylene by C–H functionalization into the backbone C–C bonds of the polymer to furnish cleavable linkages. We developed routes to polyamides from oxidized polyethylenes by Beckmann rearrangement of the intermediate oximes. These polyamides have enhanced surface properties, relative to unmodified polyethylene, while maintaining similar mechanical properties, and they underwent reductive cleavage at the amide linkages to afford telechelic fragments by ruthenium-catalyzed hydrogenolysis. The resulting telechelic units were then polymerized to form PUU elastomers. This work points to strategies that could lower the barriers to reuse of polyethylene and increase the sustainability of hydrocarbon-based plastics.

## Author contributions

Conceptualization: J. X. S. and J. F. H. methodology: J. X. S., N. R. C., and J. F. H. investigation of methodology: J. X. S., D. D. K., N. R. C., and P. L. B. writing – original draft: J. X. S., D. D. K., and J. F. H. writing – review and editing: J. X. S., N. R. C., and J. F. H. with contributions and approval from all authors.

## Conflicts of interest

There are no conflicts to declare.

## Supplementary Material

SC-016-D5SC02684A-s001

## Data Availability

The data supporting this article have been included as part of the ESI.†
