# Supplementary material for: Backbone editing and deconstruction of polyethylene by Beckmann rearrangement and hydrogenolysis
Source: Chem Sci. 2025 Jun 2;16(25):11304–10. doi: 10.1039/d5sc02684a (PMC12128105; doi:10.1039/d5sc02684a)
Supplement: SC-016-D5SC02684A-s001 [file SC-016-D5SC02684A-s001.pdf]

Supplementary Information (SI) for Chemical Science.
This journal is © The Royal Society of Chemistry 2025

Supporting Information for

Backbone Editing and Deconstruction of Polyethylene by Beckmann Rearrangement and Hydrogenolysis

Jake X. Shi,[‡,a,b] Diane D. Kim,[‡,a] Nicodemo R. Ciccia, [‡a,b] Pierre Lahaie-Boivin,[a] and John F. Hartwig[a,b*]

[‡]These authors contributed equally to this work

[a]Department of Chemistry, University of California, Berkeley, California 94720, United States

[b]Division of Chemical Sciences, Lawrence Berkeley National Laboratory, Berkeley, California 94720, United States

Table of Contents

General Information 1
Assessment of the degree of functionalization 2
Calculation of the stoichiometry of catalysts and reagents 2
Calculation of yield 2
Synthesis of polymers 3
Synthesis of small-molecule models 7
Beckmann rearrangement of small-molecule models 8
Hydrogenolysis of polymers 10
Synthesis of polyurea-urethane 4 12
Calculation of wt% PE fragments in polyurea–urethane 4 13
Synthesis of polyurethane 4' 14
Characterization of compounds 15
Additional NMR Spectra 55
Materials testing 56
References 69

General Information

All air sensitive manipulations were conducted under an inert atmosphere in a nitrogen-filled or argon-filled glovebox or by standard Schlenk techniques. All reagents were purchased from commercial sources and used without further purification. Low density polyethylene (LDPE) and -high density polyethylene (HDPE), were purchased from Sigma-Aldrich. Solvents were degassed with nitrogen and dried in a solvent purification system with a 1 m column containing activated alumina and stored under 4Å molecular sieves. Fourier-transform infrared spectra were collected using a Bruker Vortex 80 spectrometer. Room-temperature NMR spectra were collected using 400, 500, and 600 MHz Bruker Instruments at the University of California, Berkeley. Variable-temperature NMR spectroscopic analysis was conducted on the 500 and 600 MHz instruments at University of California Berkeley. ^{1}H chemical shifts were reported in ppm, relative to the resonance of the residual solvent ($CDCl_3$, 7.26 ppm; $C_2D_2Cl_4$, 6.00 ppm). ^{13}C chemical shifts were reported in ppm, relative to the resonance of the residual solvent ($CDCl_3$, 77.16 ppm; $C_2D_2Cl_4$, 73.78 ppm). High-temperature, size-exclusion chromatography (HT-SEC) was performed on a Tosoh EcoSEC-HT with three TSKgel GMHhr-H(S) HT columns in series. Runs were performed at 135 ºC and 1 mL/min with 1,2,4-trichlorobenzene + 0.05% butylated hydroxytoluene (BHT) as mobile phase. Molecular weight was determined relative to polyethylene standards. For polymers that were soluble in tetrahydrofuran at room temperature, size exclusion chromatography (SEC) was performed on a Malvern OmniSEC equipped with refractive index, light scattering, and intrinsic viscosity detectors and two Malvern T6000M columns in series. Runs were performed at 35 ºC and 1 mL/min with tetrahydrofuran as mobile phase. Absolute molecular weight was calculated following detector calibration with a single narrow poly(styrene) standard. Differential scanning calorimetry (DSC) was performed on a TA Discovery DSC 25 instrument. Aluminum 6061 (Al-6061) and nylon-6,6 (ASTM D5989) substrates were cut at the UC Berkeley Cory Hall Machine shop from 0.160 cm thick, 10.16 cm x 121.92 cm (0.063" thick, 4"x48") and from 0.635 cm thick, 15.24 cm x 121.92 cm (0.25" thick, 6"x48") sheet stocks, respectively, purchased from McMaster-Carr (USA). Lap shear adhesion testing was conducted according to ASTM D1002-10 on an Instron universal materials tester equipped with a 5 kN load cell with a shear rate of 1.5 mm/min. Adhesion strength was determined by the maximum load divided by the bonded overlap area, which was measured with digital calipers prior to testing, and the apparent failure mode was assessed visually. The adhesive strengths of LDPE and functionalized polyethylenes to aluminum were assessed by single lap shear testing on rectangular aluminum 6061 (Al 6061) substrates with dimensions 0.16 cm thick x 1 cm width x 10 cm length. The adhesive strengths of LDPE and functionalized polyethylenes to nylon-6,6 was assessed by single lap shear testing on rectangular nylon-6,6 (ASTM D5989) substrates with dimensions 0.16 cm thick x 1 cm width x 10 cm length. Compression molding was conducted on a Carver benchtop lab press with heated plates (model 4386). Tensile testing was conducted according to ASTM D638 on an Instron universal materials tester. Tensile stress and strain were measured at room temperature using an extension rate of 50 mm/min. Thermogravimetric analysis (TGA) was performed with a TA Discovery TGA 550 instrument.

CAUTION: Heating solvents to temperatures above their boiling points in sealed containers creates high pressures and possible explosion hazards. Reactions conducted in sealed vials with solvents at temperatures above their boiling points should be performed with caution, such as behind a safety shield.

Assessment of the degree of functionalization

The degree of functionalization was determined by ^{1}H NMR spectroscopy at 100 °C in $C_2D_2Cl_4$. The integration of the peaks between 1.7 and 0.7 ppm was set to 400 (4 proton per monomer unit, 100 monomer units in total). The integration of the protons that are alpha to the functional groups of interest were then compared to the integration of the protons of the monomer units.

Calculation of the stoichiometry of catalysts and reagents

The stoichiometry for the catalysts and reagents were calculated based on the number of functional groups in the polymer.

$$mmol_{amide} = \frac{mass_{polymer}}{MW_{repeat\ unit}} * \frac{\%\ functionalization}{100} * 1000$$

For example, 1 g of polymer **2a** with 3.0% functionalization:

$$mmol_{amide} = \frac{1\ .0\ g}{\left(28.05 \frac{g}{mol}\right)} * \frac{3.0\%}{100} * 1000 = 1.1\ mmol_{amide}$$

Calculation of yield

The yield for each reaction was determined by the following equation in which $mass_{product,actual}$ denotes the mass of the polymer obtained after the reaction, and $mass_{product,theoretical}$ denotes the mass of the polymer if all of the initial functional groups have been functionalized.

$$\%\ mass\ yield = 100 * \left(\frac{mass_{product,actual}}{mass_{product,theoretical}}\right)$$

For example, for the synthesis of polymer **1a** by the condensation of polymer **1** with hydroxylamine hydrochloride:

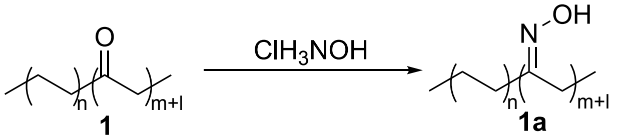

1
3.3 g
3.0% ketone
3.5 mmol ketone

1a
2.9 g

$$mass_{product,theoretical} = 3.3\ g + \frac{3.5\ mmol * MW_{NH}}{1000} = 3.4\ g$$

$$\%\ mass\ yield = 100 * \left(\frac{2.9\ g}{3.4\ g}\right) = 85\%$$

Synthesis of polymers
***Oxo*-PE**

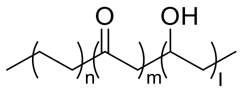

The synthesis of *oxo*-PE was adapted from a literature procedure.[1]

To a 250 mL round-bottom flask with a stir bar, 3.00 g of PE (LDPE, HDPE, or waste HDPE (milk jug), 107 mmol) were added. The solids were suspended in 107 mL of 1,2-dichlorobenzene. The flask was heated at 120 °C until the polymer dissolved. Then, 1.77 g of 2,6-dichloropyridine-*N*-oxide (10.7 mmol) were added to the flask. In a separate 4 mL vial, 5.9 mg of tetrakispentafluorophenylphorphyrin ruthenium carbonyl (0.0054 mmol) were dissolved in 5.3 mL of dichloromethane. To the flask heated at 120 °C, the solution containing the catalyst was added with a pipette in a portion wise manner (CAUTION: dichloromethane evaporates vigorously when the solution is added). The flask was heated at 120 °C for 1 h. The flask was cooled slightly, and the contents were poured in 300 mL of methanol under vigorous stirring to precipitate the polymer. The slurry was filtered, and the powder was washed with copious amounts of methanol to afford *oxo*-PE as a light tan powder. Mass recovery: LDPE: 2.9 g, 97%; HDPE: 3.0 g, >99%; Waste HDPE: 2.9 g, 97%. Molar yield with respect to 2,6-dichloropyridine-*N*-oxide: LDPE: 33%; HDPE: 38%; Waste HDPE: 27%.

LDPE: The degree of functionalization was determined to be 3.3% total functionalization (2.4% ketone and 0.9% alcohol) by ^{1}H NMR spectroscopy at 100 °C. ^{1}H NMR (600 MHz, $C_2D_2Cl_4$) δ 3.62 (br, C**H**OH), 2.40 (t, *J* = 7.2 Hz, C**H**$_2$C(O)C**H**$_2$), 1.62 (br), 1.35 (br), 0.98 – 0.88 (m).

HDPE: The degree of functionalization was determined to be 3.8% total functionalization (2.5% ketone and 1.3% alcohol) by ^{1}H NMR spectroscopy at 100 °C. ^{1}H NMR (600 MHz, $C_2D_2Cl_4$) δ 3.62 (br, C**H**OH), 2.40 (t, *J* = 7.2 Hz, C**H**$_2$C(O)C**H**$_2$), 1.61 (br), 1.34 (br), 0.98 – 0.88 (m).

Waste HDPE (milk jug): The degree of functionalization was determined to be 2.7% total functionalization (1.9% ketone and 0.8% alcohol) by ^{1}H NMR spectroscopy at 100 °C. ^{1}H NMR (600 MHz, $C_2D_2Cl_4$) δ 3.62 (br, C**H**OH), 2.40 (t, *J* = 7.2 Hz, C**H**$_2$C(O)C**H**$_2$), 1.62 (br), 1.35 (br), 0.98 – 0.92 (m).

***Keto*-PE**

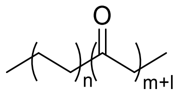

The synthesis of *keto*-PE was adapted from a literature procedure.[2]

To a 250 mL Corning bottle containing a stir bar under nitrogen, 3.0 g of *Oxo*-PE (LDPE, HDPE, or waste HDPE (milk jug), 1.3% alcohol, 1.4 mmol alcohol), 42 mg of pentamethylcyclopentadienyl iridium dichloride dimer (0.052 mmol), and 19 mg of potassium carbonate (0.14 mmol) were added. The solids were suspended in 120 mL of toluene, and 2 mL of acetone (27 mmol) were added. The bottle was tightly sealed and heated at 140 °C for 48 h. The bottle was cooled slightly, and the contents were poured in 300 mL of methanol under vigorous stirring to precipitate the polymer. The slurry was filtered, and the powder was washed with copious amounts of methanol to afford *keto*-PE as a light tan powder. Mass recovery: LDPE: 2.8 g, 93%; HDPE: 2.7 g, 90%; Waste HDPE: 2.6 g, 87%.

LDPE: The degree of functionalization was determined to be 3.1% ketone by ^{1}H NMR spectroscopy at 100 °C. ^{1}H NMR (600 MHz, $C_2D_2Cl_4$) δ 2.40 (t, *J* = 7.2 Hz, C**H**$_2$C(O)C**H**$_2$), 1.62 (br), 1.35 (br), 0.98 – 0.88 (m).

HDPE: The degree of functionalization was determined to be 3.8% ketone by ^{1}H NMR spectroscopy at 100 °C. ^{1}H NMR (600 MHz, $C_2D_2Cl_4$) δ 2.40 (t, *J* = 7.2 Hz, C**H**$_2$C(O)C**H**$_2$), 1.62 (br), 1.34 (br), 0.98 – 0.92 (m).

Waste HDPE (milk jug): The degree of functionalization was determined to be 2.8% ketone by ^{1}H NMR spectroscopy at 100 °C. ^{1}H NMR (600 MHz, $C_2D_2Cl_4$) δ 2.40 (t, *J* = 7.2 Hz, C**H**$_2$C(O)C**H**$_2$), 1.62 (br), 1.35 (br), 0.98 – 0.92 (m).

***Oxime*-PE**

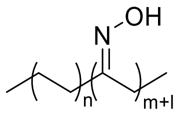

The synthesis of *oxime*-PE was adapted from a literature procedure.[2] As previously reported, the *oxime*-PEs were not able to be characterized by HTSEC because no peak was observed in the chromatograms; we posit that this observation could be caused by aggregation of the oximes in 1,2,4- trichlorobenzene at 135 °C which prevented elution and detection of the polymer.

For LDPE: To a 250 mL Corning bottle with a stir bar, 3.28 g of *keto*-LDPE (3% ketone, 3.51 mmol ketone) and 1.22 g of hydroxylamine hydrochloride (17.6 mmol) were added. The solids were suspended in 110 mL of pyridine. The bottle was heated at 100 °C for 12 h. The bottle was cooled slightly, and the contents were poured in 300 mL of methanol under vigorous stirring to precipitate the polymer. The slurry was filtered, and the powder was washed with copious amounts of methanol to afford *oxime*-LDPE as a light tan powder (2.85 g, 86%)

For HDPE and waste-HDPE: To a 4 mL vial with a stir bar, 25 mg of *keto*-HDPE (3% ketone, 0.027 mmol ketone) and 10 mg of hydroxylamine hydrochloride (0.14 mmol) were added. The solids were suspended in 1 mL of pyridine. The vial was heated at 120 °C for 3 h and 30 min. The vial was cooled slightly, and 3 mL of methanol were added to the vial under vigorous stirring to precipitate the polymer. The slurry was filtered, and the powder was washed with copious amounts of methanol to afford *oxime*-HDPE as a light tan powder. Mass recovery: HDPE: 21 mg, 84%; Waste HDPE: 23 g, 92%.

LDPE: The degree of functionalization was determined to be 3.1% oxime by ^{1}H NMR spectroscopy at 100 °C. ^{1}H NMR (600 MHz, $C_2D_2Cl_4$) δ 2.38 (t, *J* = 7.8 Hz, C**H**2CNOHCH2), 2.21 (t, *J* = 7.2 Hz, CH_2CNOHC$\mathbf{H}_2$), 1.57 (br), 1.34 (br), 0.98 – 0.88 (m).

HDPE: The degree of functionalization was determined to be 3.5% oxime by ^{1}H NMR spectroscopy at 100 °C. ^{1}H NMR (600 MHz, $C_2D_2Cl_4$) δ 2.38 (t, *J* = 7.8 Hz, C**H**2CNOHCH2), 2.21 (t, *J* = 7.2 Hz, CH_2CNOHC$\mathbf{H}_2$), 1.57 (br), 1.35 (br), 0.98 – 0.88 (m).

Waste HDPE (milk jug): The degree of functionalization was determined to be 2.4% oxime by ^{1}H NMR spectroscopy at 100 °C. ^{1}H NMR (600 MHz, $C_2D_2Cl_4$) δ 2.38 (t, *J* = 7.8 Hz, C**H**2CNOHCH2), 2.21 (t, *J* = 7.2 Hz, CH_2CNOHC$\mathbf{H}_2$), 1.57 (br), 1.34 (br), 0.98 – 0.92 (m).

Amide-**PE**

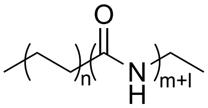

For LDPE: To a 250 mL Corning bottle with a stir bar, 1.00 g of *oxime*-LDPE (3% oxime, 3.2 mmol oxime) were added. The solids were suspended in 100 mL of THF. The flask was heated at 80 °C until the polymer dissolved. The bottle was cooled to room temperature, and 5.7 mL of a propanephosphonic acid anhydride in ethyl acetate (1.7 M, 9.6 mmol) solution were added to the bottle. The bottle was tightly sealed and heated at 80 °C for 24 h. The bottle was cooled slightly, and the contents were poured in 300 mL of methanol under vigorous stirring to precipitate the polymer. The slurry was filtered, and the powder was washed with copious amounts of methanol to afford *amide*-LDPE as a light tan powder (830 mg, 83%).

For HDPE and waste-HDPE: To a 20 mL vial with a stir bar, 75 mg of *oxime*-HDPE (3% oxime, 0.081 mmol oxime) were added. The solids were suspended in 3 mL of THF. The vial was heated at 80 °C until the polymer dissolved. The vial was cooled to room temperature, and 144 μL of a propanephosphonic acid anhydride in ethyl acetate (1.7 M, 0.24 mmol) solution were added to the vial. The vial was tightly sealed and heated at 120 °C for 5 min and then at 80 °C for 24 h. The vial was cooled slightly, and 16 mL of methanol was added to the vial under vigorous stirring to precipitate the polymer. The slurry was filtered, and the powder was washed with copious amounts of methanol to afford *amide*-HDPE as a light tan powder. Mass recovery: HDPE: 60 mg, 80%; Waste HDPE: 54 mg, 72%. The *amide*-HDPEs were not able to be characterized by HTSEC because no peak was observed in the chromatograms; we posit that this observation could be caused by aggregation of the amides in 1,2,4-trichlorobenzene at 135 °C which prevented elution and detection of the polymer.

LDPE: The degree of functionalization was determined to be 2.2% amide by ^{1}H NMR spectroscopy at 100 °C. ^{1}H NMR (600 MHz, $C_2D_2Cl_4$) δ 5.28 (br, $CH_2C(O)N\mathbf{H}CH_2$), 3.30 – 3.20 (m, $CH_2C(O)NHC\mathbf{H}_2$), 2.17 (t, J = 7.5 Hz, $C\mathbf{H}_2C(O)NHCH_2$), 1.66 (m), 1.54 (br), 1.35 (br), 0.98 – 0.88 (m). ^{13}C NMR (151 MHz, $C_2D_2Cl_4$) δ 172.4 ($CH_2\mathbf{C}(O)NHCH_2$), 42.5, 39.4, 37.5, 36.6, 35.1, 33.9, 33.5, 32.1, 31.6, 30.1, 29.9, 29.6, 29.4, 29.4, 29.3, 29.3, 29.2, 29.1, 29.1, 29.0, 28.9, 28.5, 26.7, 26.7, 26.3, 25.5, 23.8, 23.4, 23.3, 22.8, 22.3, 13.7, 13.7.

HDPE: The degree of functionalization was determined to be 3.0% amide by ^{1}H NMR spectroscopy at 100 °C. ^{1}H NMR (600 MHz, $C_2D_2Cl_4$) δ 5.29 (br, $CH_2C(O)N\mathbf{H}CH_2$), 3.30 – 3.20 (m, $CH_2C(O)NHC\mathbf{H}_2$), 2.17 (t, J = 7.5 Hz, $C\mathbf{H}_2C(O)NHCH_2$), 1.66 (m), 1.54 (br), 1.35 (br), 1.09 (m), 0.98 – 0.88 (m).

Waste HDPE (milk jug): The degree of functionalization was determined to be 2.5% amide by ^{1}H NMR spectroscopy at 100 °C. ^{1}H NMR (600 MHz, $C_2D_2Cl_4$) δ 5.26 (br, $CH_2C(O)N\mathbf{H}CH_2$), 3.30 – 3.20 (m, $CH_2C(O)NHC\mathbf{H}_2$), 2.17 (t, J = 7.5 Hz, $C\mathbf{H}_2C(O)NHCH_2$), 1.66 (m), 1.54 (br), 1.35 (br), 1.24 (m), 1.10 (m), 0.98 – 0.88 (m).

Synthesis of small-molecule models

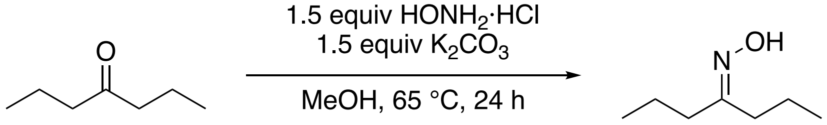

oxime-7

4-Heptanone oxime (***oxime-7)*****:** To a 20 mL scintillation vial was added 418 µL 4-heptanone (3.0 mmol, 1.0 equiv), 313 mg $HONH_2$·HCl (4.5 mmol, 1.5 equiv), 622 mg K_2CO_3 (4.5 mmol, 1.5 equiv), and 6 mL MeOH. The reaction mixture was heated at 65 °C for 24 h. Then, the solution was diluted with EtOAc and washed with H_2O (3 x 50 mL) and brine (50 mL). The organic phase was dried over Na_2SO_4, and the solvent was removed under vacuum to yield the title compound as a colorless oil (338 mg, 87% yield). The ^{1}H NMR spectrum of the title compound matches those previously reported.[3]

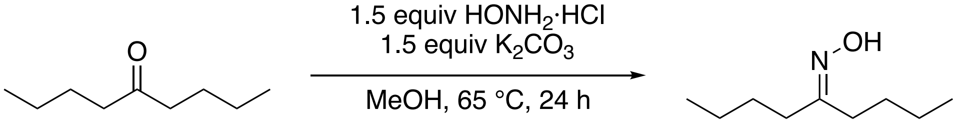

oxime-9

5-Nonanone oxime (***oxime-9)***: To a 20 mL scintillation vial was added 520 µL 5-nonanone (3.0 mmol, 1.0 equiv), 313 mg $HONH_2$·HCl (4.5 mmol, 1.5 equiv), 622 mg K_2CO_3 (4.5 mmol, 1.5 equiv), and 6 mL MeOH. The reaction mixture was heated at 65 °C for 24 h. Then, the solution was diluted with EtOAc and washed with H_2O (3 x 50 mL) and brine (50 mL). The organic phase was dried over Na_2SO_4, and the solvent was removed under vacuum to yield the title compound as a colorless oil (446 mg, 95% yield). The ^{1}H NMR spectrum of the title compound matches those previously reported.[4]

Beckmann rearrangement of small-molecule models

General Procedure: The Beckmann rearrangement with propanephosphonic acid anhydride was conducted with small-molecule models of polyethylene to determine whether exchange between amide groups under the reaction conditions. To a 4 mL vial was added oxime (0.08 mmol, 1 equiv), 0.24 mmol propanephosphonic acid anhydride (144 µL of a 1.7 M solution in ethyl acetate, 3 equiv), and 1 mL THF. The reaction mixture was heated at 80 °C for 24 h. The reaction mixture was analyzed by GCMS to assess the formation of exchange products. Representative GC traces of the reaction mixtures showing the product distribution are shown in Scheme S1. No products from exchange reactions were observed by GCMS.

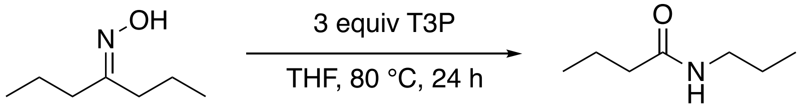

oxime-7

The reaction was performed according to the general procedure with 0.08 mmol 4-heptanone oxime (10 mg, 1 equiv), 0.24 mmol propanephosphonic acid anhydride (144 µL of a 1.7 M solution in ethyl acetate, 3 equiv), and 1 mL THF. A representative GC trace of the crude reaction mixture is shown in Scheme S1.

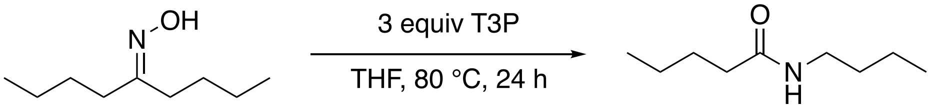

oxime-9

The reaction was performed according to the general procedure with 0.08 mmol 5-nonanone oxime (13 mg, 1 equiv), 0.24 mmol propanephosphonic acid anhydride (144 µL of a 1.7 M solution in ethyl acetate, 3 equiv), and 1 mL THF. A representative GC trace of the crude reaction mixture is shown in Scheme S1.

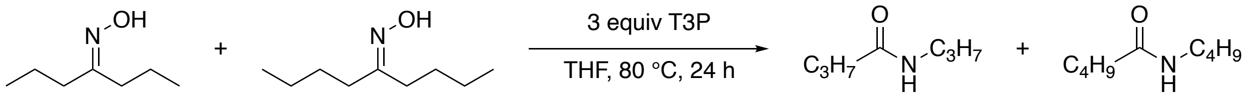

oxime-7 0.5 equiv ***oxime-9*** 0.5 equiv

The reaction was performed according to the general procedure with 0.04 mmol 4-heptanone oxime (5 mg, 0.5 equiv), 0.04 mmol 5-nonanone oxime (6 mg, 0.5 mmol), 0.24 mmol propanephosphonic acid anhydride (144 µL of a 1.7 M solution in ethyl acetate, 3 equiv), and 1 mL THF. A representative GC trace of the crude reaction mixture is shown in Scheme S1.

Scheme S1. Representative GCMS traces of the crude reaction mixtures of the Beckmann rearrangements of small-molecule models.

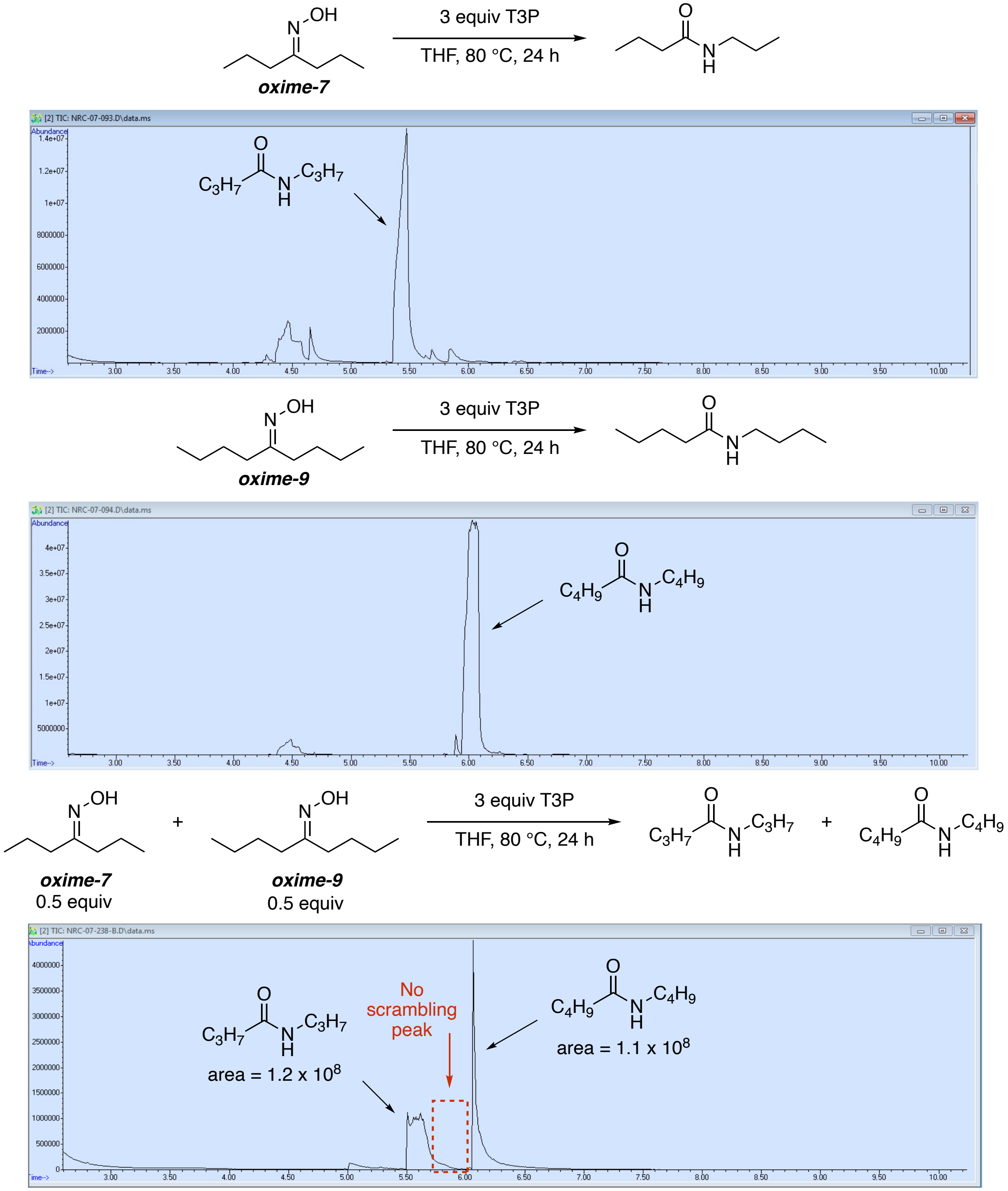

Hydrogenolysis of polymers
Synthesis of catalysts

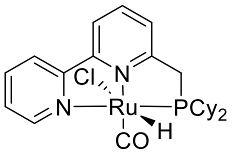
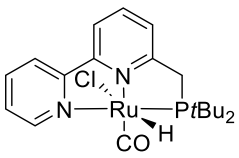
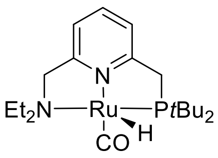
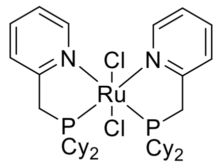
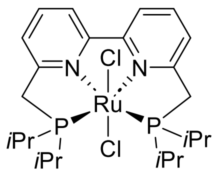

Ru1 **Ru2** **Ru3** **Ru4** **Ru5**

Complex **Ru1** was synthesized according to literature procedure.[5] Complex **Ru2** was synthesized according to literature procedure.[6] Complex **Ru3** was synthesized according to literature procedure.[7] Complex **Ru4** was synthesized according to literature procedure.[8] Complex **Ru5** was synthesized according to literature procedure.[9]

General procedure for the hydrogenolysis of *amide*-PE with ruthenium catalysts

To a 20 mL glass liner with a stir bar were added 50 mg of *amide*-PE (2.0% amide, 0.036 mmol), a corresponding amount of ruthenium catalyst, and a corresponding amount of base. The solids were suspended in 2 mL of solvent. The glass liner was placed in a Parr reactor (25 mL internal volume), and the reactor was sealed under nitrogen. The reactor was charged with hydrogen and heated for an allotted time. The reactor was then cooled to room temperature, and the reactor was depressurized slowly and opened. The liner was reheated to dissolve the polymer, and methanol was added to precipitate the polymer. The slurry was filtered to afford the product. The extent of hydrogenolysis of the amide linkages was assessed by ^{1}H NMR spectroscopy. The conditions and result for each reaction performed are summarized in **Table S1**.

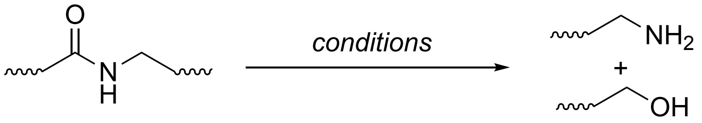

Table S1. Conditions for the hydrogenolysis of *amide*-LDPE with ruthenium catalysts.

Entry	Catalyst[a]	P_{H2} (bar)	Temp (°C)	Base	Conversion (%)[f]
1[b,d]	4 mol% **Ru1**	50	120	20 mol% KOtBu	52
2[b,d]	4 mol% **Ru2**	50	120	20 mol% KOtBu	n.r.
3[b,d]	4 mol% **Ru3**	50	120	20 mol% KOtBu	86
4[c,e]	5 mol% **Ru4**	80	150	20 mol% 2-methyl-2-adamantanol, 20 mol% NaH	14
5[c,d]	5 mol% **Ru5**	40	130	20 mol% NaH	15
6[c,e]	5 mol% **Ru5**	40	130	20 mol% NaH	>99

[a]Catalyst loading with respect to equivalents of amide. [b]Reaction run in THF. [c]Reaction run in PhMe. [d]Reaction run for 24 h. [e]Reaction run for 48 h. [f]Conversion of amides as determined by ^{1}H NMR spectroscopy in $CDCl_3$ at room temperature.

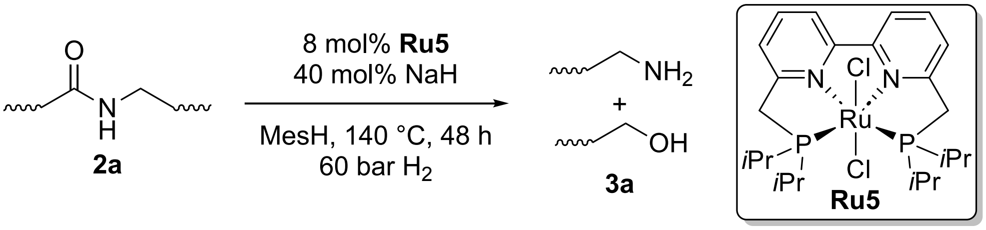

Procedure for the large scale hydrogenolysis of *amide*-LDPE with Ru5
To a 300 mL glass liner with a stir bar were added 1.5 g of *amide*-LDPE (2% amide, 1.1 mmol), 48 mg of **Ru5** (0.082 mmol), and 9.0 mg of sodium hydride (0.38 mmol). The solids were suspended in 40 mL of mesitylene. The bottle was placed in a Parr reactor (300 mL internal volume), and the reactor was sealed under nitrogen. The reactor was charged with 60 bar of hydrogen and heated at 140 °C for 48 h. The reactor was then cooled to room temperature, and the reactor was depressurized slowly and opened. The liner was reheated to dissolve the polymer, and methanol was added to precipitate the polymer. The slurry was filtered to afford the product as a brown powder. The extent of hydrogenolysis of the amide linkages was quantitative as assessed by ^{1}H NMR spectroscopy at 100 °C.

Hydrogenolysis of *amide*-LDPE: The degree of functionalization was determined to be 2.6% total functionalization (1.6% alcohol and 1.1% amine) by ^{1}H NMR spectroscopy at 100 °C. ^{1}H NMR (600 MHz, $C_2D_2Cl_4$) δ 3.66 (t, *J* = 6.6 Hz, C**H**$_2$OH), 3.05 (br, C**H**$_2$NH$_2$), 1.62 (br), 1.35 (br), 1.00 – 0.89 (m). ^{13}C NMR (151 MHz, $C_2D_2Cl_4$) δ 42.5, 33.9, 29.9, 29.4, 29.2, 29.1, 26.7, 23.8, 22.8, 22.4, 13.7. ^{13}C NMR (151 MHz, $C_2D_2Cl_4$) δ 62.8 (**C**H$_2$OH), 39.8 (**C**H$_2$NH$_2$), 37.5, 33.9, 33.5, 32.7, 32.1, 31.6, 29.9, 29.5, 29.4, 29.3, 29.2, 29.2, 29.0, 28.9, 28.8, 27.5, 26.7, 26.4, 25.6, 25.4, 22.8, 22.3, 13.7, 13.7.

Hydrogenolysis of *amide*-HDPE: The degree of functionalization was determined to be 4.1% total functionalization (2.3% alcohol and 1.8% amine) by ^{1}H NMR spectroscopy at 100 °C. ^{1}H NMR (600 MHz, $C_2D_2Cl_4$) δ 3.66 (t, *J* = 6.6 Hz, C**H**$_2$OH), 3.03 (br, C**H**$_2$NH$_2$), 1.62 (br), 1.35 (br), 1.00 – 0.92 (m).

Hydrogenolysis of *amide*-Waste HDPE (milk jug): The degree of functionalization was determined to be 2.1% total functionalization (1.4% alcohol and 0.7% amine) by ^{1}H NMR spectroscopy at 100 °C. ^{1}H NMR (600 MHz, $C_2D_2Cl_4$) δ 3.67 (t, *J* = 6.6 Hz, C**H**$_2$OH), 3.06 (br, C**H**$_2$NH$_2$), 1.62 (br), 1.35 (br), 1.00 – 0.91 (m).

Synthesis of polyurea-urethane 4

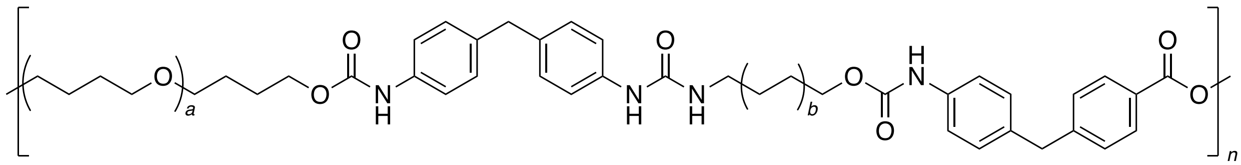

To a 20 mL vial with a stir bar under nitrogen were added 535 mg of poly(tetrahydrofuran) (M_n = 1000 Da, 0.54 mmol) and 161 mg of methylene diphenyldiisocyanate (0.64 mmol). The solids were suspended in 4 mL of THF, and the vial was heated at 80 °C for 3 h. The vial was cooled to room temperature. Then 75 mg of alcohol-amine-LDPE (0.11 mmol amine and alcohol) and 12.5 mg of tin(II) 2-ethylhexanoate (0.03 mmol) were added to the vial under nitrogen. The solids were further diluted with the addition of 4 mL of THF, and the vial was heated at 100 °C overnight. The viscous reaction mixture was poured into 200 mL of methanol under rigorous stirring to precipitate the polymer. The resulting solid was filtered and washed with copious amounts of methanol and dried under high vacuum at 80 °C overnight. The polymer was collected as a stringy yellow solid (664 mg). The polymer was unable to be characterized by SEC because of its insolubility in THF at 35 °C, DMF at 55 °C, or 1,2,4-trichlorbenzene at 135 °C.

^{1}H NMR (600 MHz, $C_2D_2Cl_4$) δ 7.32 (d, J = 8.0 Hz), 7.16 (d, J = 8.0 Hz), 6.56 (s), 4.22 (t, J = 6.8 Hz), 3.94 (br), 3.46 (br), 1.80 (br), 1.72 – 1.58 (m), 1.50 (br), 1.43 – 1.24 (m), 0.96 (br).

^{13}C NMR (151 MHz, $C_2D_2Cl_4$) δ 154.7, 153.5, 136.2, 136.1, 129.2, 119.2, 70.5, 70.4, 69.9, 65.1, 40.4, 29.4, 26.5, 26.2, 25.9.

Calculation of wt% PE fragments in polyurea–urethane 4

$$\# \, pTHF \; repeat \; units = \frac{H_\alpha}{4} = \frac{49.94}{4} = 12.49 \qquad (H_\alpha = 3.46 \, ppm)$$

$$\# \, PE \; repeat \; units = \frac{H_{PE}}{4} = \frac{26.32}{4} = \; 6.58 \qquad (H_{PE} = 1.34 \, ppm)$$

$$Referenced \; to \; 1 \; MDI \; unit: H_\beta = 4, H_{\beta'} = 4 \qquad (H_\beta = 7.33 \, ppm, H_{\beta'} = 7.15 \, ppm)$$

$$M_{avg,unit} \approx M_{pTHF,repeats}(\# \, pTHF \; repeats) + M_{PE,repeats}(\# \, PE \; repeats) + M_{MDI,average}$$

$$M_{avg,unit} \approx 72(12.49) + 28(6.58) + 283.3 = \; 1366.8 \; g/unit$$

$$wt\%_{PE,fragments} \approx \frac{M_{PE,repeats}(\# \, PE \; repeats)}{M_{avg,unit}}$$

$$wt\%_{PE,fragments} \approx \frac{28(6.58)}{1366.8} = 0.135 * 100 = 13.5 \; wt\%$$

Synthesis of polyurethane 4'

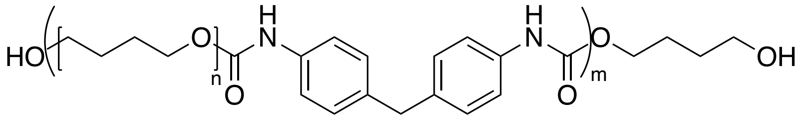

The title compound was prepared by a modified literature procedure.[10] To a 20 mL vial with a stir bar under nitrogen were added 641 mg of poly(tetrahydrofuran) (M_n = 1000 Da, 0.64 mmol) and 161 mg of methylene diphenyldiisocyanate (0.64 mmol). The solids were suspended in 5 mL of THF and one drop of tripropylamine was added. The vial was sealed and heated at 80 °C overnight. Then, the solvent was removed under vacuum at 80 °C. The remaining solid was dissolved in 10 mL DCM, filtered through a 0.2 µM syringe filter, and concentrated under vacuum. The title compound was collected as a colorless sticky solid (668 mg).

^{1}H NMR (500 MHz, $CDCl_3$) δ 7.27 (br), 7.09 (d, J = 8.4 Hz), 6.71 (br), 4.16 (t, J = 6.5 Hz), 3.87 (s), 3.63 (t, J = 5.8 Hz), 3.41 (br), 1.82 – 1.69 (m), 1.70 – 1.56 (m).

^{13}C NMR (126 MHz, $CDCl_3$) δ 153.9, 136.3, 136.2, 129.5, 119.0, 71.0, 70.8, 70.8, 70.3, 65.1, 62.9, 40.7, 30.5, 27.1, 26.6, 26.3, 26.0.

Characterization of compounds

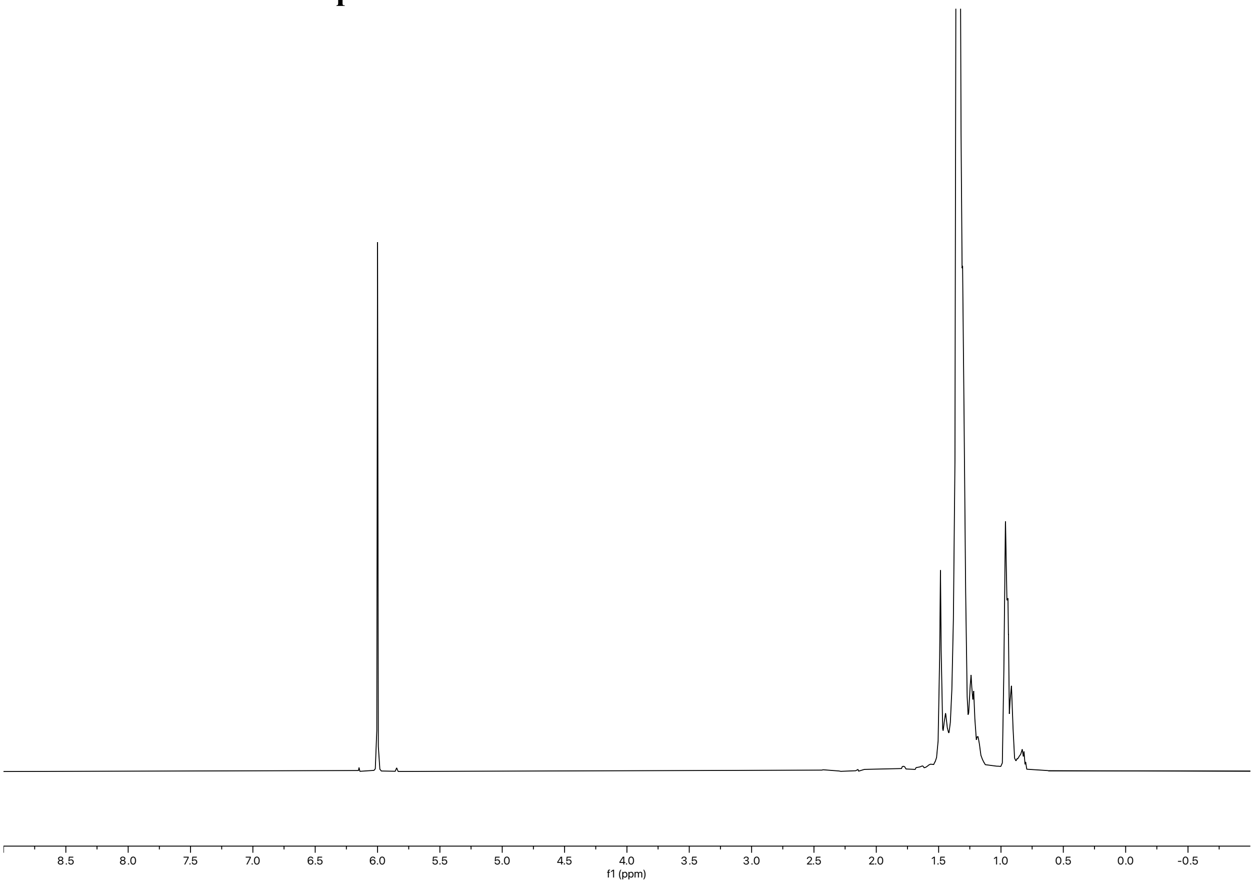

Figure S1. ^{1}H NMR spectrum of unmodified LDPE.

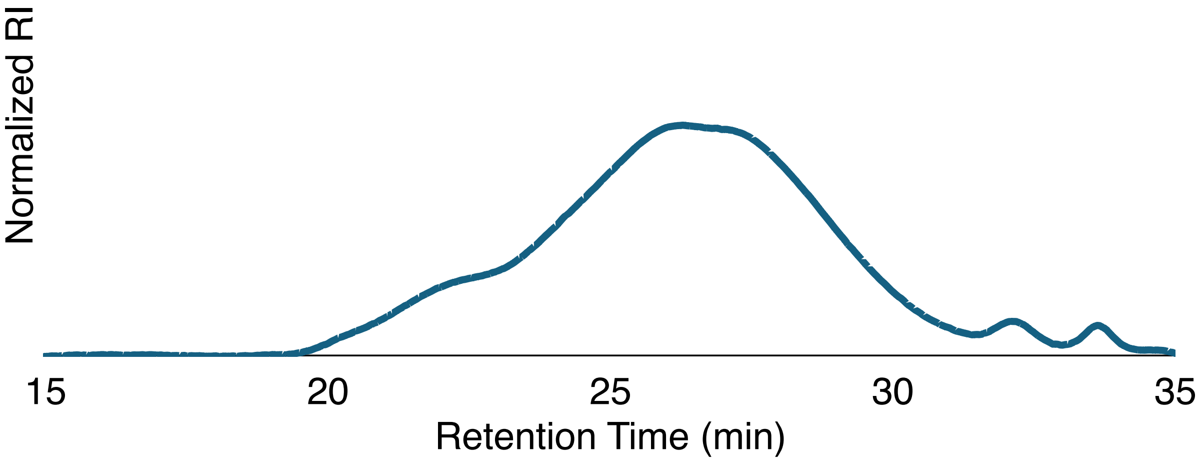

Figure S2. Size exclusion chromatogram of unmodified LDPE. M_n = 9.6 kDa, $Đ$ = 6.7. Molecular weight was determined relative to polyethylene standards.

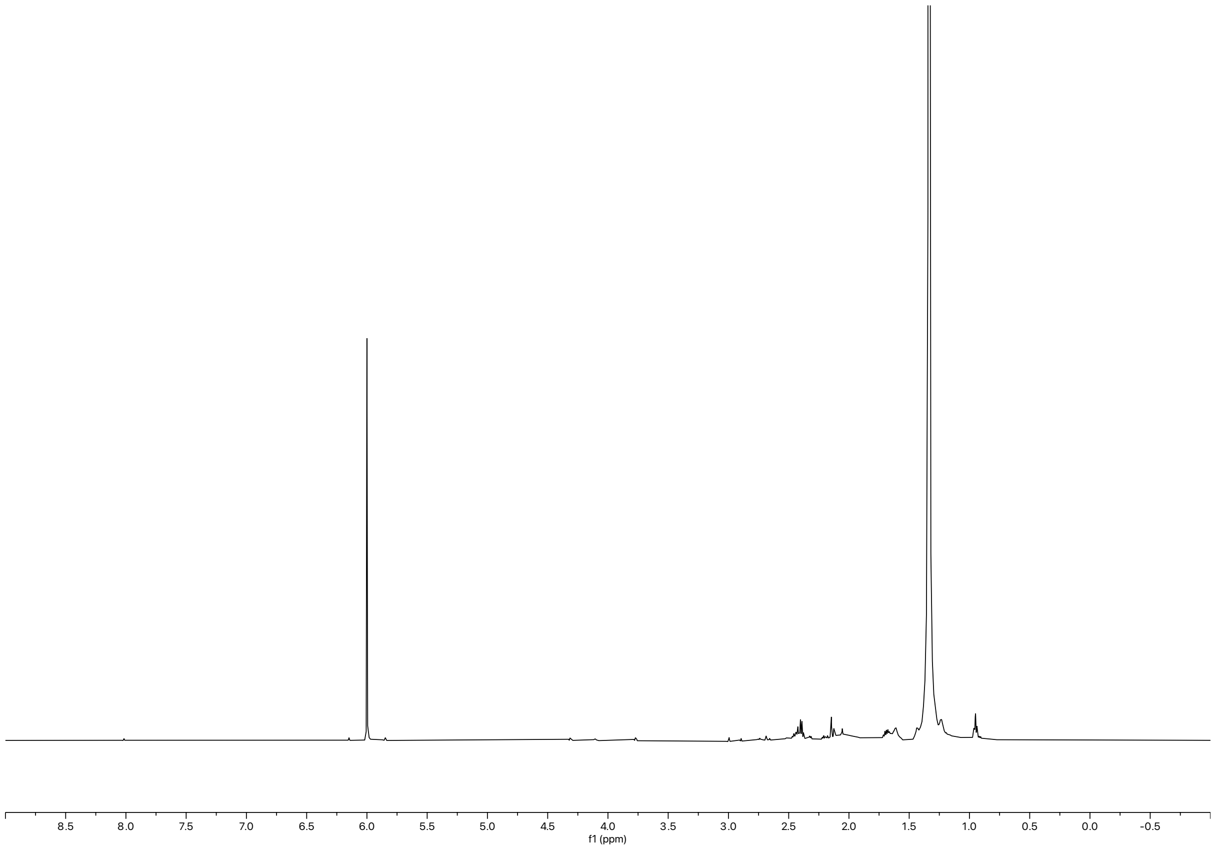

Figure S3. ^{1}H NMR spectrum of unmodified HDPE.

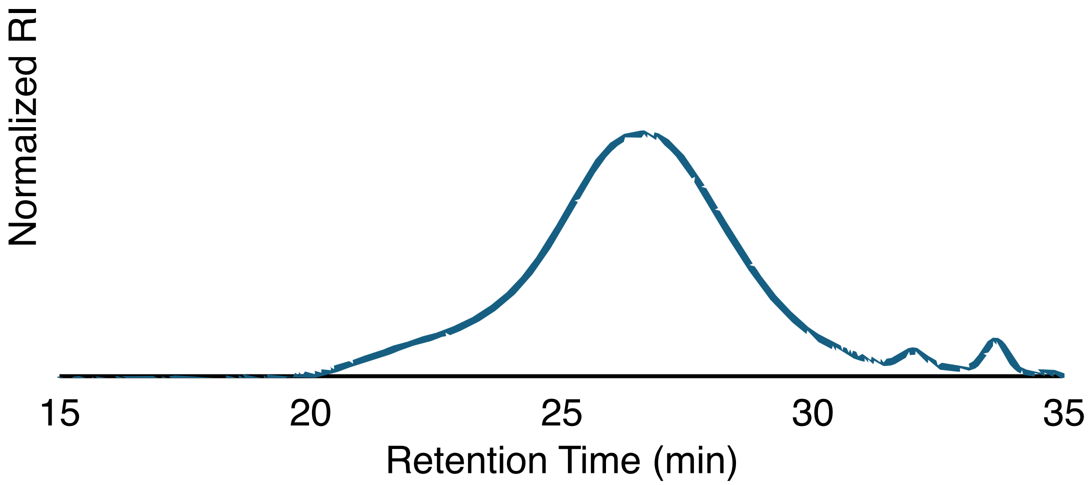

Figure S4. Size exclusion chromatogram of unmodified HDPE. M_n = 9.9 kDa, $Đ$ = 4.3. Molecular weight was determined relative to polyethylene standards.

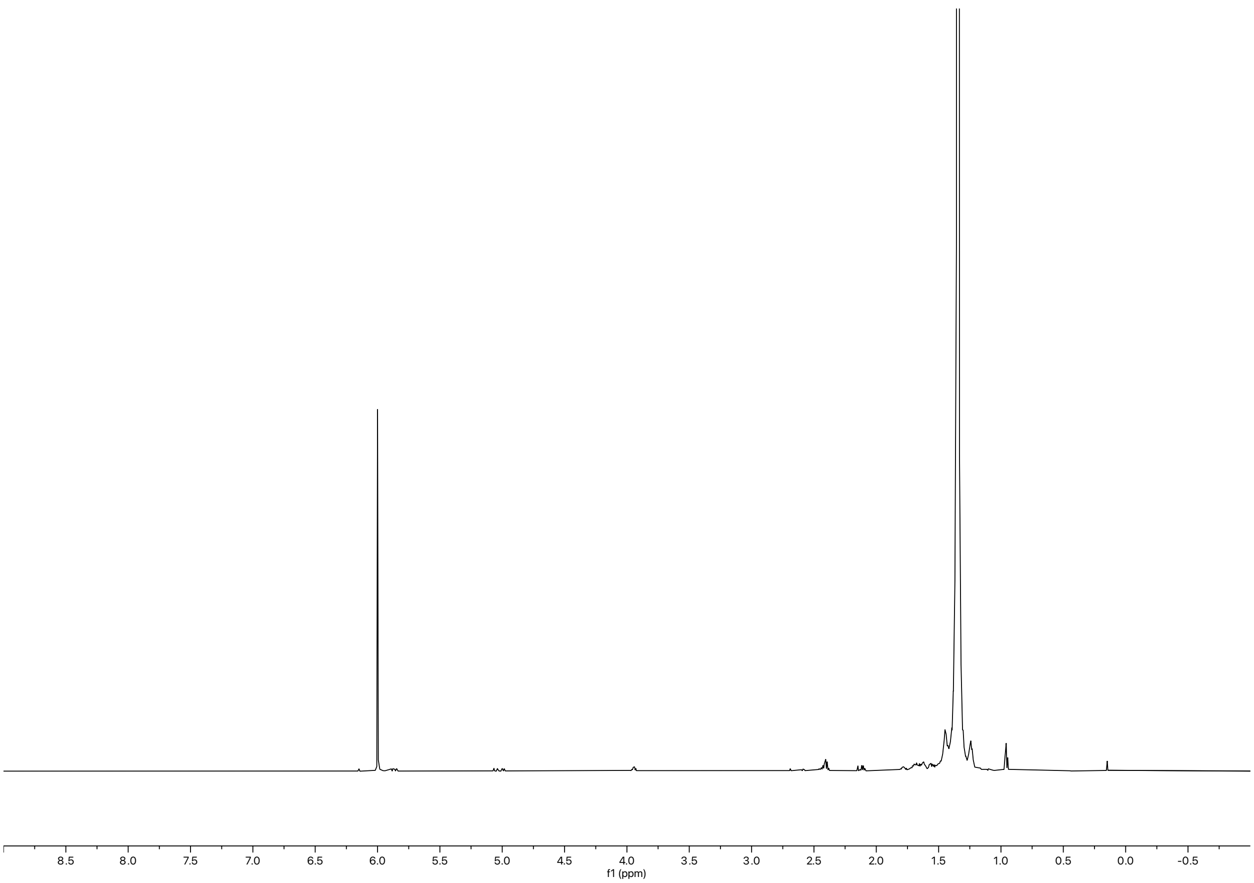

Figure S5. ^{1}H NMR spectrum of unmodified waste-HDPE (milk jug).

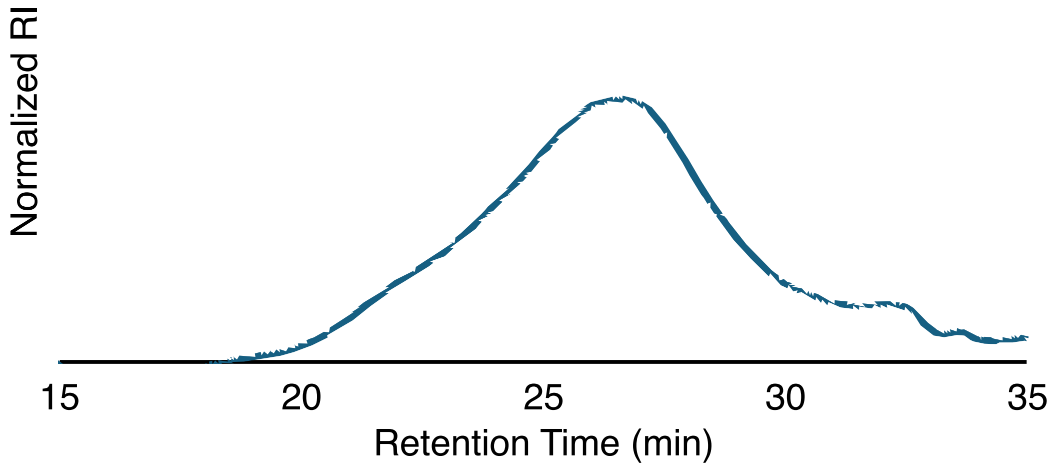

Figure S6. Size exclusion chromatogram of unmodified waste-HDPE (milk jug). M_n = 11.3 kDa, $Đ$ = 5.7. Molecular weight was determined relative to polyethylene standards.

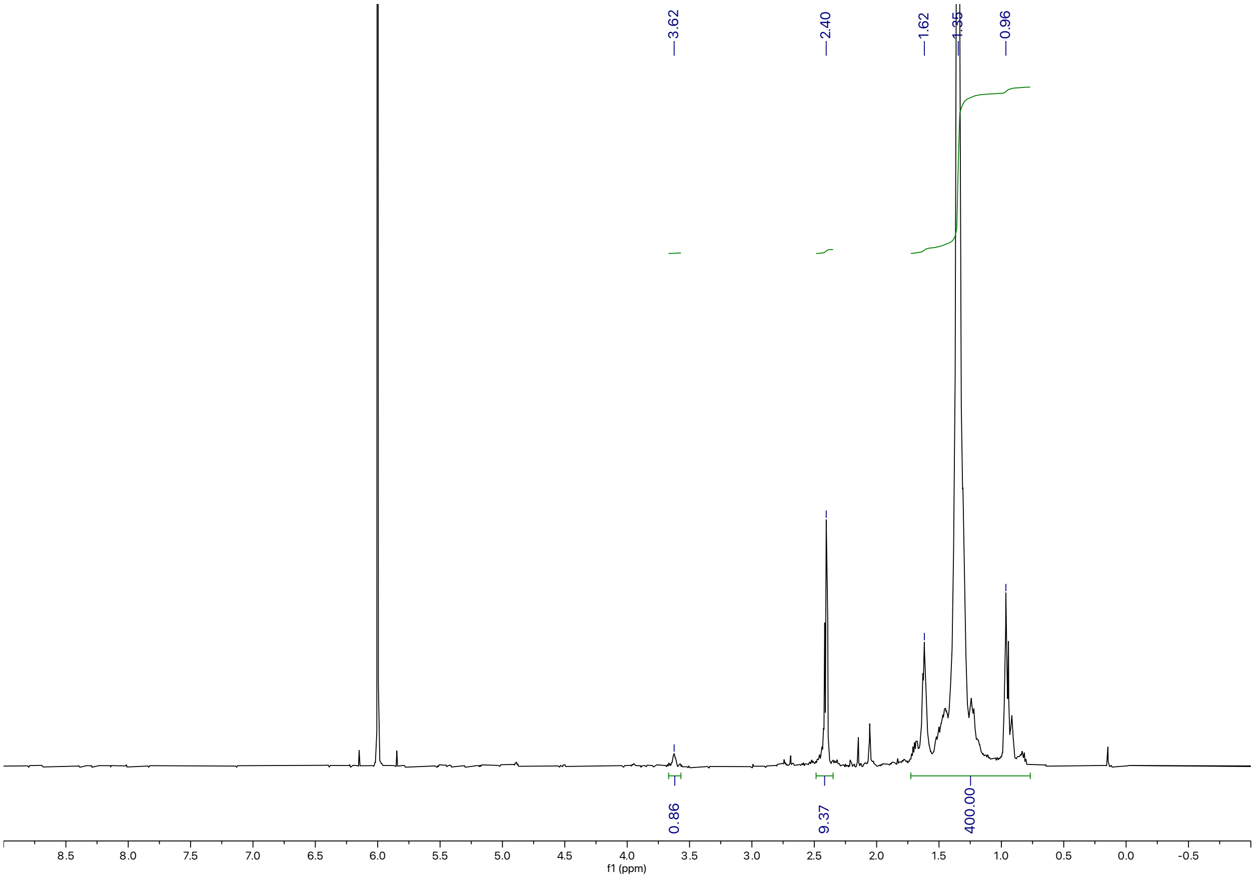

Figure S7. ^{1}H NMR spectrum of *oxo*-LDPE.

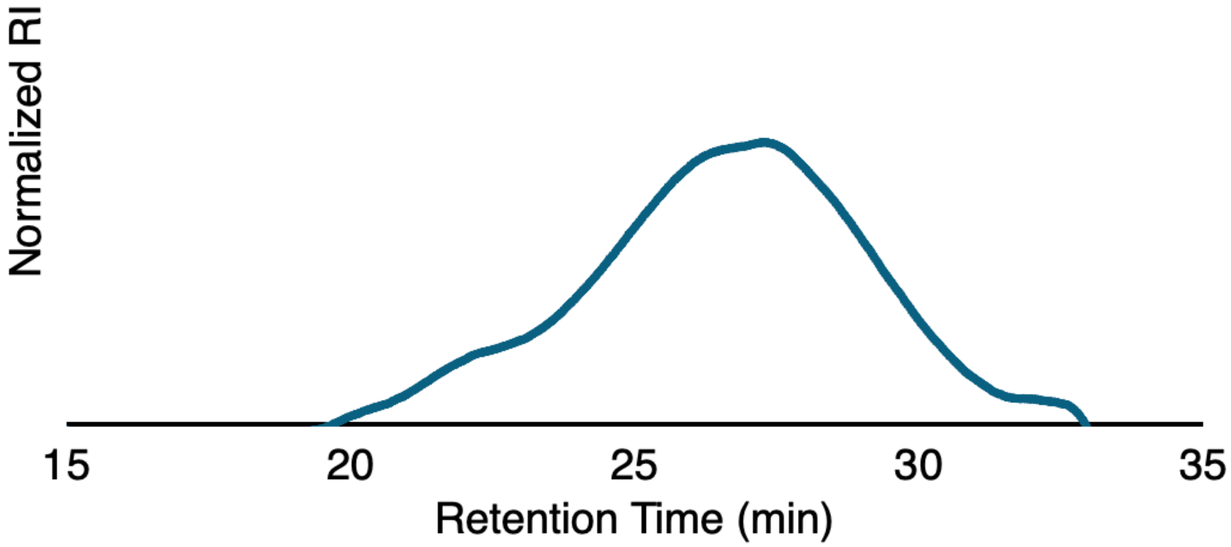

Figure S8. Size exclusion chromatogram of *oxo*-LDPE. M_n = 7.8 kDa, $Đ$ = 7.3. Molecular weight was determined relative to polyethylene standards.

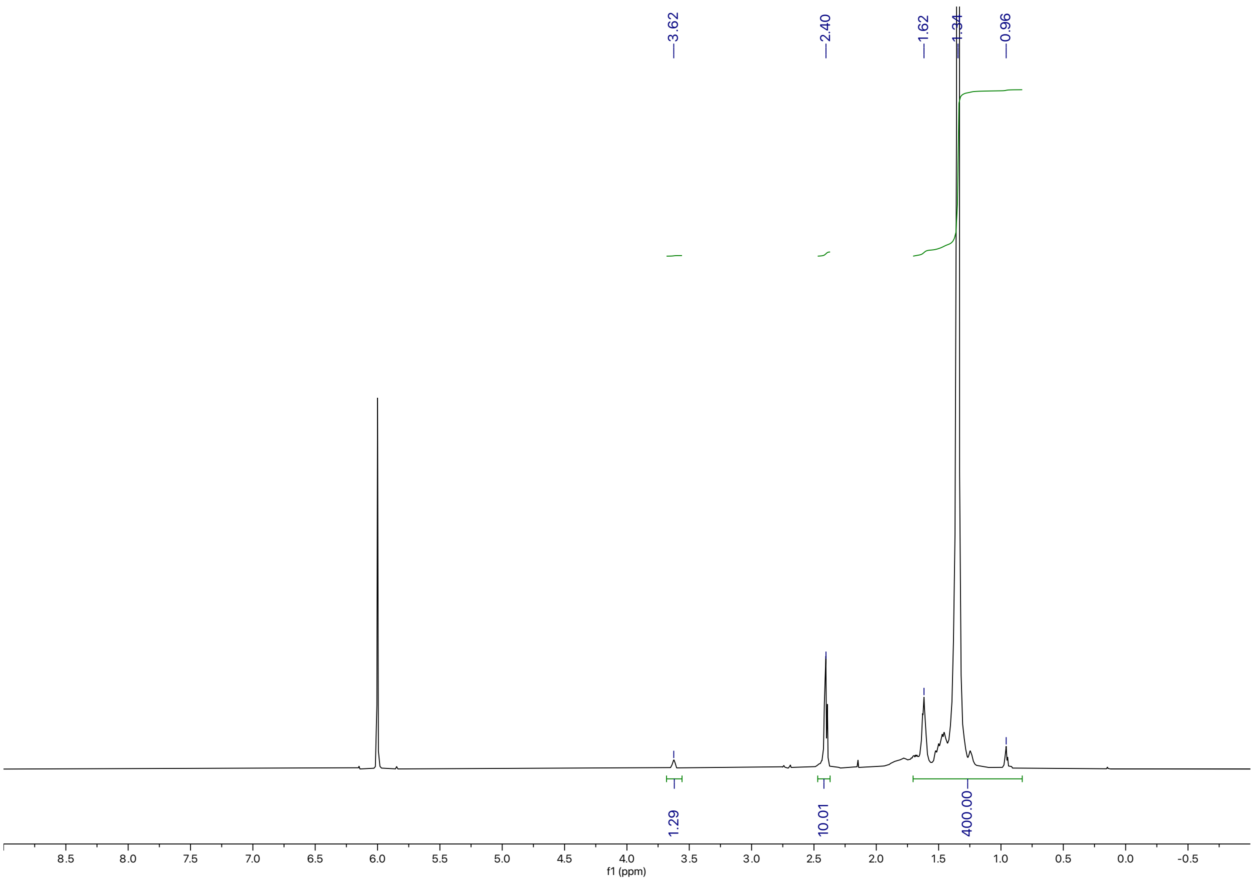

Figure S9. ^{1}H NMR spectrum of *oxo*-HDPE.

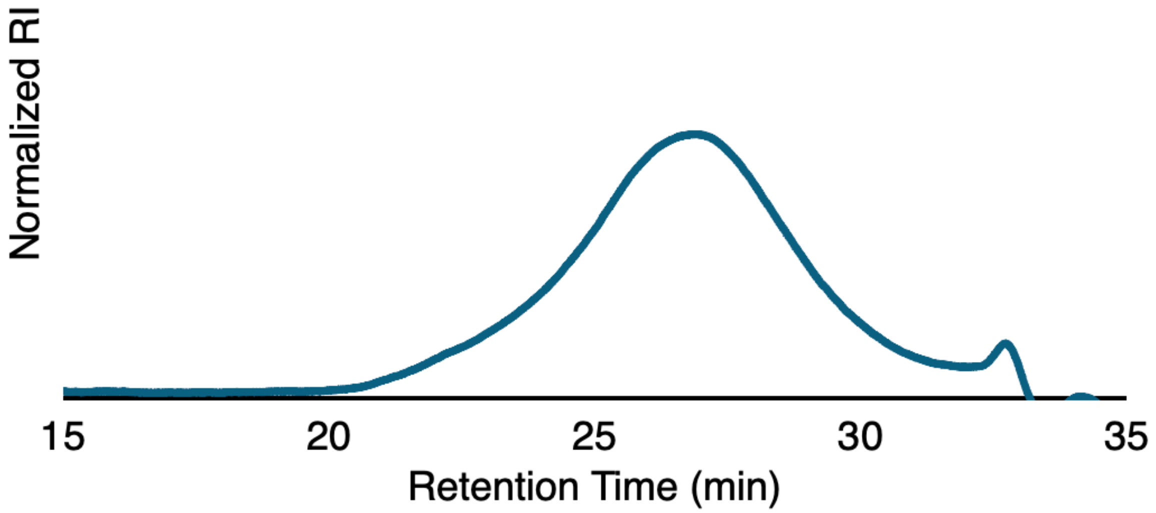

Figure S10. Size exclusion chromatogram of *oxo*-HDPE. M_n = 8.2 kDa, $Đ$ = 4.4. Molecular weight was determined relative to polyethylene standards.

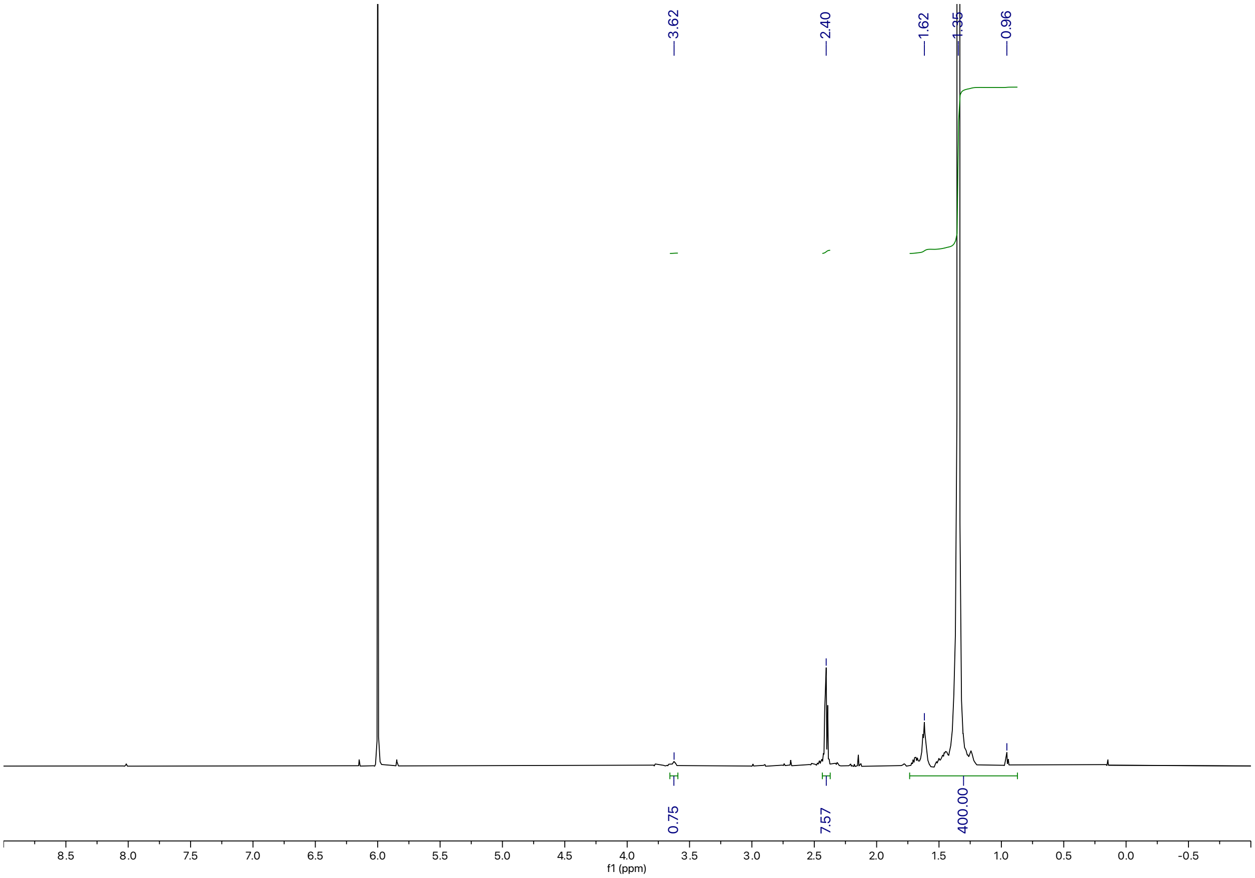

Figure S11. ^{1}H NMR spectrum of *oxo*-waste-HDPE (milk jug).

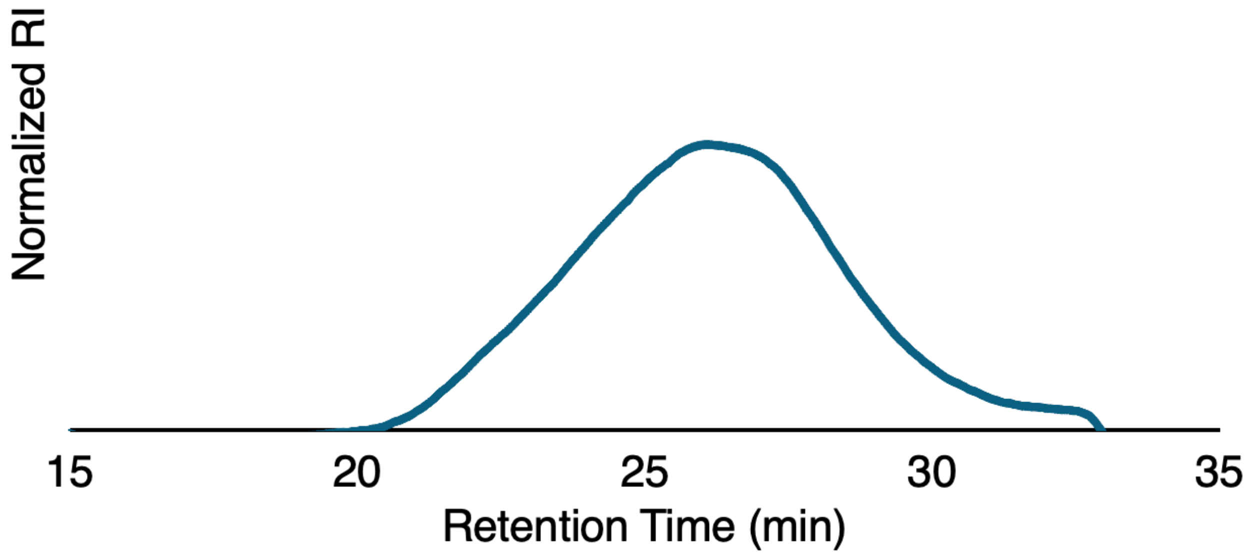

Figure S12. Size exclusion chromatogram of *oxo*-waste-HDPE (milk jug). M_n = 10.5 kDa, $Đ$ = 5.0. Molecular weight was determined relative to polyethylene standards.

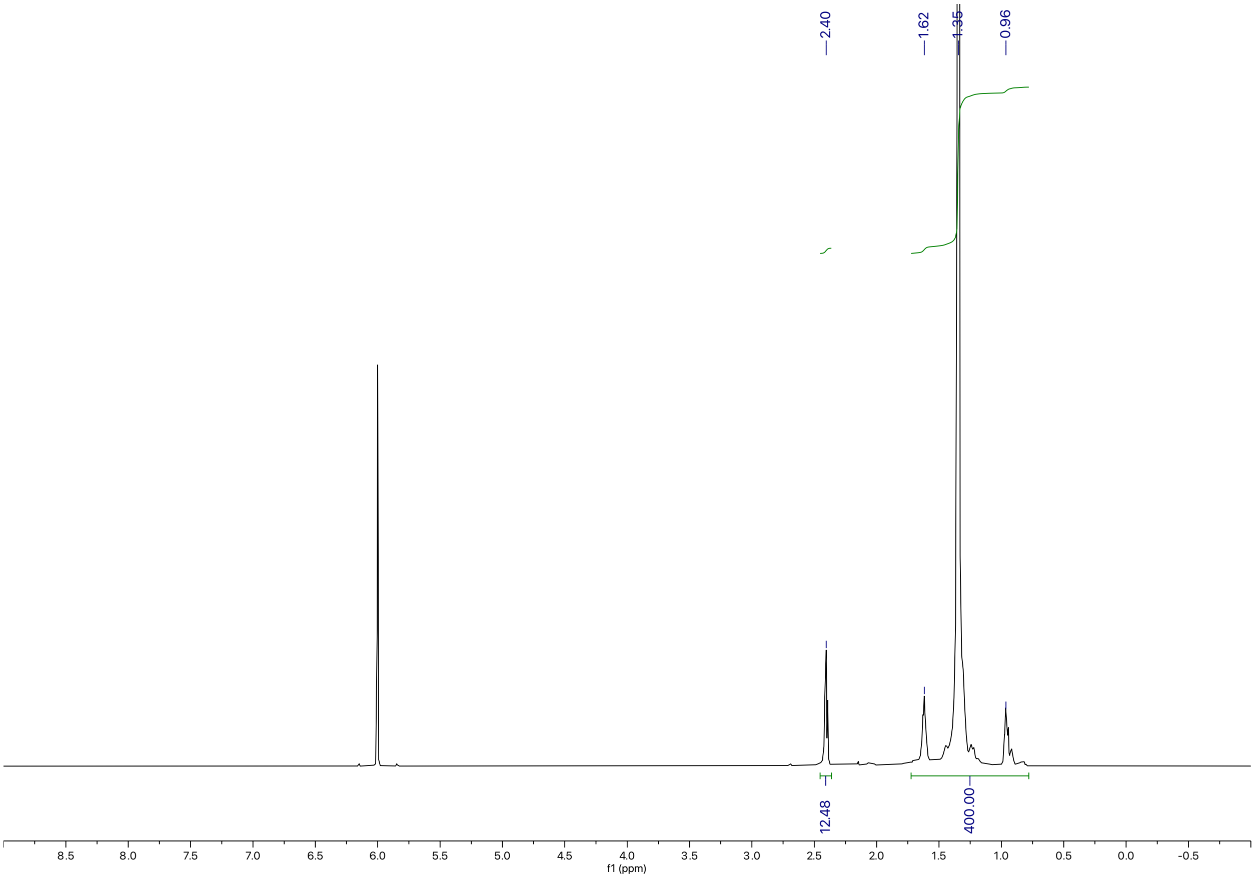

Figure S13. ^{1}H NMR spectrum of *keto*-LDPE.

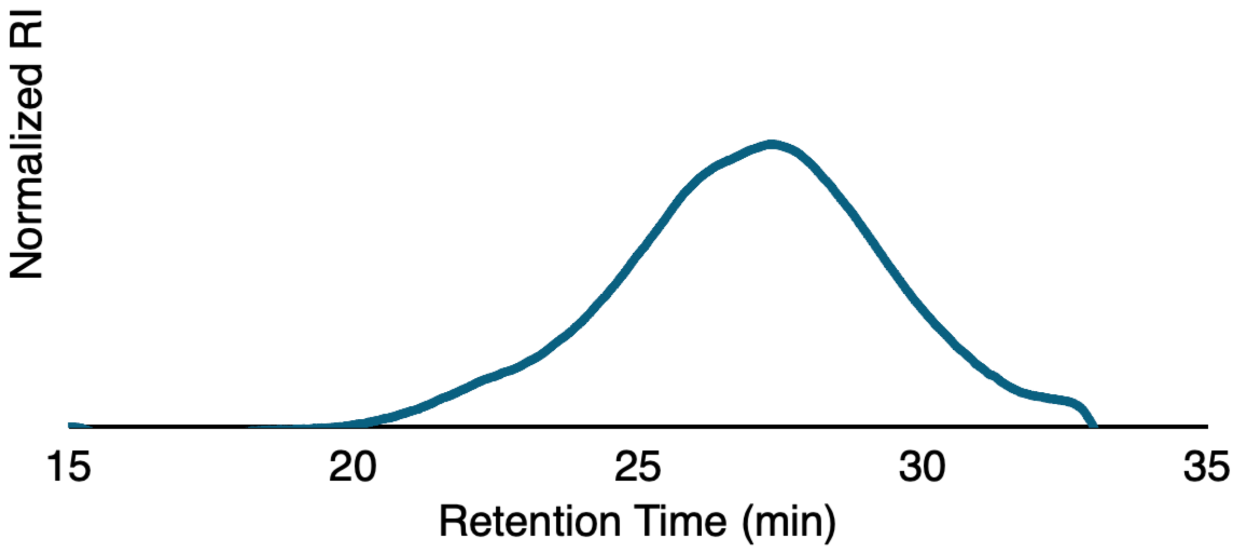

Figure S14. Size exclusion chromatogram of *keto*-LDPE. M_n = 6.3 kDa, $Đ$ = 5.8. Molecular weight was determined relative to polyethylene standards.

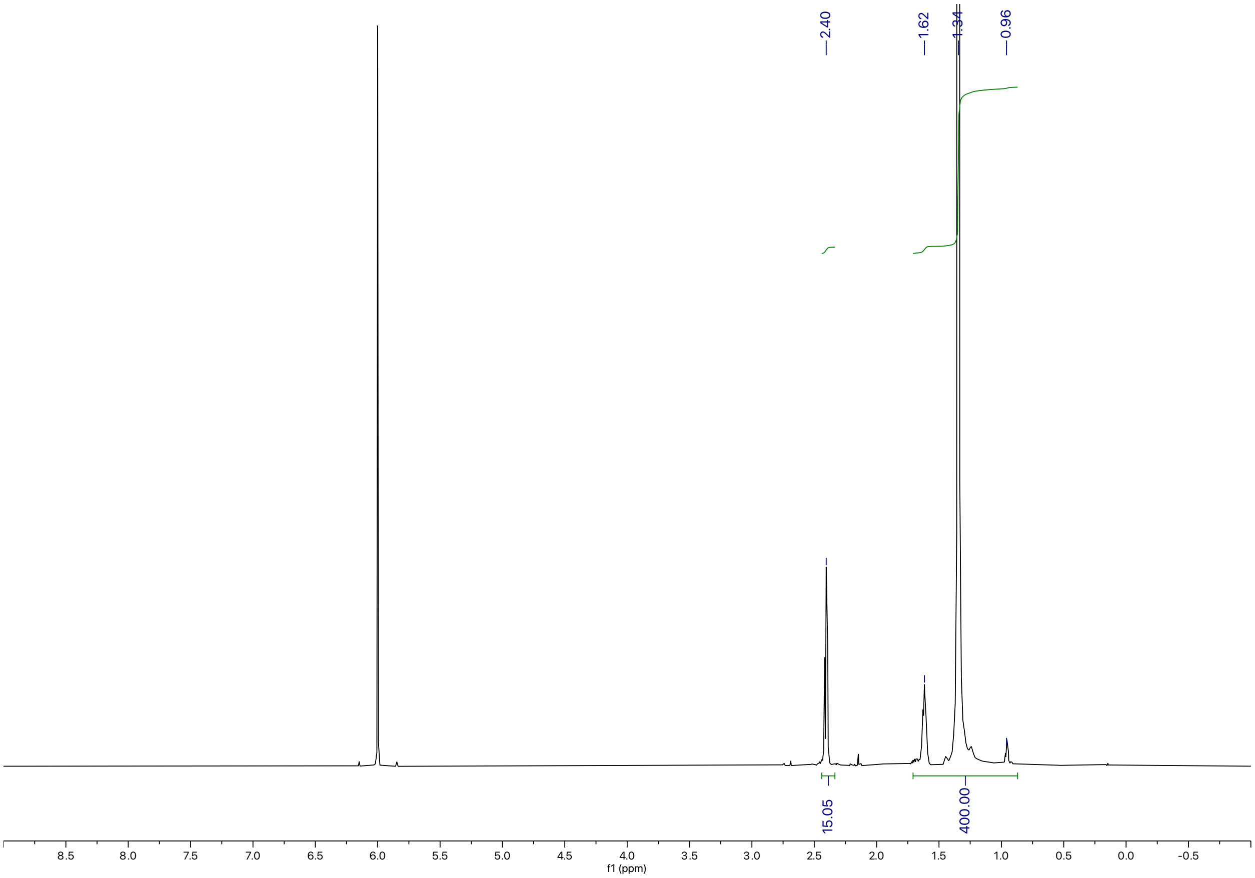

Figure S15. ^{1}H NMR spectrum of *keto*-HDPE.

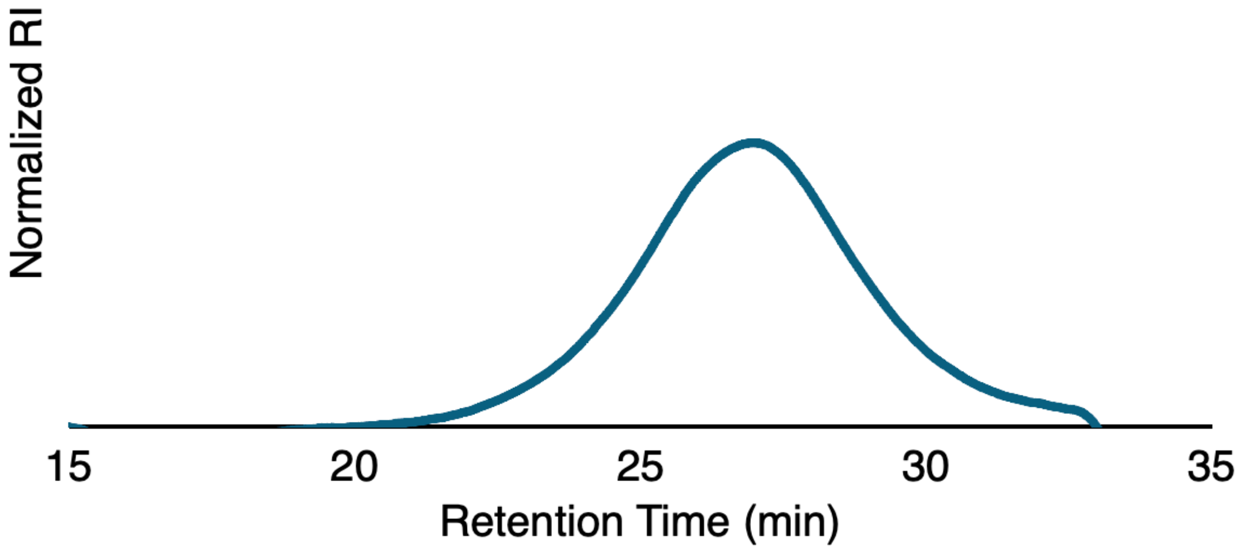

Figure S16. Size exclusion chromatogram of *keto*-HDPE. M_n = 6.9 kDa, $Đ$ = 3.7. Molecular weight was determined relative to polyethylene standards.

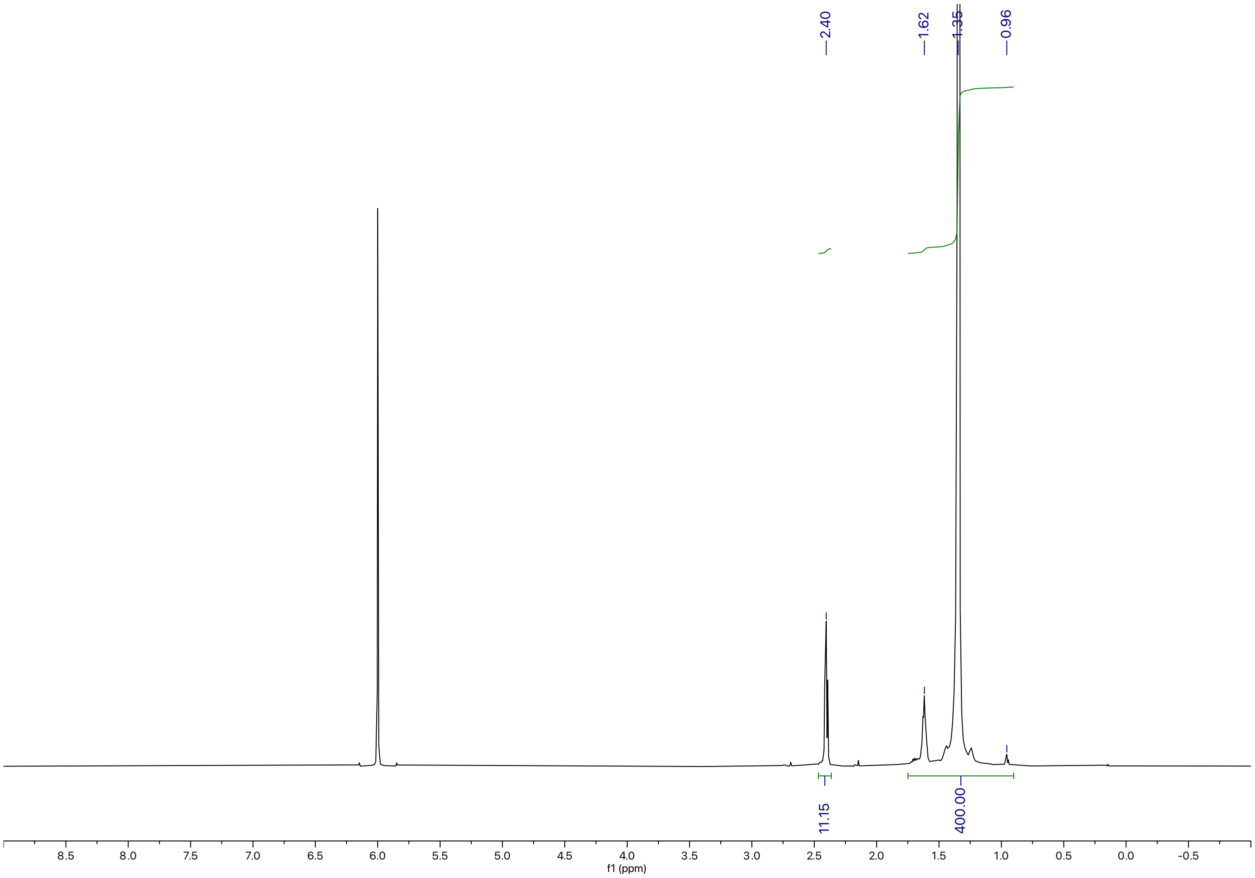

Figure S17. ^{1}H NMR spectrum of *keto*-waste-HDPE (milk jug).

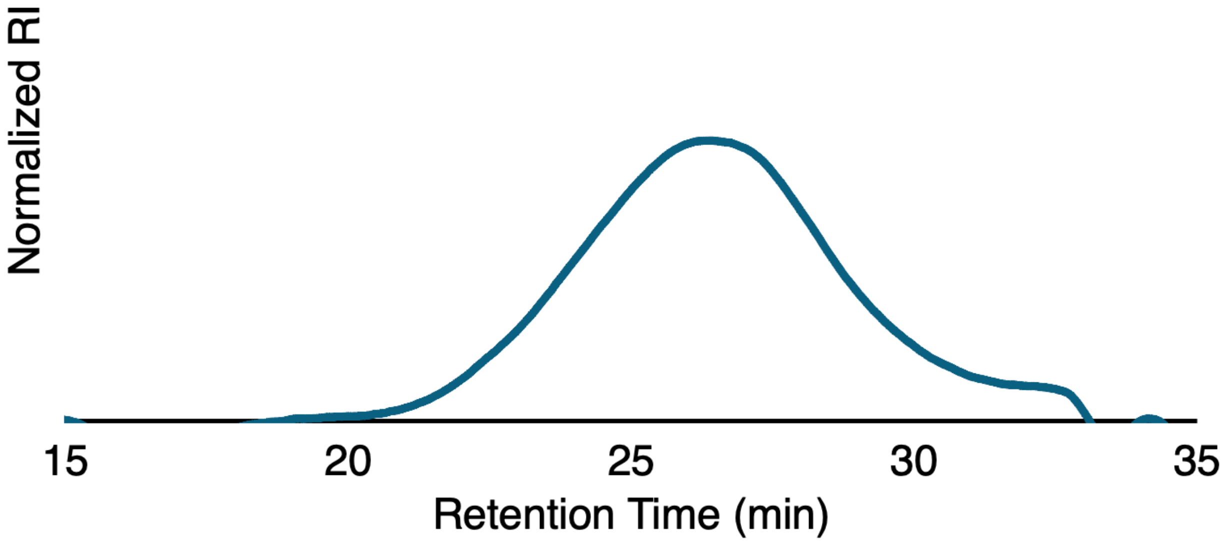

Figure S18. Size exclusion chromatogram of *keto*-waste HDPE (milk jug). M_n = 9.7 kDa, $Đ$ = 4.2. Molecular weight was determined relative to polyethylene standards.

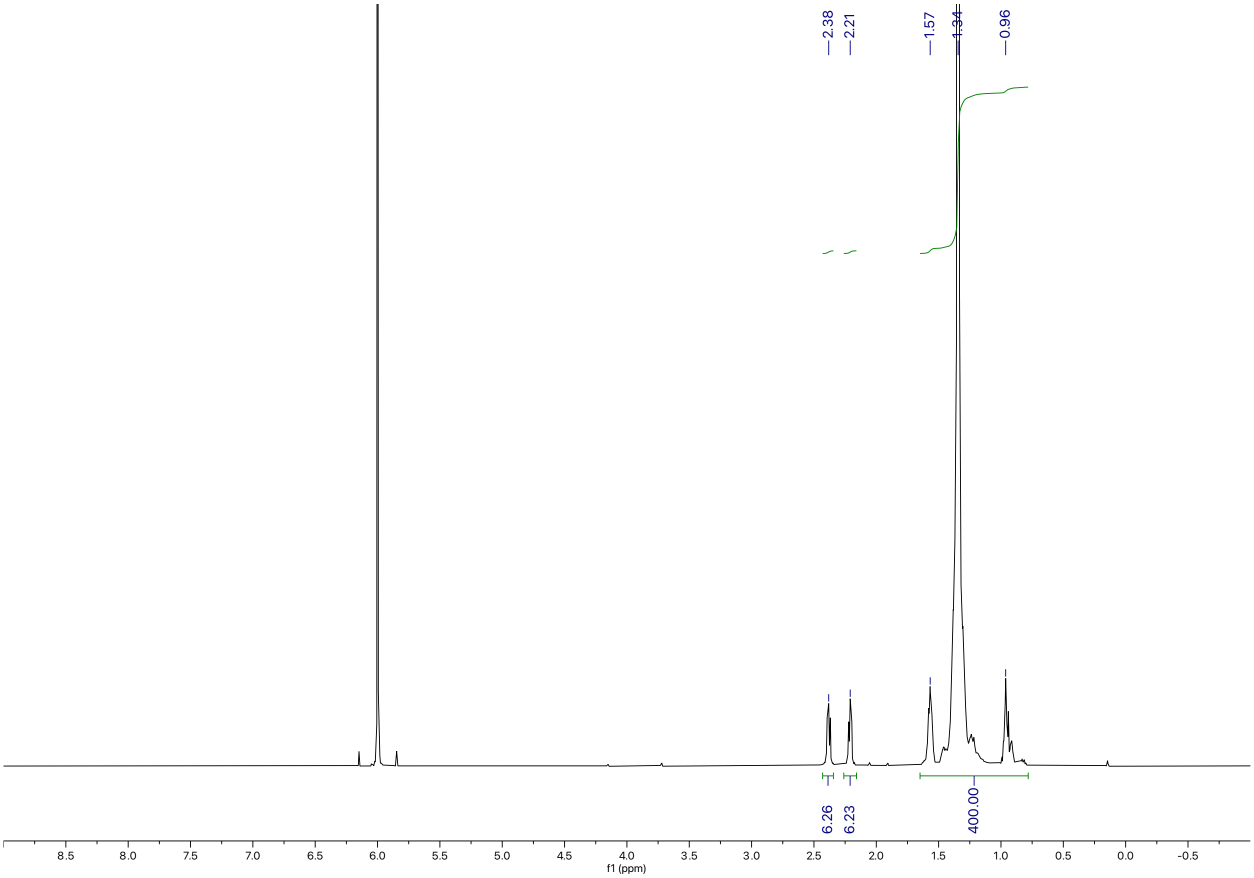

Figure S19. ^{1}H NMR spectrum of *oxime*-LDPE.

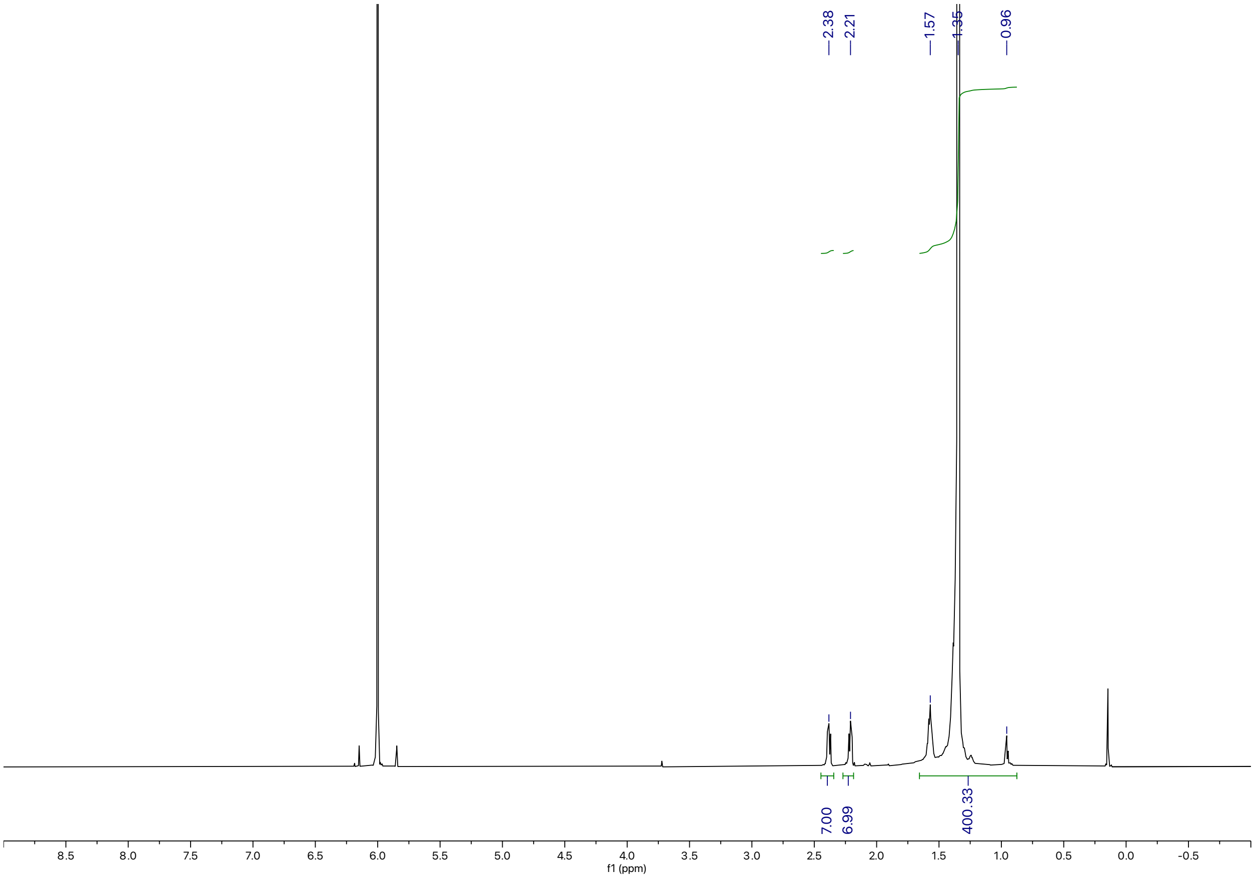

Figure S20. ^{1}H NMR spectrum of *oxime*-HDPE.

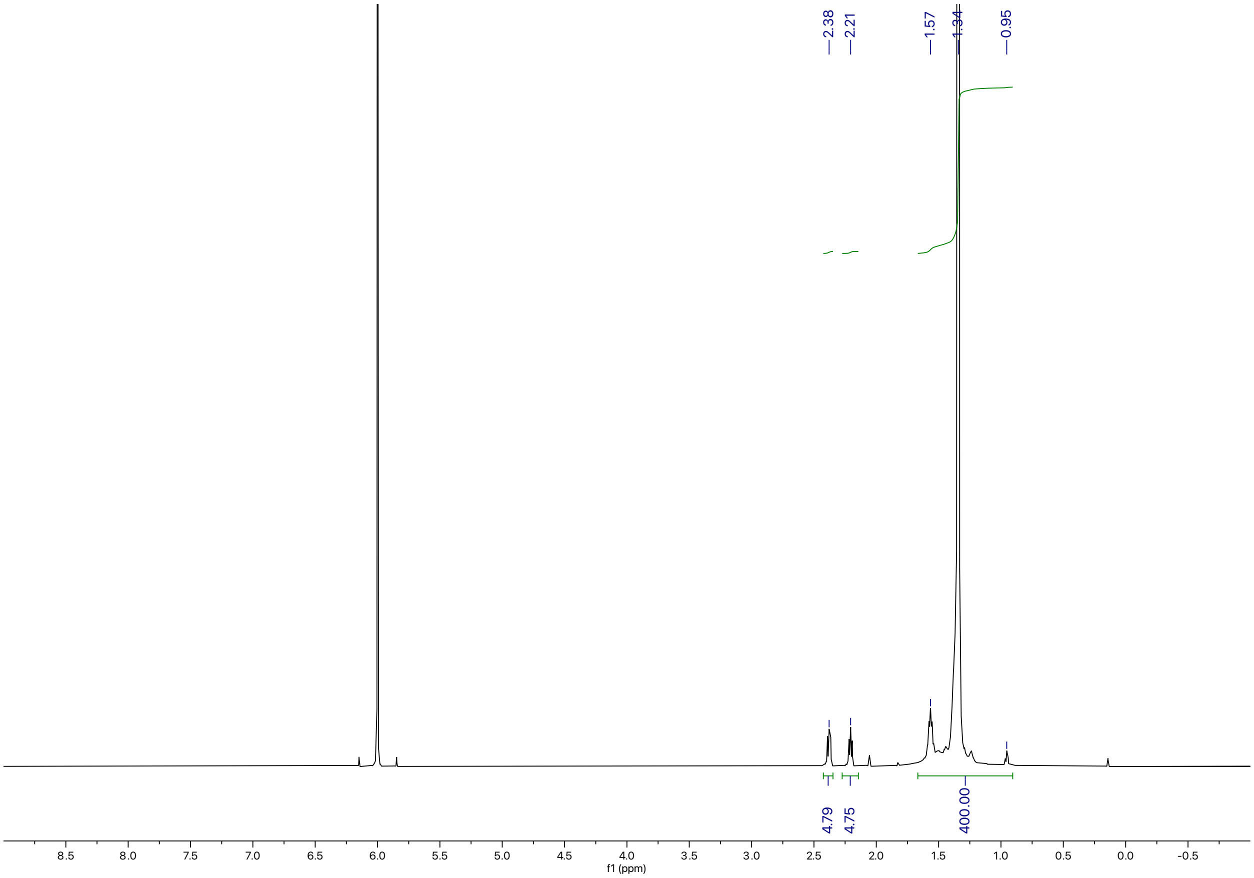

Figure S21. ^{1}H NMR spectrum of *oxime*-waste-HDPE (milk jug).

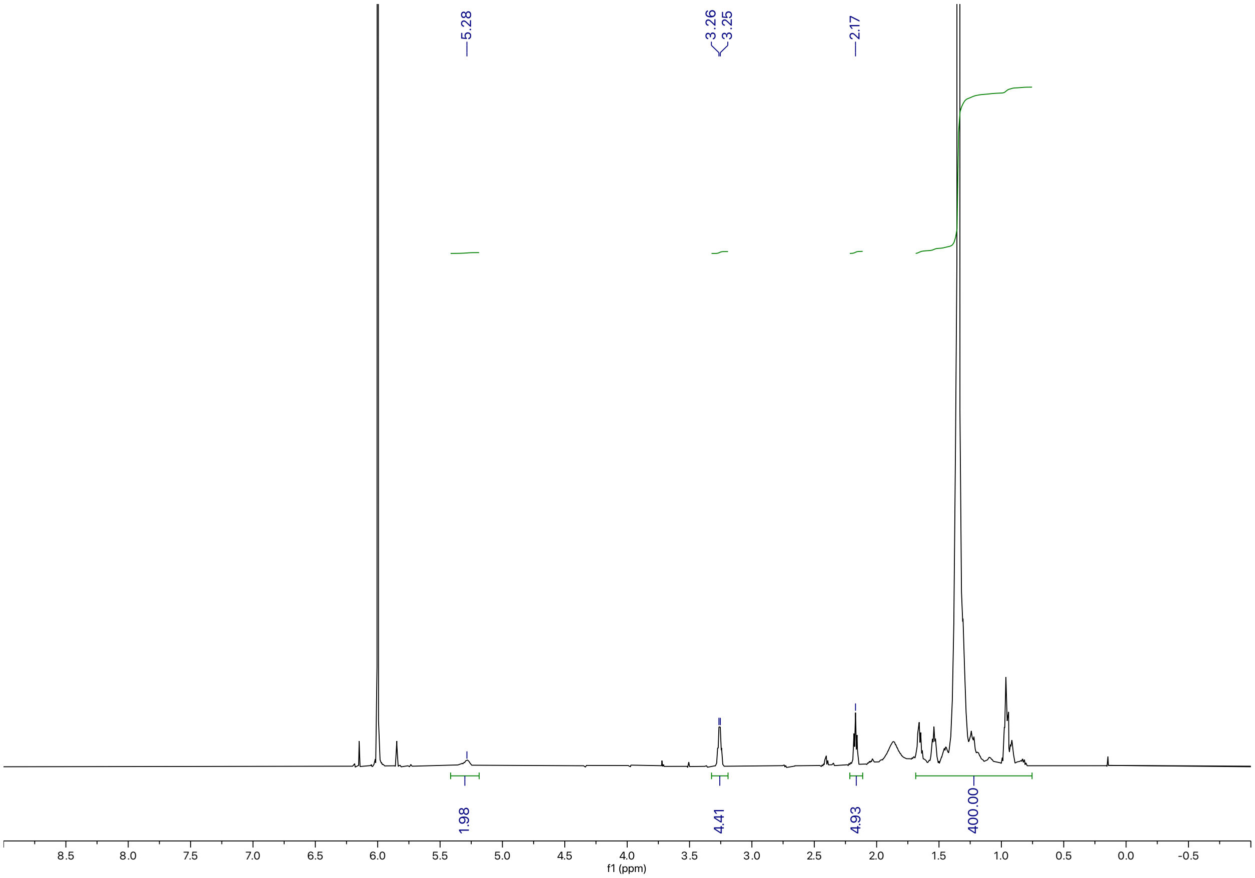

Figure S22. ^{1}H NMR spectrum of *amide*-LDPE **2a**.

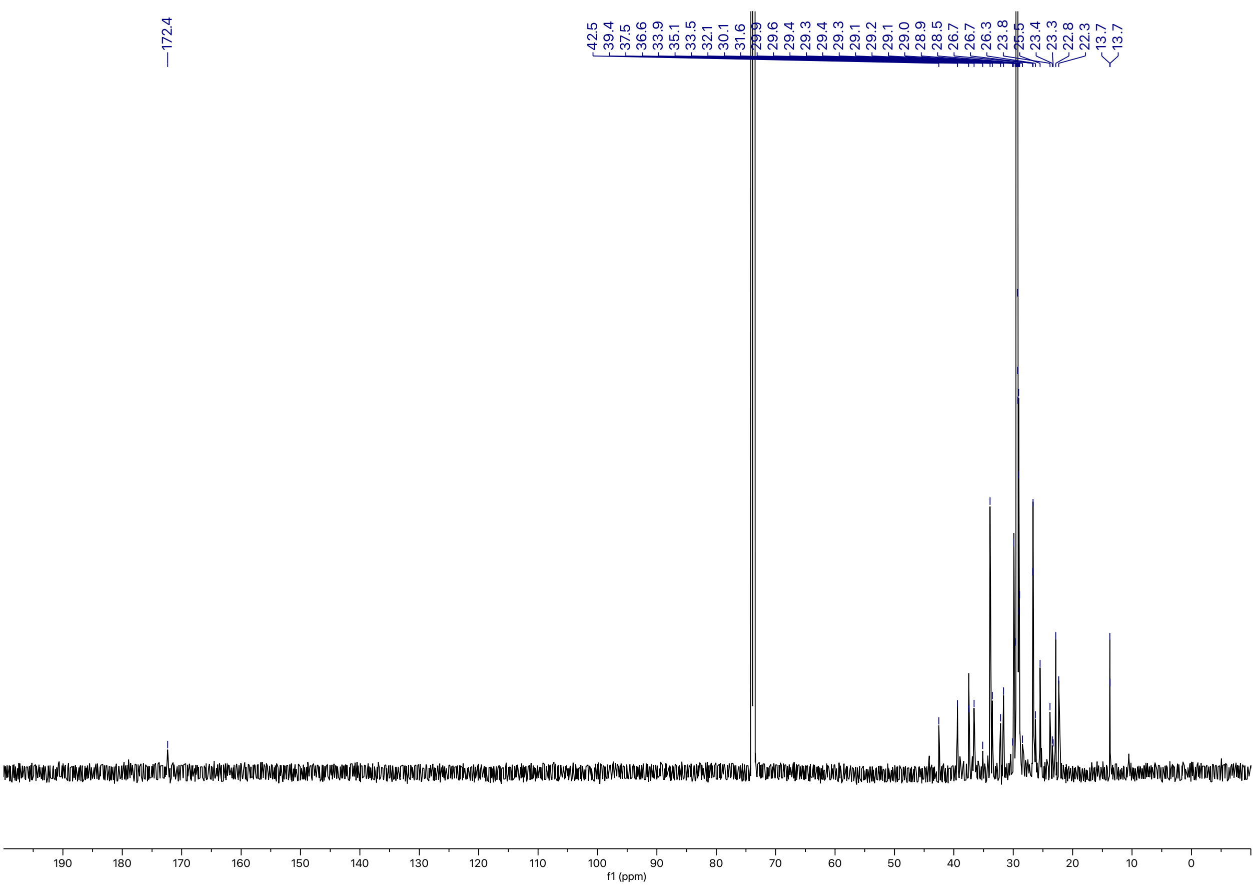

Figure S23. ^{13}C NMR spectrum of *amide*-LDPE **2a**.

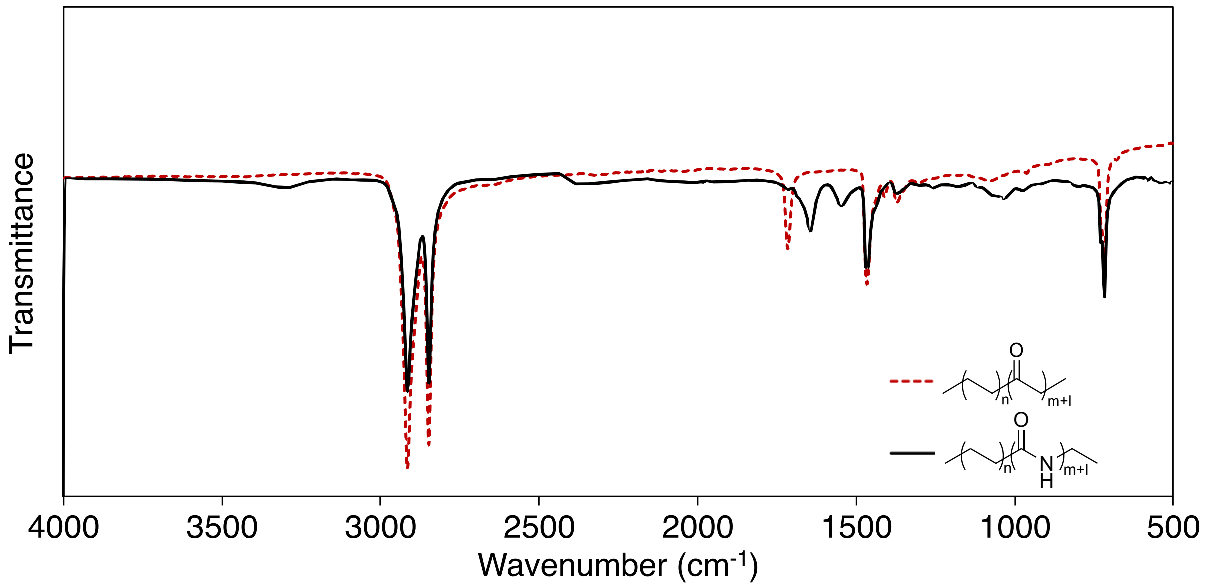

Figure S24. Overlay of the FTIR spectrum of *amide*-LDPE **2a**, major peaks ν (cm^{-1}): 3305, 2915, 2848, 1645, 1548, 1464, 1370, 1261, 1035, 729, 718, and the FTIR spectrum of *keto*-LDPE, major peaks ν (cm^{-1}): 2916, 2849, 1717, 1467, 1412, 1373, 1084, 719.

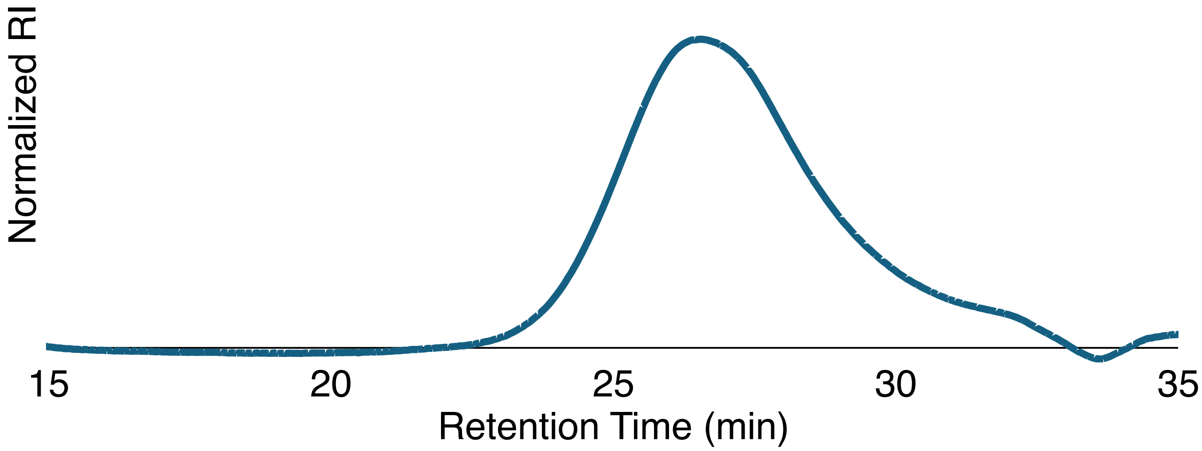

Figure S25. Size exclusion chromatogram of *amide*-LDPE **2a**. M_n = 8.1 kDa, $Đ$ = 2.4. Molecular weight was determined relative to polyethylene standards.

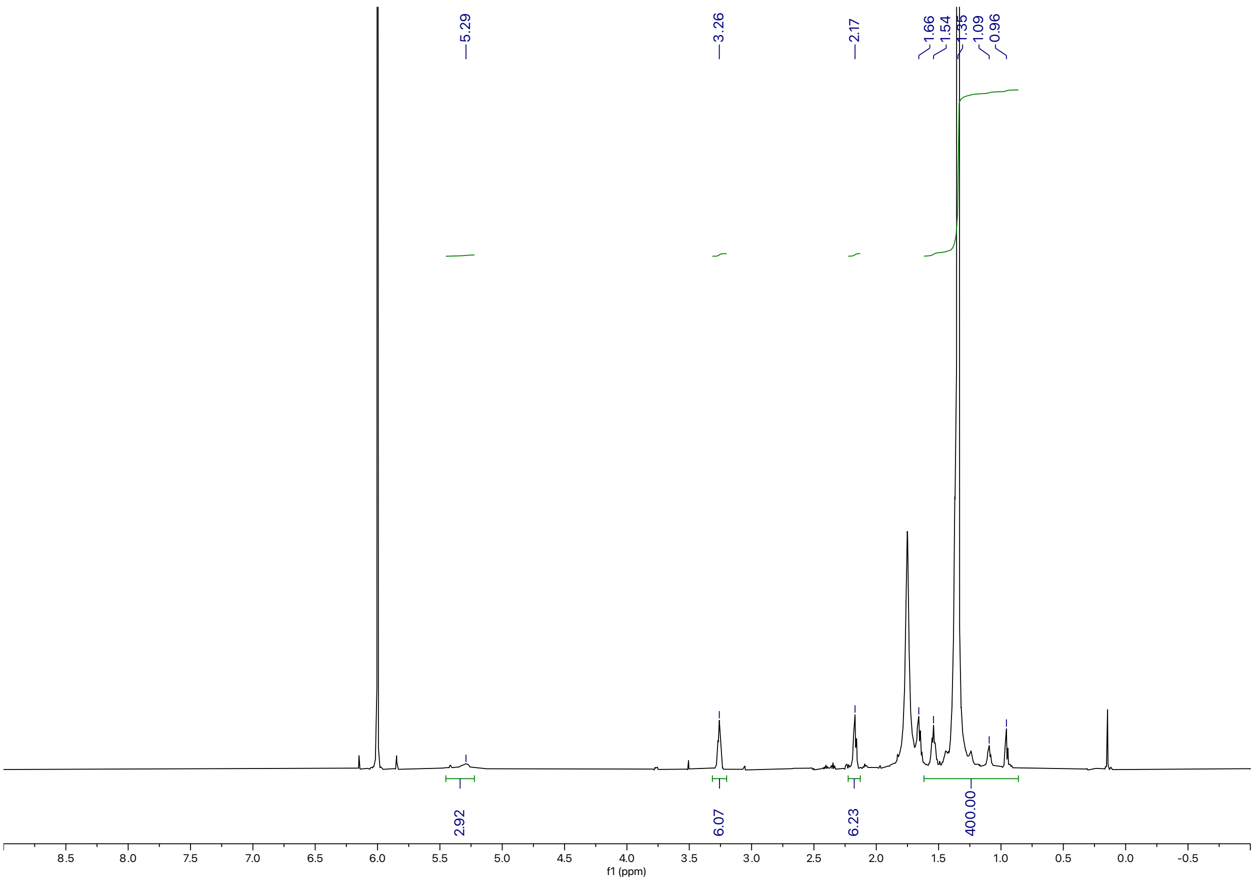

Figure S26. ^{1}H NMR spectrum of *amide*-HDPE **2b**.

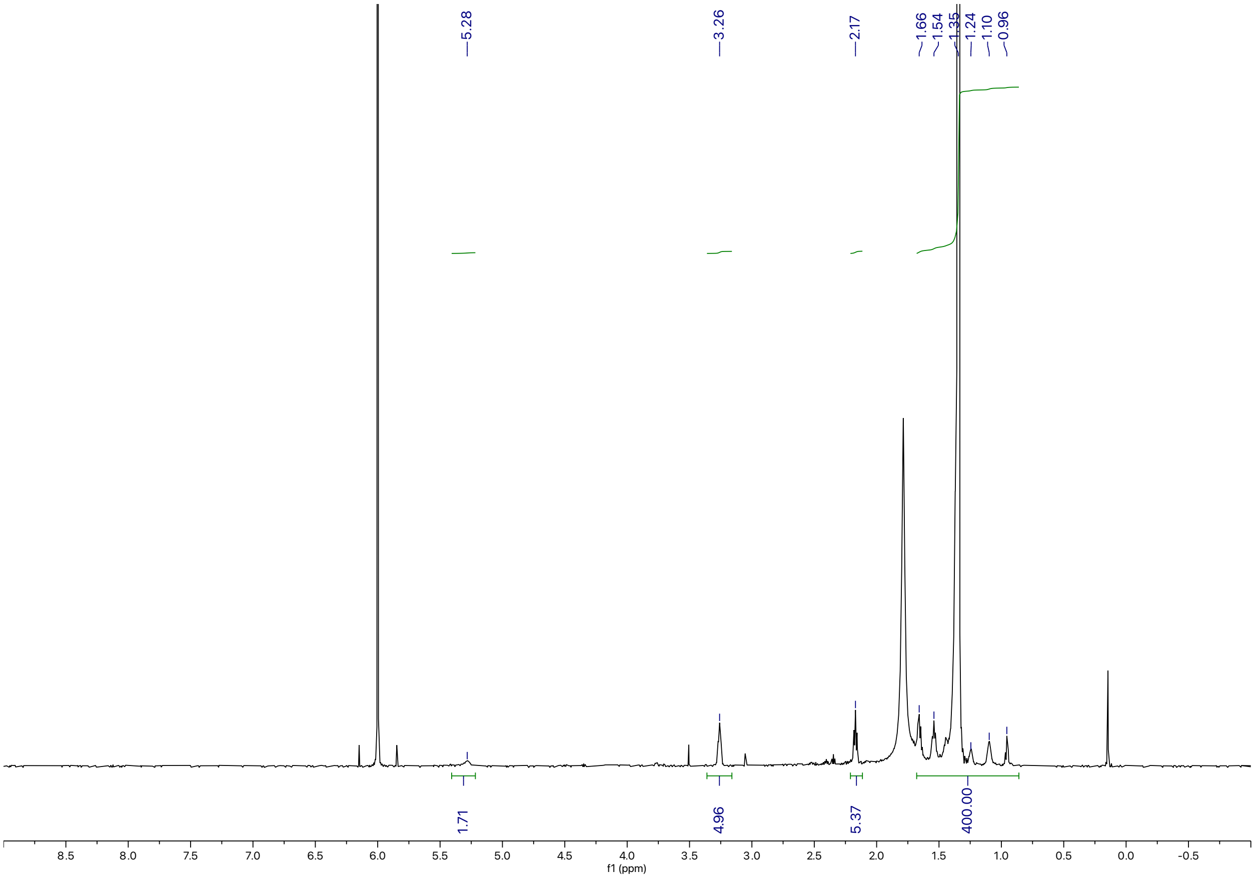

Figure S27. ^{1}H NMR spectrum of *amide*-*waste*-HDPE (milk jug) **2c**.

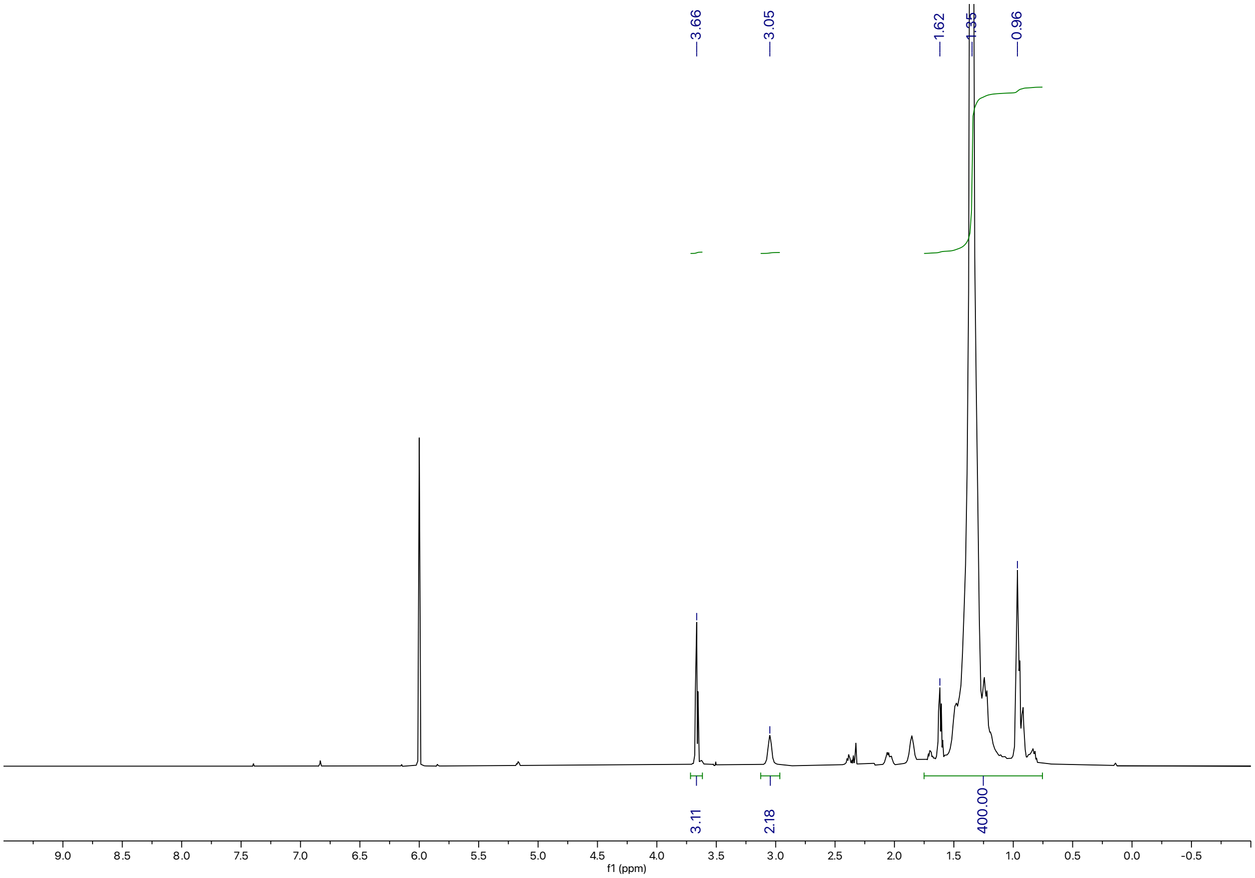

Figure S28. ^{1}H NMR spectrum of the hydrogenolysis of *amide*-LDPE **2a**.

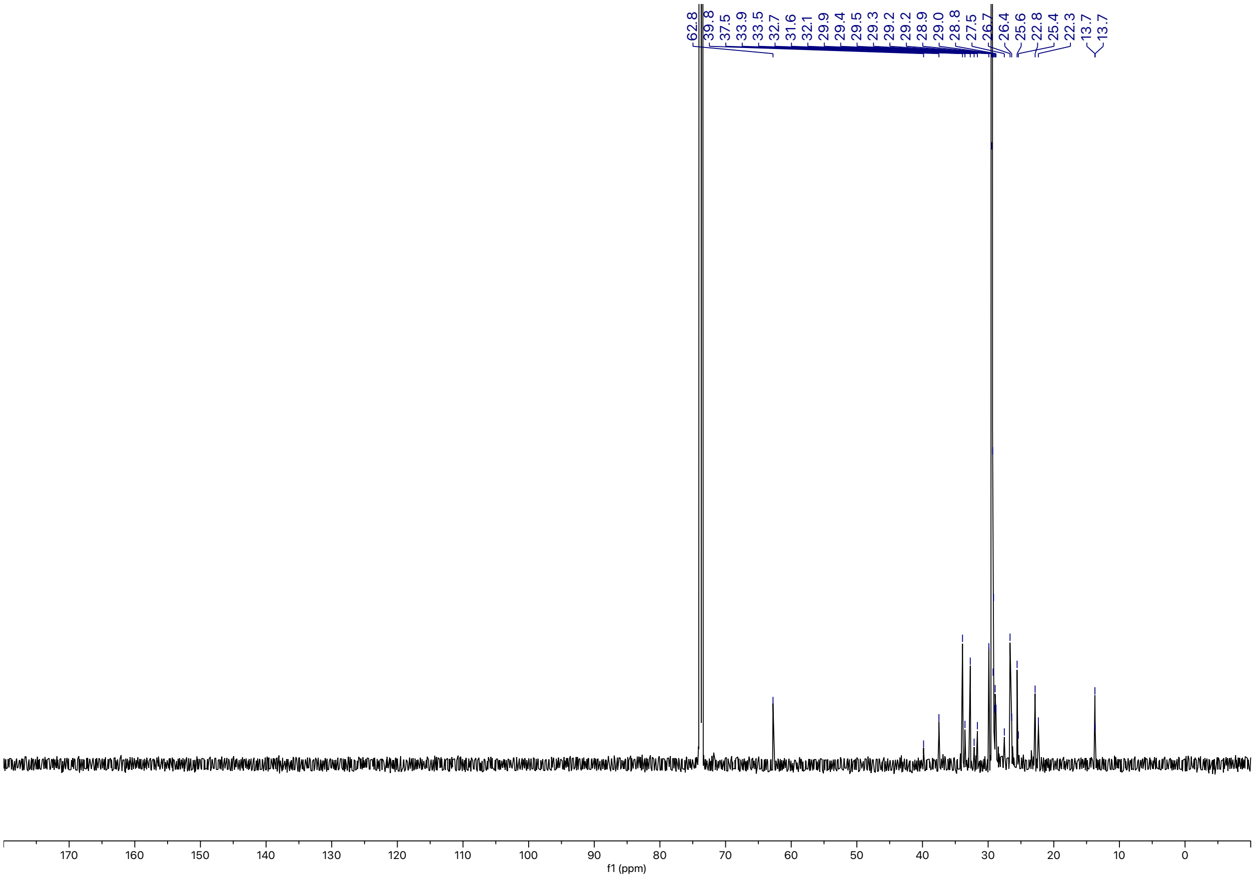

Figure S29. ^{13}C NMR spectrum of the hydrogenolysis of *amide*-LDPE **2a**.

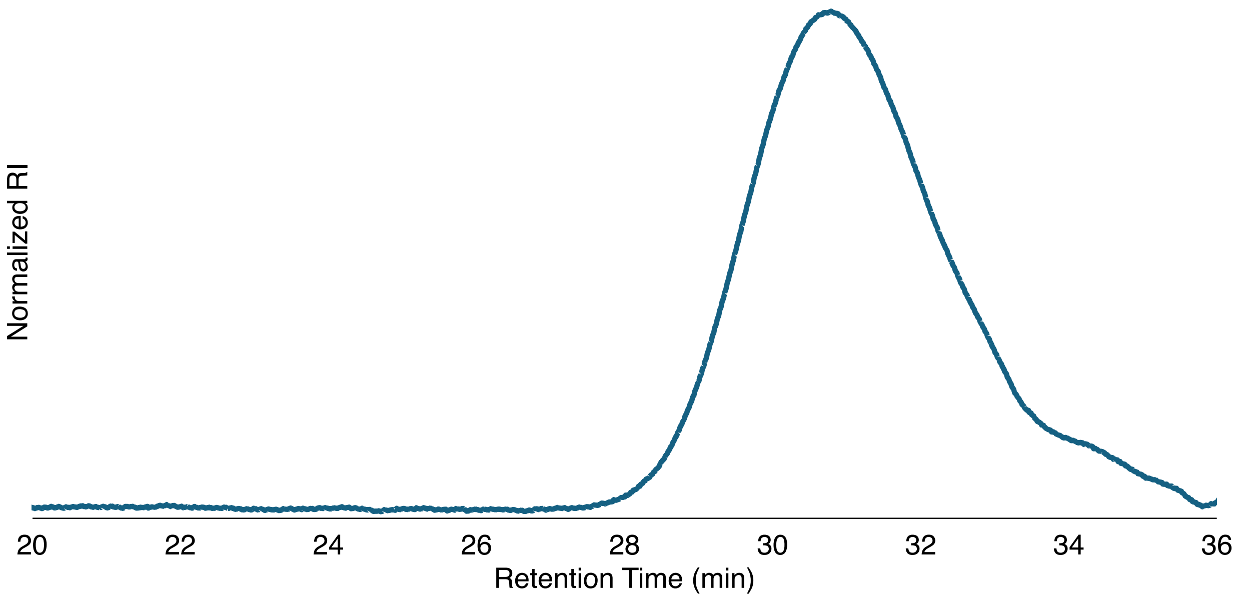

Figure S30. Size exclusion chromatogram of the hydrogenolysis product of *amide*-LDPE **2a**. M_n = 803 Da, $Đ$ = 1.5. Molecular weight was determined relative to polyethylene standards.

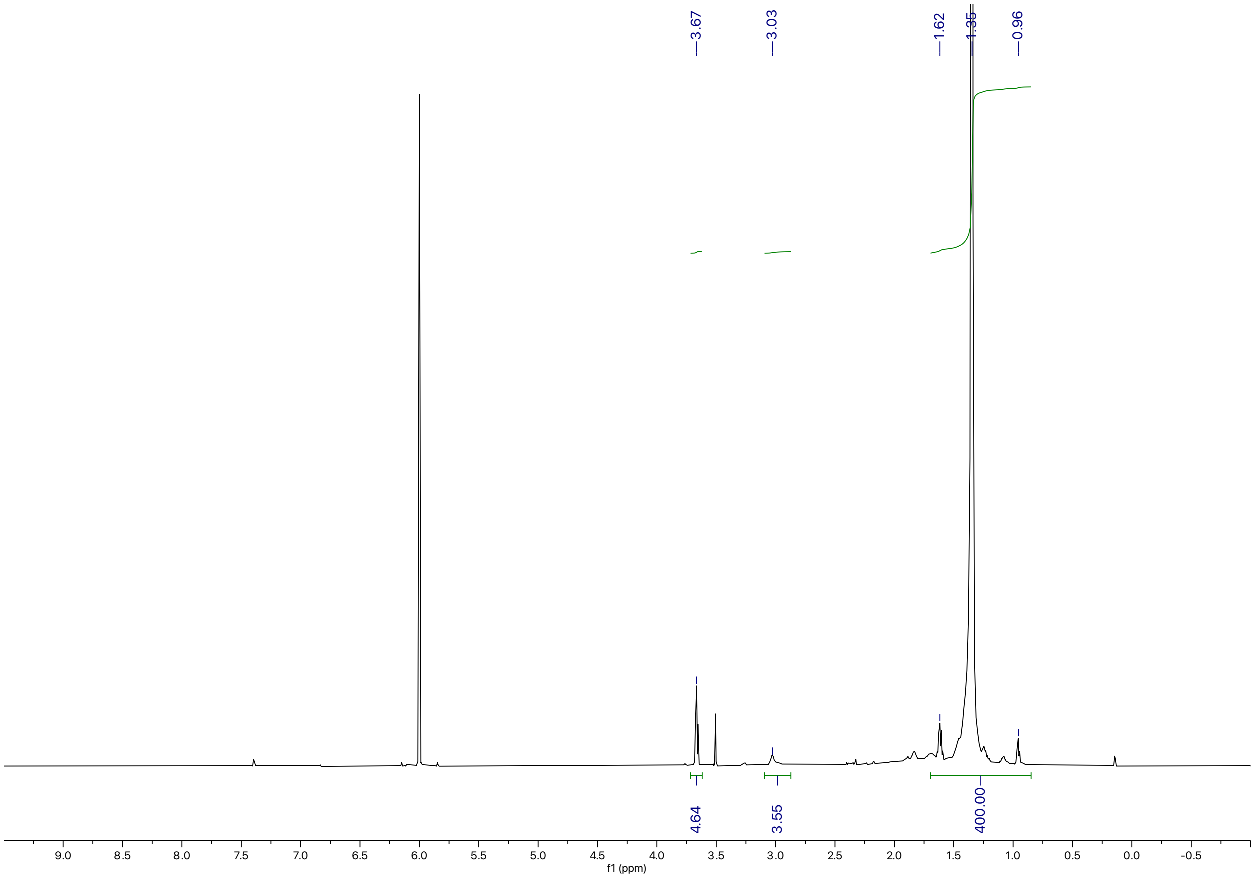

Figure S31. ^{1}H NMR spectrum of the hydrogenolysis of *amide*-HDPE **2b**.

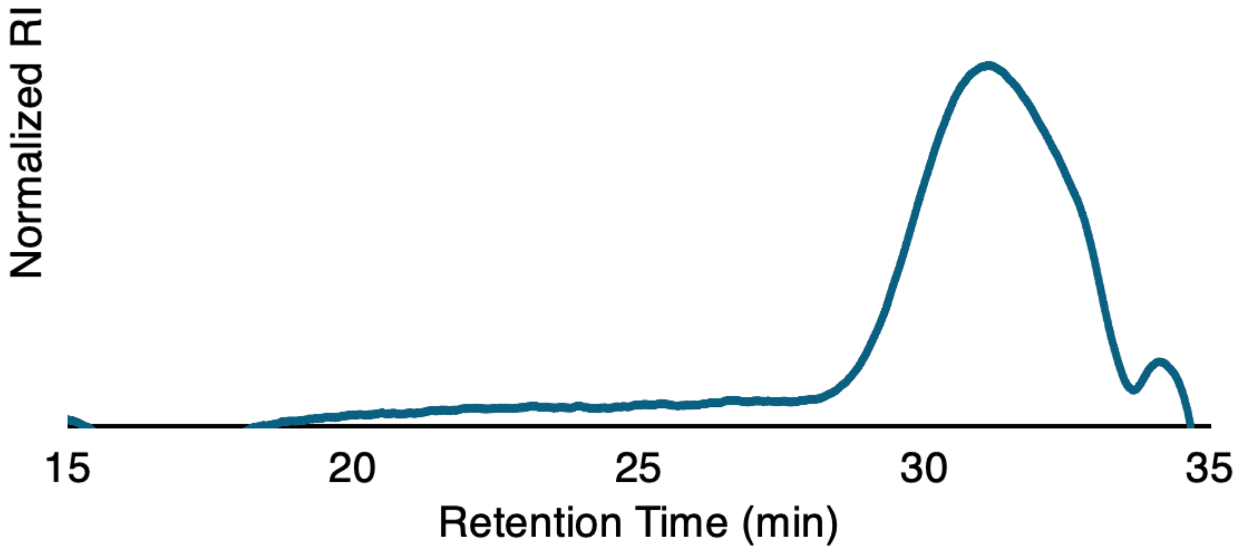

Figure S32. Size exclusion chromatogram of the hydrogenolysis product of *amide*-HDPE **2b**. M_n = 661 Da, $Đ$ = 1.5. Molecular weight was determined relative to polyethylene standards.

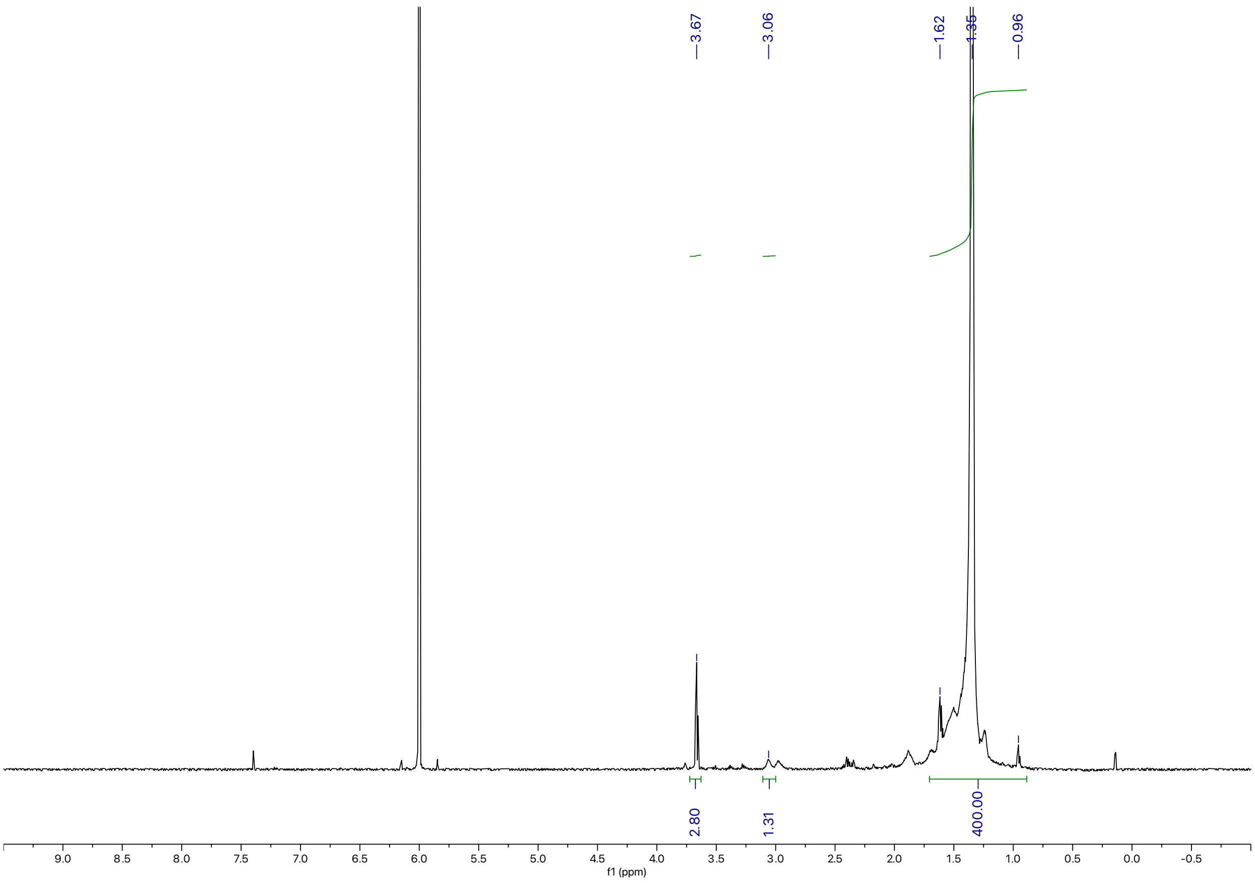

Figure S33. ^{1}H NMR spectrum of the hydrogenolysis of *amide*-waste-HDPE (milk jug) **2c**.

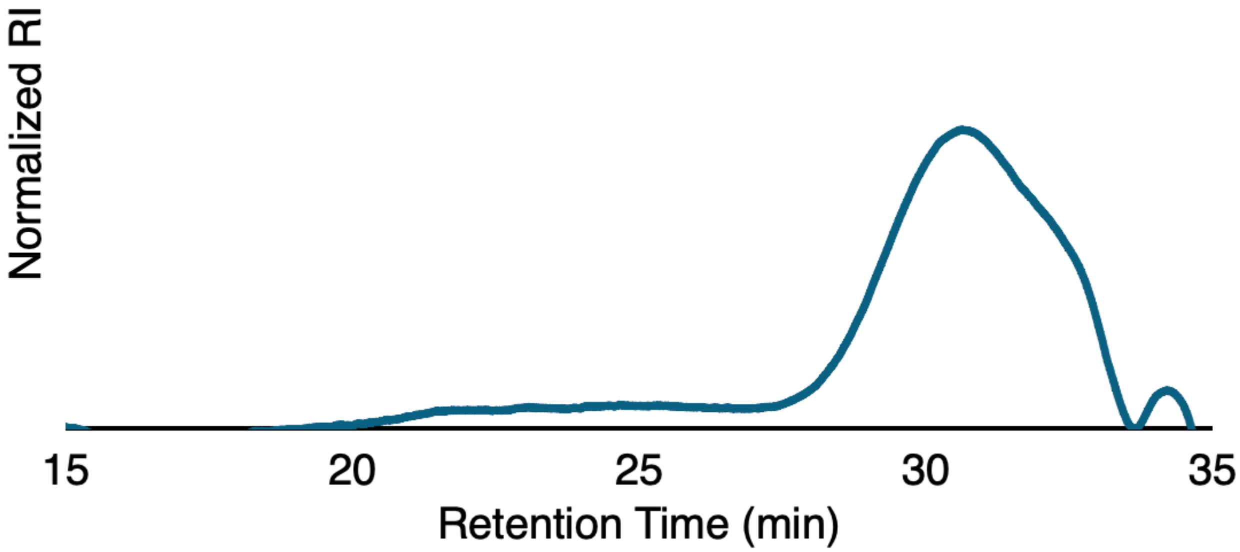

Figure S34. Size exclusion chromatogram of the hydrogenolysis product of *amide*-waste-HDPE (milk jug) **2c**. M_n = 760 Da, $Đ$ = 1.8. Molecular weight was determined relative to polyethylene standards.

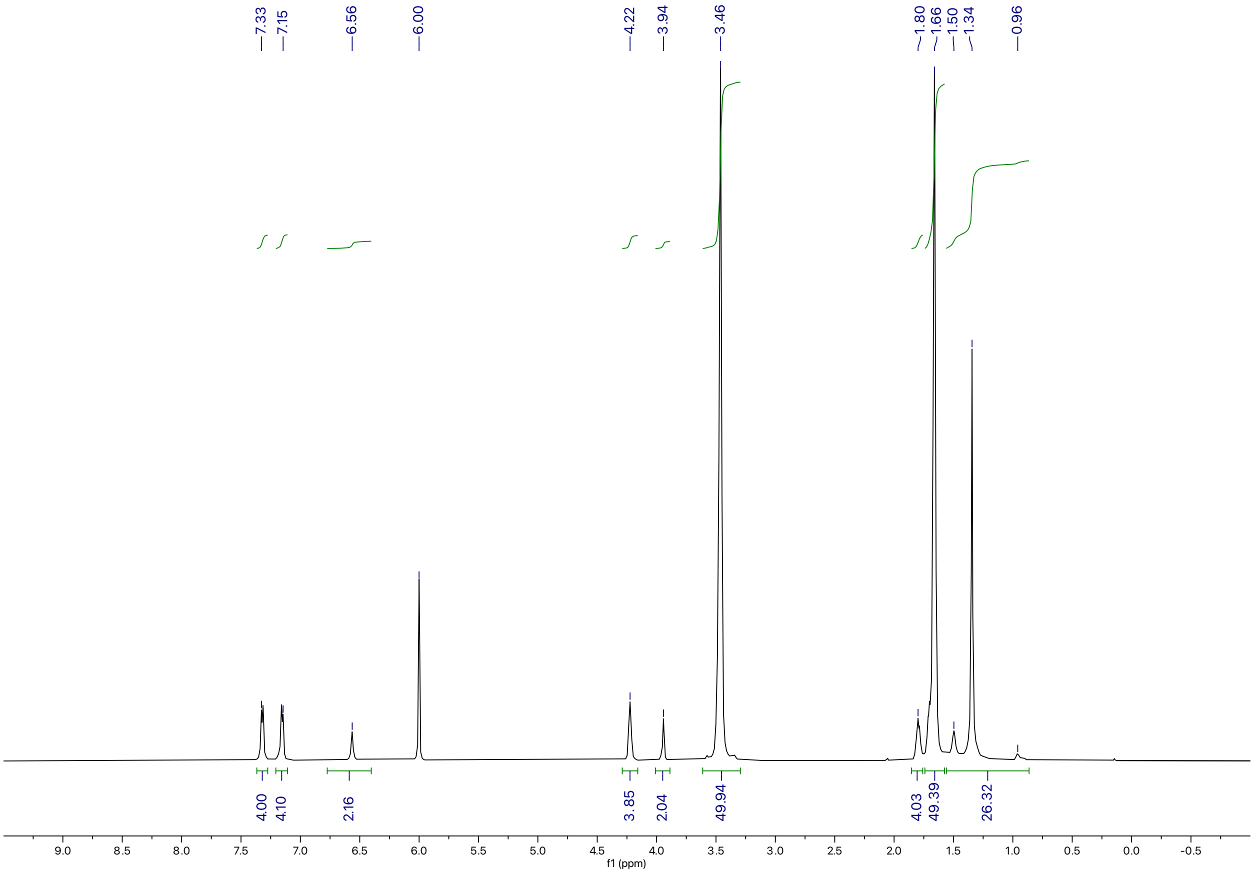

Figure S35. ^{1}H NMR spectrum of PUU **4**.

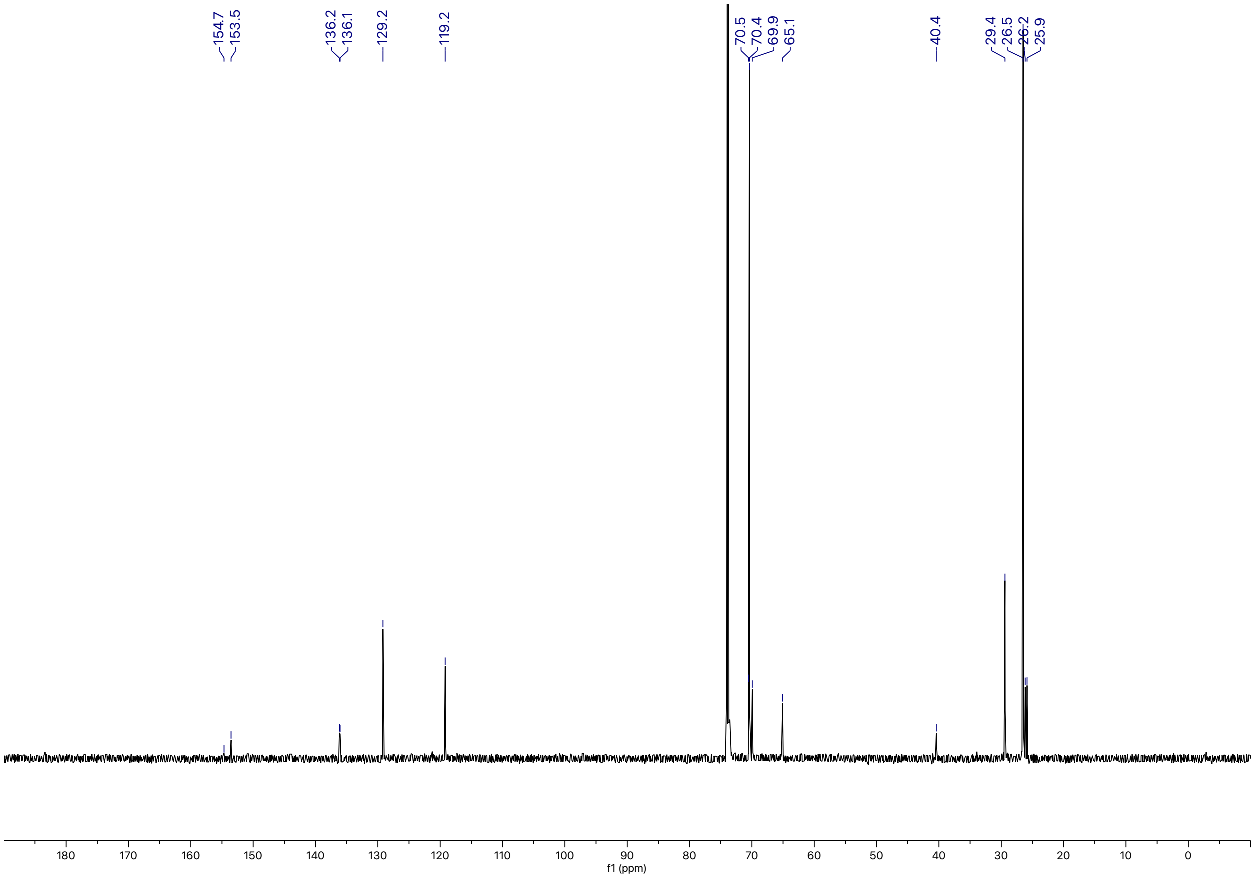

Figure S36. ^{13}C NMR spectrum of PUU **4**.

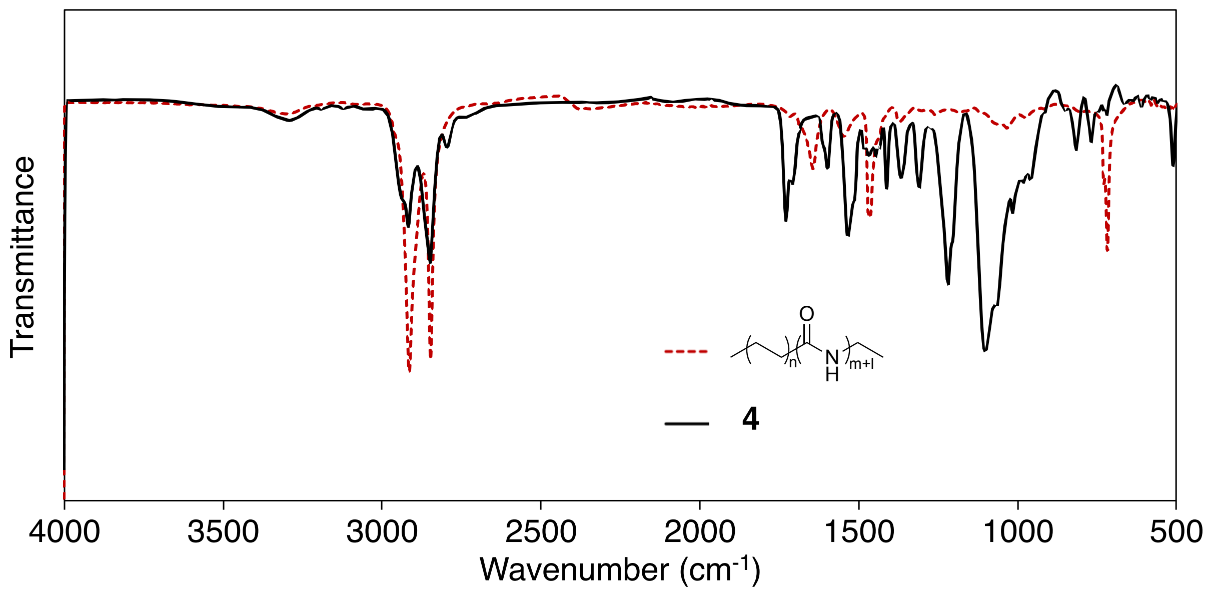

Figure S37. Overlay of the FTIR spectrum of PUU **4**, major peaks ν (cm^{-1}): 3292, 2918, 2849, 2796, 1730, 1710, 1599, 1534, 1467, 1447, 1413, 1367, 1310, 1220, 1103, 1017, 982, 960, 816, 767, 720, 611, 511, and the FTIR spectrum of *amide*-LDPE **2a**, major peaks ν (cm^{-1}): 3305, 2915, 2848, 1645, 1548, 1464, 1370, 1261, 1035, 729, 718.

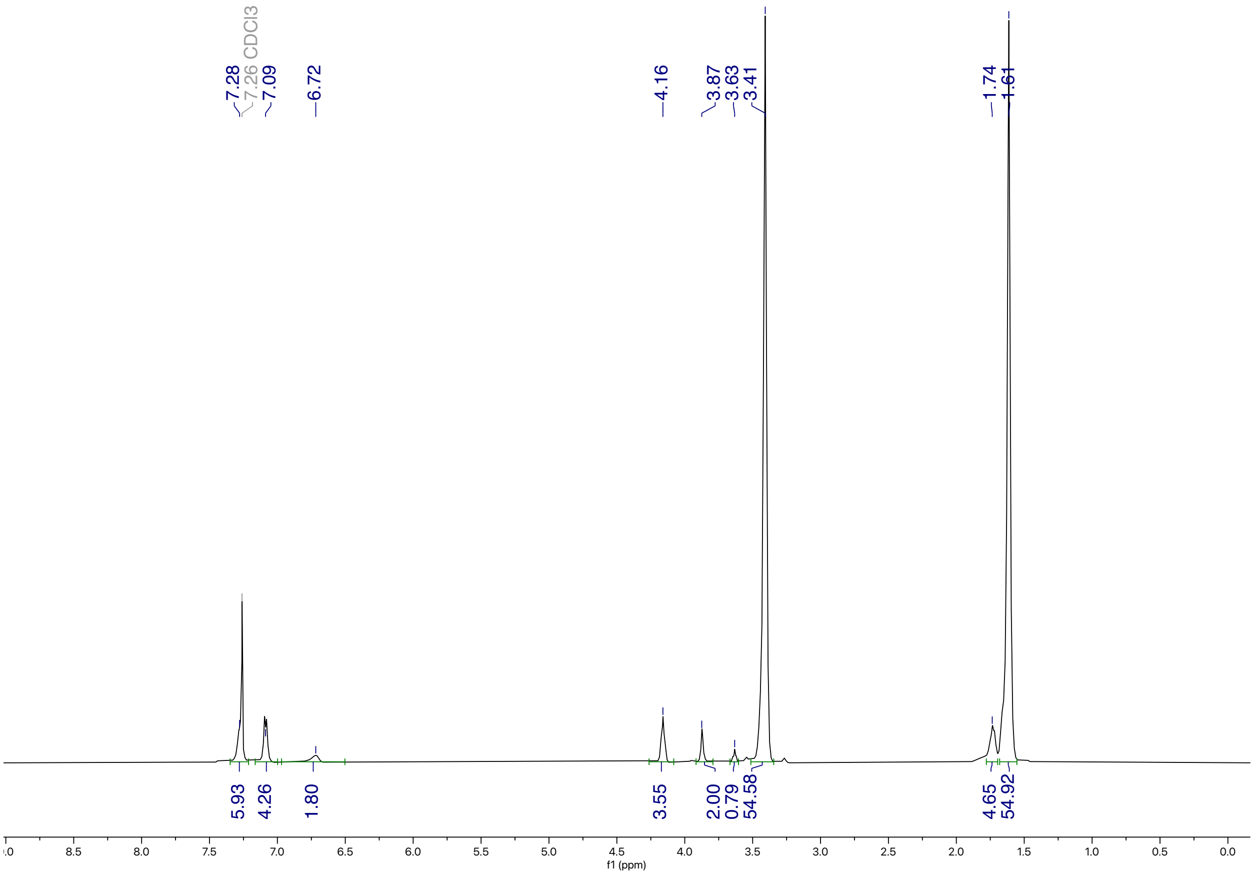

Figure S38. ^{1}H NMR spectrum of PU **4’**.

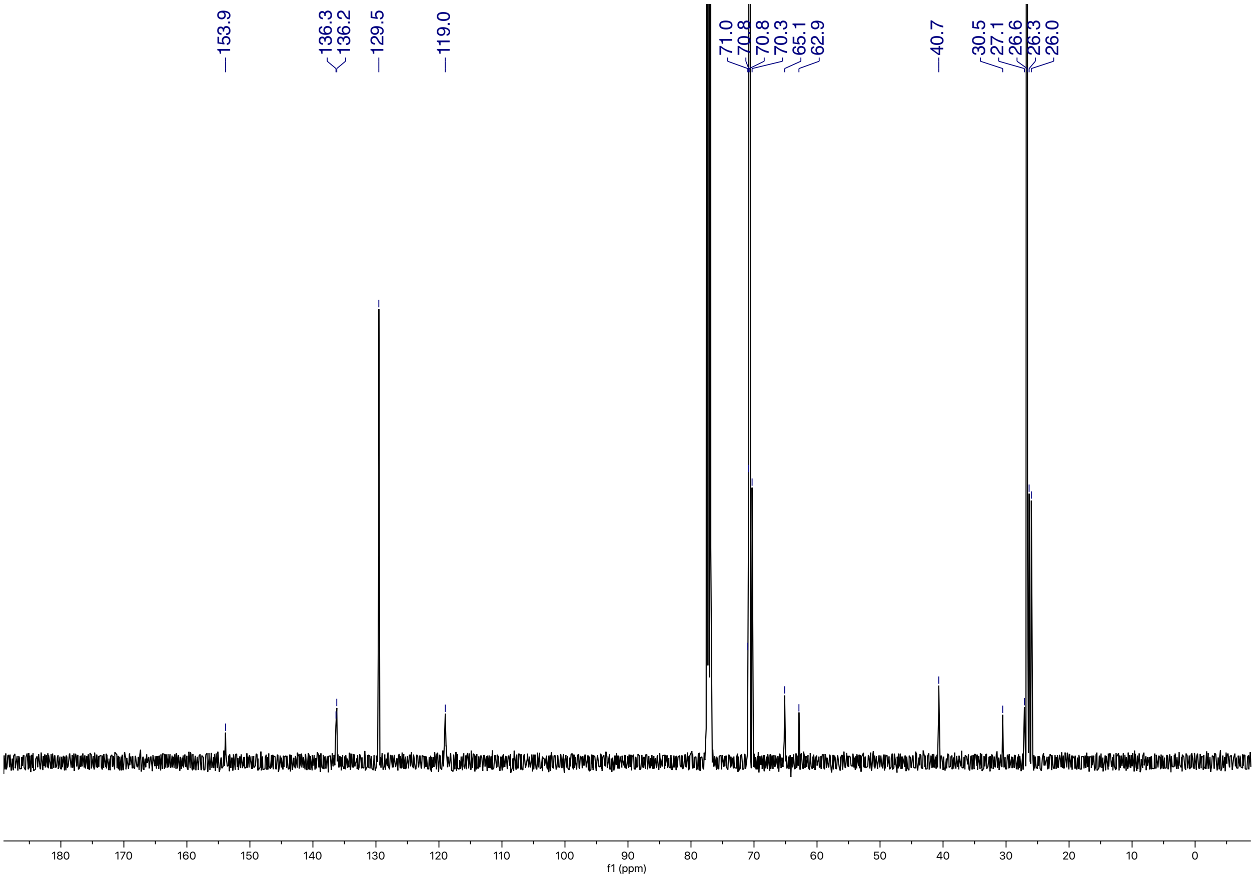

Figure S39. ^{13}C NMR spectrum of PU **4’**.

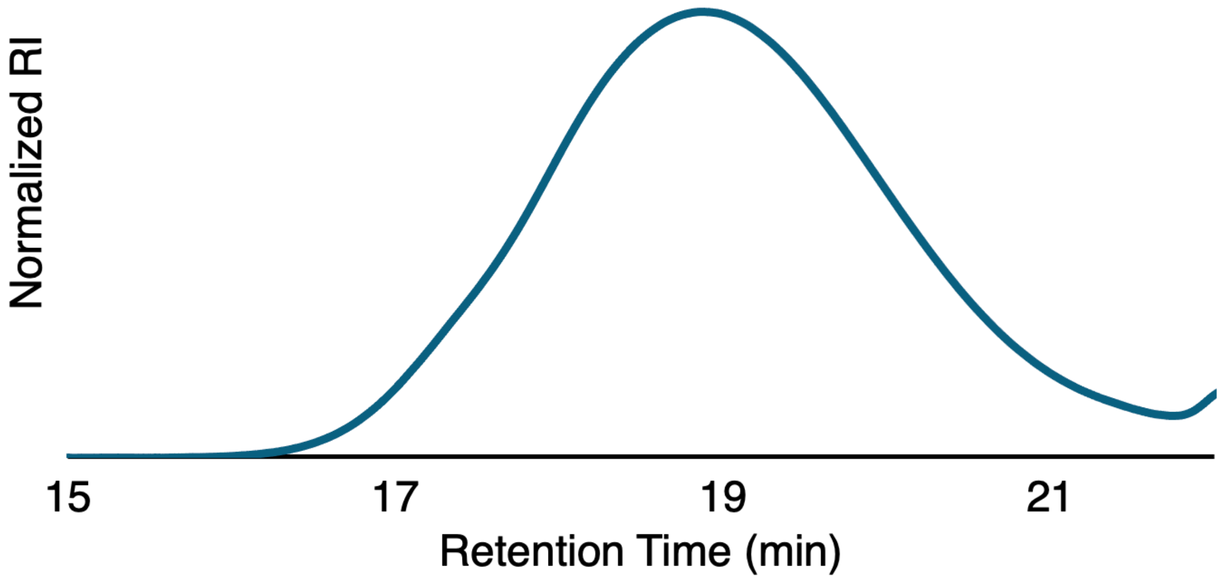

Figure S40. Size exclusion chromatogram of PU **4’**. M_n = 11.3 kDa, $Đ$ = 2.4. Absolute molecular weight was calculated following detector calibration with a single narrow poly(styrene) standard.

Additional NMR Spectra

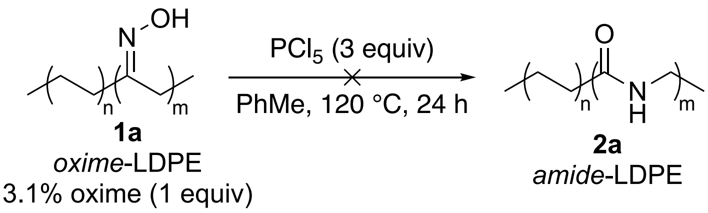

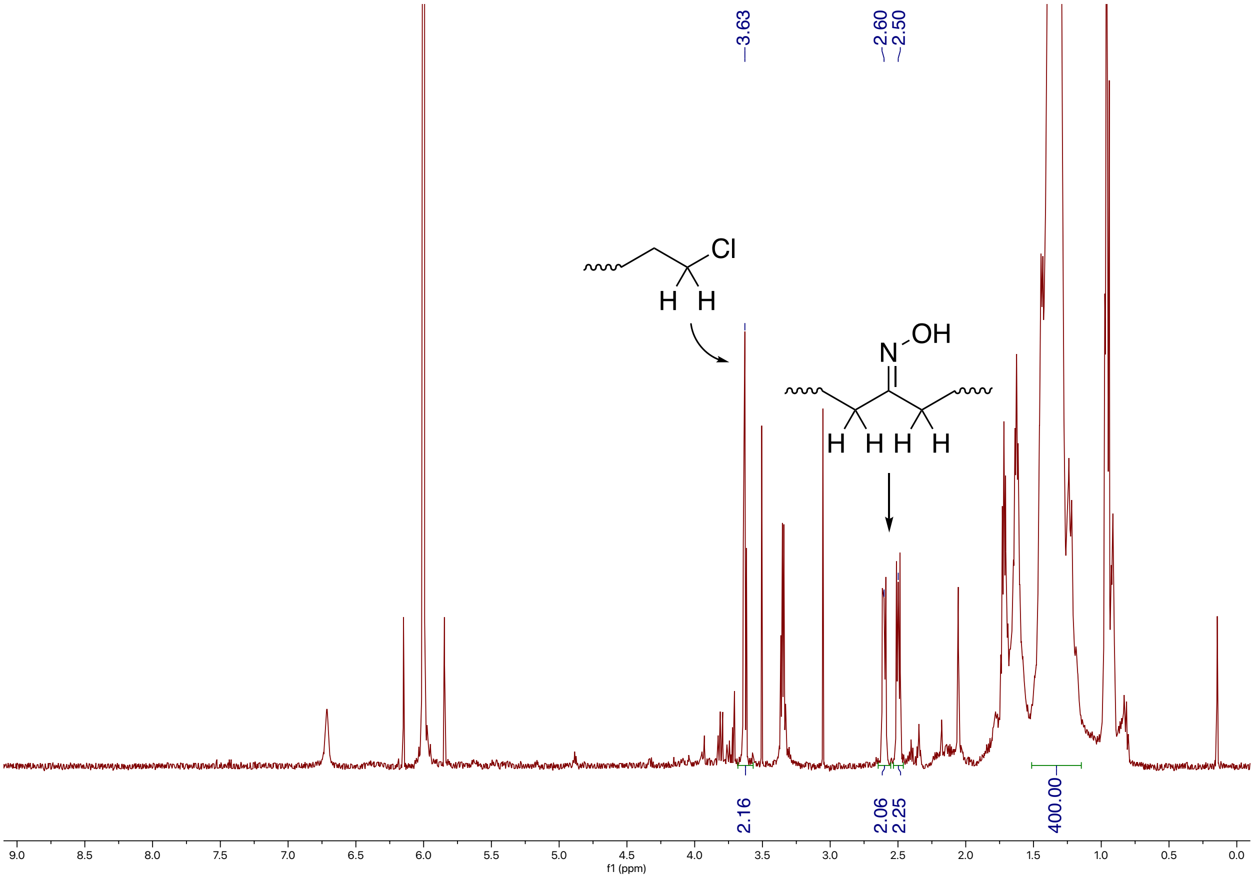

Figure S41. ^{1}H NMR spectrum of the crude product from the reaction of *oxime*-LDPE **1a** and PCl_5.

Materials testing

Procedure for lap shear tests

Lap shear tests were conducted according to ASTM D1002-10 on an Instron universal materials tester equipped with a 5 kN load cell with a shear rate of 1.5 mm/min. Adhesion strength was determined by the maximum load divided by the bonded overlap area, which was measured with digital calipers prior to testing, and the apparent failure mode was assessed visually.

Substrate and Lap Joint Preparation

1. **Degreased Substrates:** To prepare the aluminum, nylon, and glass substrates for adhesive bonding, they were degreased. Substrates were wiped with a fresh Kimwipe soaked in acetone, followed by a second Kimwipe soaked in ethyl acetate. Substrates were air-dried.

   Lap joint preparation: Polymer films of LDPE, polymer **2a**, and PUU **4** (0.1 – 0.3 mm) were prepared on a hot press at 120 °C for 45 seconds to provide melts. Specifically, polymer samples between two Kapton films were pressed between steel plates at 2000 psig. Teflon shims were used to control film thickness. The samples were cooled at room temperature. For testing of aluminum and nylon substrates, a 1 cm x 1 cm piece of the polymer film was cut and the cut films were placed at the end of a clean Al 6061 or nylon-6,6 adherend. For testing of glass substrates, a 2 mm x 2 mm piece of the polymer film was cut and the cut films were placed at the end of a clean glass microscope slide (for glass substrates, rectangular area lap-shear testing consistently led to the failure of the glass due to excessive adhesive force and thus the testing area on glass substrates was reduced to 3–5 mm diameter round-shaped adhesives to produce viable specimens). The substrates were overlapped in an antiparallel arrangement, clamped with two small binder clips, and subsequently transferred to a pre-heated oven. Samples were heated at 140 °C for 3–5 minutes. All samples were allowed to cool slowly to room temperature. Excess polyethylene adhesive was carefully removed from the edges with a razor. Shims were applied to lap joint ends to help align the grip of the mechanical tester. Multiple attempts to prepare lap joints with LDPE failed, as indicated by breaking of the lap joint during the clamping process. Thus, the adhesion strengths of LDPE were unmeasurable by this method. For lap-joints with Gorilla Glue, the adhesive was applied to one substrate at room temperature. Another substrate was aligned in an antiparallel arrangement, clamped with two small binder clips, and allowed to cure for 24 h. All measurable samples were loaded at 1.5 mm/min in shear until failure, and the dimensions of the bonded area were measured with calipers. Finally, the adhesive strength was determined by the peak load divided by the overlap area. Lap shear measurements were repeated for at least three specimens, and the values reported are averages of the measurements of these sets of specimens.

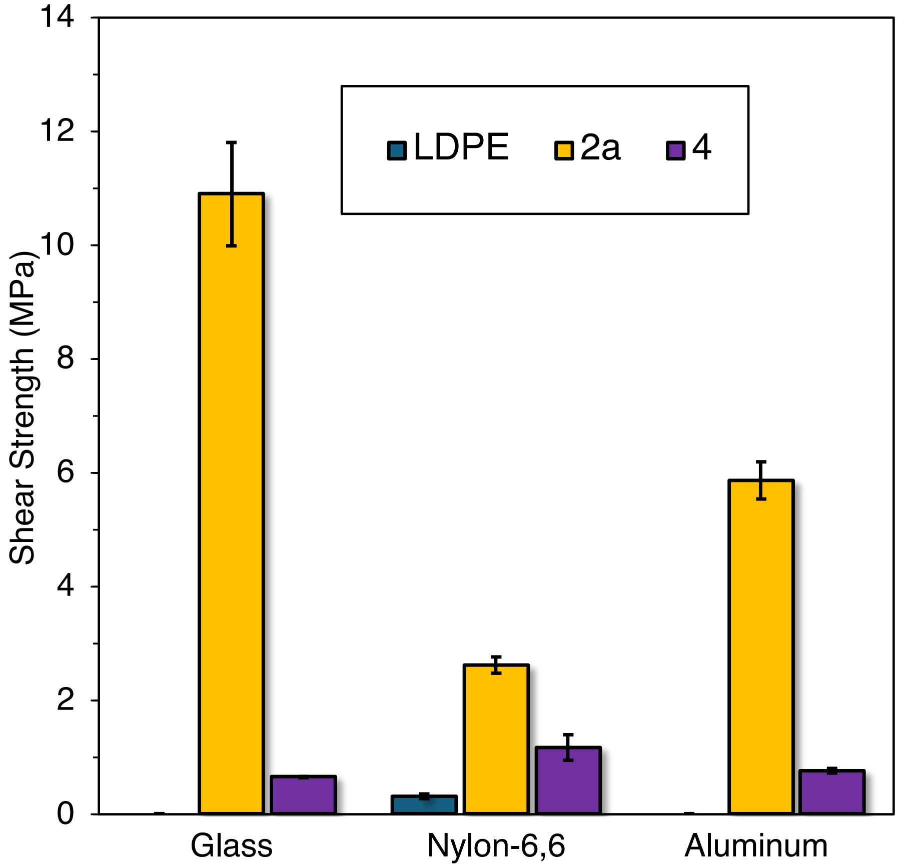

Figure S42. Lap shear strength of joints Al-LDPE-Al, nylon-6,6-LDPE-nylon-6,6, glass-LDPE-glass, Al-**2a**-Al, nylon-6,6-**2a**-nylon-6,6, glass-**2a**-glass, Al-**4**-Al, nylon-6,6-**4**-nylon-6,6, glass-**4**-glass. Error bars represent standard deviations.

Table S2. Summary of results of adhesion strength in lap shear tests

Entry	Interface	Shear Strength (MPa)	Mode of Failure
1[a]	Al-LDPE-Al	--	--
2	nylon-6,6-LDPE-nylon-6,6	0.3 ± 0.04	Adhesive
3[a]	glass-LDPE-glass	--	--
4	Al-**2a**-Al	5.9 ± 0.3	Adhesive
5	nylon-6,6-**2a**-nylon-6,6	2.6 ± 0.1	Adhesive
6	glass-**2a**-glass	10.9 ± 0.9	Adhesive
7	Al-**4**-Al	0.8 ± 0.05	Adhesive
8	nylon-6,6-**4**-nylon-6,6	1.2 ± 0.2	Adhesive
9	glass-**4**-glass	0.7 ± 0.01	Adhesive
10	Al-Gorilla glue-Al	4.3 ± 0.9	--
11	Glass-Gorilla glue-Glass	6.5 ± 1.9	--
12	nylon-6,6–Gorilla glue–nylon-6,6	0.6 ± 0.2	--

[a]Shear strength not measurable because of lap joint failure during the clamping process.

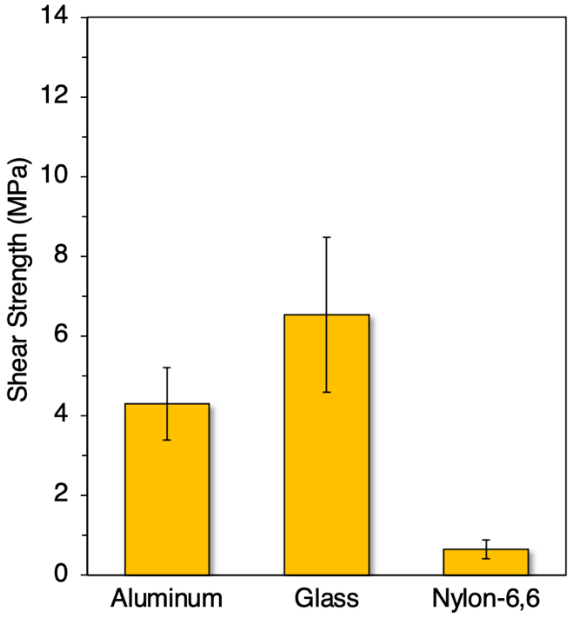

Figure S43. Lap shear strength of joints Al-Gorilla glue-Al, glass-Gorilla glue-glass, and nylon-6,6-Gorilla glue-nylon-6,6. Error bars represent standard deviations.

Procedure for tensile tests

1. **Sample preparation:** Polymer films of LDPE, polymer **2a**, and PUU **4** were prepared on a hot press at 120 °C for 45 seconds to provide melts (350 ± 50µm thickness). Specifically, polymer samples between two Kapton films were pressed between steel plates at 2000 psig. Teflon shims were used to control film thickness. The samples were then cooled at room temperature and cut into a dog-bone geometry using a cutting die (ASTM D-638V) to obtain samples that were 9.53 mm in length and 3.18 mm in width.
2. **Experimental procedures for tensile tests:** Tensile testing was conducted according to ASTM D638 on an Instron universal materials tester. Tensile stress and strain were measured at room temperature using an extension rate of 50 mm/min or 10 mm min^{-1}. Measurements were repeated for at least three samples, and average values are reported.
3. **Experimental procedures for elastic hysteresis tests:** Elastic hysteresis testing was conducted according to ASTM D638 on an Instron universal materials tester. Tensile stress and strain were measured at room temperature using an extension rate of 50 mm/min until the dog-bone was stretched to 60 mm in length and then backwards at a rate of 50 mm/min until the force returned to zero.

Table S3. Summary of results of tensile tests conducted at a strain rate of 50 mm min^{-1}.

Polymer	tensile stress at max load (MPa)	Young's Modulus (MPa)	tensile strain (extension) at break (%)	toughness (MJ/m^3)
LDPE	11.4 ± 1.1	148.5 ± 16.8	227.8 ± 96.8	19.5 ± 9.8
**2a**	8.5 ± 1.6	122.3 ± 14.9	234.0 ± 44.7	18.0 ± 3.8
**4**	5.5 ± 0.9	0.9 ± 0.2	939.2 ± 89.9	29.9 ± 6.6

Table S4. Summary of results of tensile tests conducted at a strain rate of 10 mm min^{-1}.

Polymer	tensile stress at max load (MPa)	Young's Modulus (MPa)	tensile strain (extension) at break (%)	toughness (MJ/m^3)
LDPE	12.6 ± 0.2	186 ± 7	522 ± 64	59 ± 7
**2a**	13.0 ± 0.4	73 ± 4	285 ± 19	31 ± 2
**4**	6.4 ± 0.6	1.0 ± 0.3	900 ± 200	32 ± 8

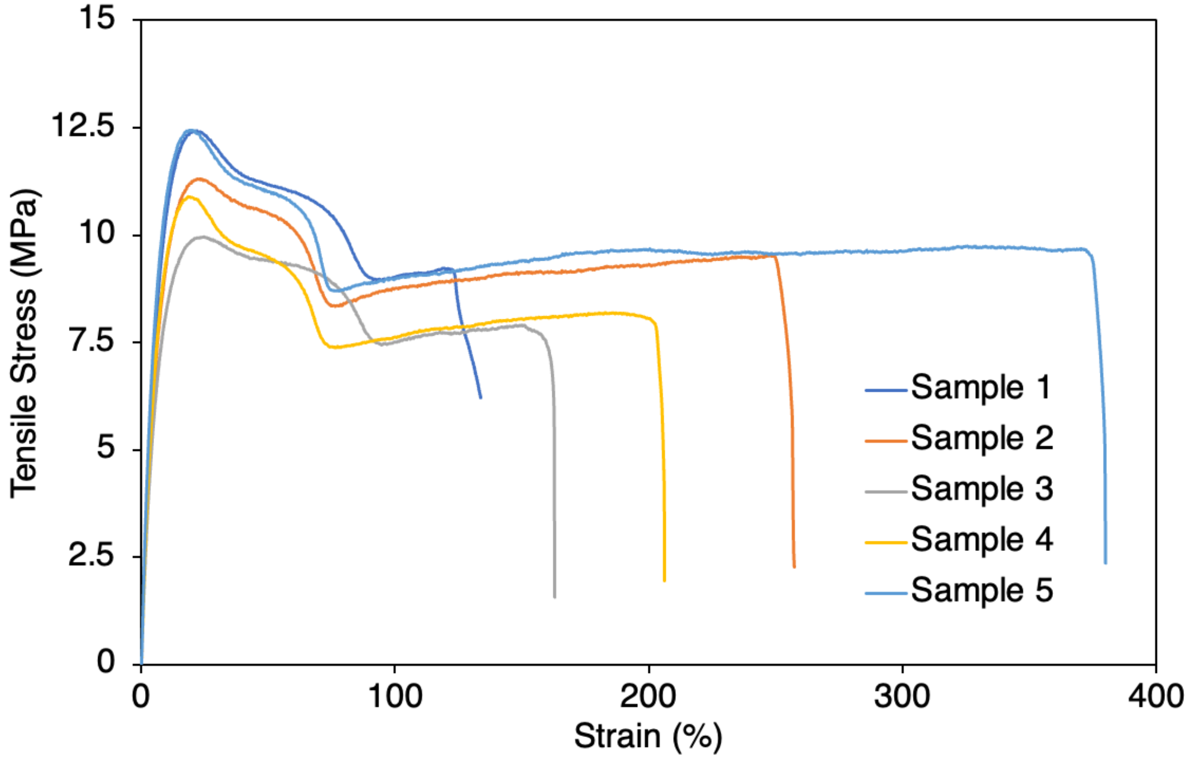

Figure S44. Stress-strain curves for unmodified LDPE conducted at a strain rate of 50 mm min^{-1}.

Table S5. Summary of results of tensile tests for unmodified LDPE conducted at a strain rate of 50 mm min^{-1}.

Sample	tensile stress at max load (MPa)	Young's Modulus (MPa)	tensile strain (extension) at break (%)	toughness (MJ/m^3)
**1**	12.4	160.4	133.7	13.4
**2**	11.3	142.4	256.7	13.5
**3**	10.0	125.8	162.8	17.1
**4**	10.9	144.9	206.1	36.7
**5**	12.5	169.0	379.8	16.9

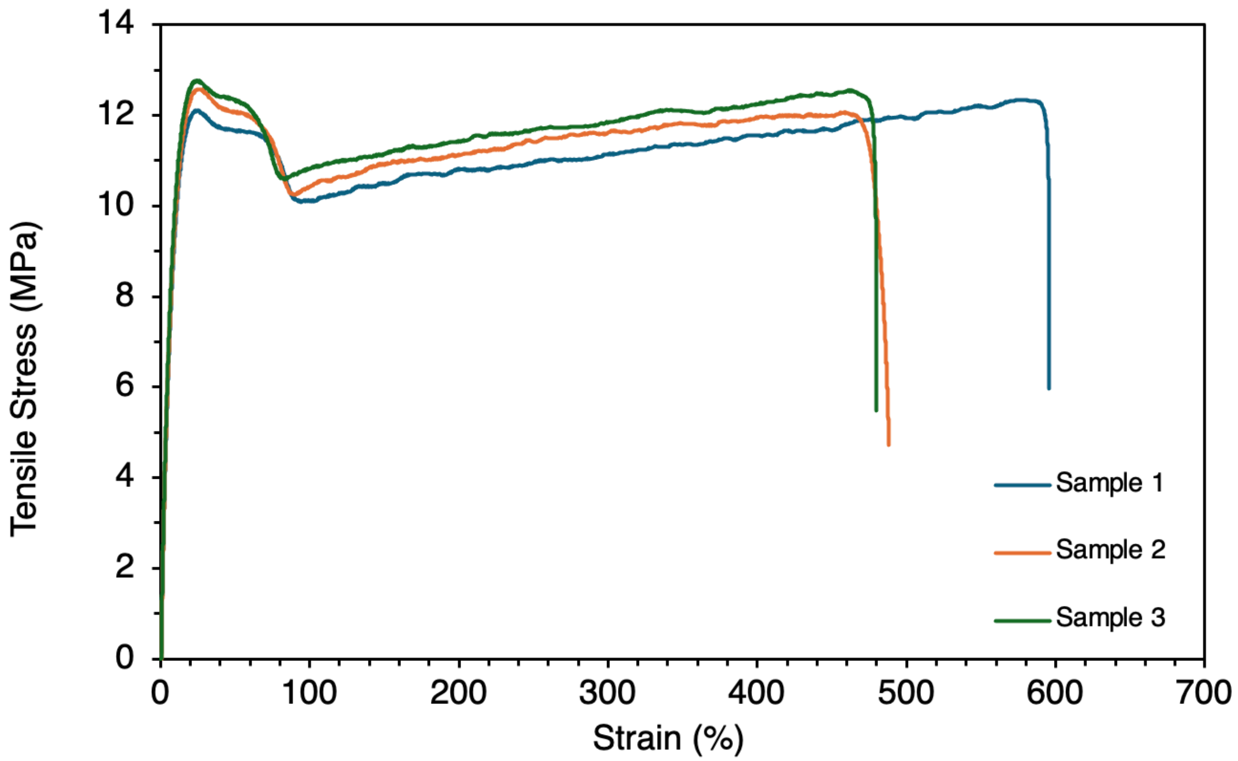

Figure S45. Stress-strain curves for unmodified LDPE conducted at a strain rate of 10 mm min^{-1}.

Table S6. Summary of results of tensile tests for unmodified LDPE conducted at a strain rate of 10 mm min^{-1}.

Sample	tensile stress at max load (MPa)	Young's Modulus (MPa)	tensile strain (extension) at break (%)	toughness (MJ/m^3)
**1**	12.4	178.3	595.5	66.9
**2**	12.6	186.6	490.9	55.2
**3**	12.8	191.8	479.6	56.0

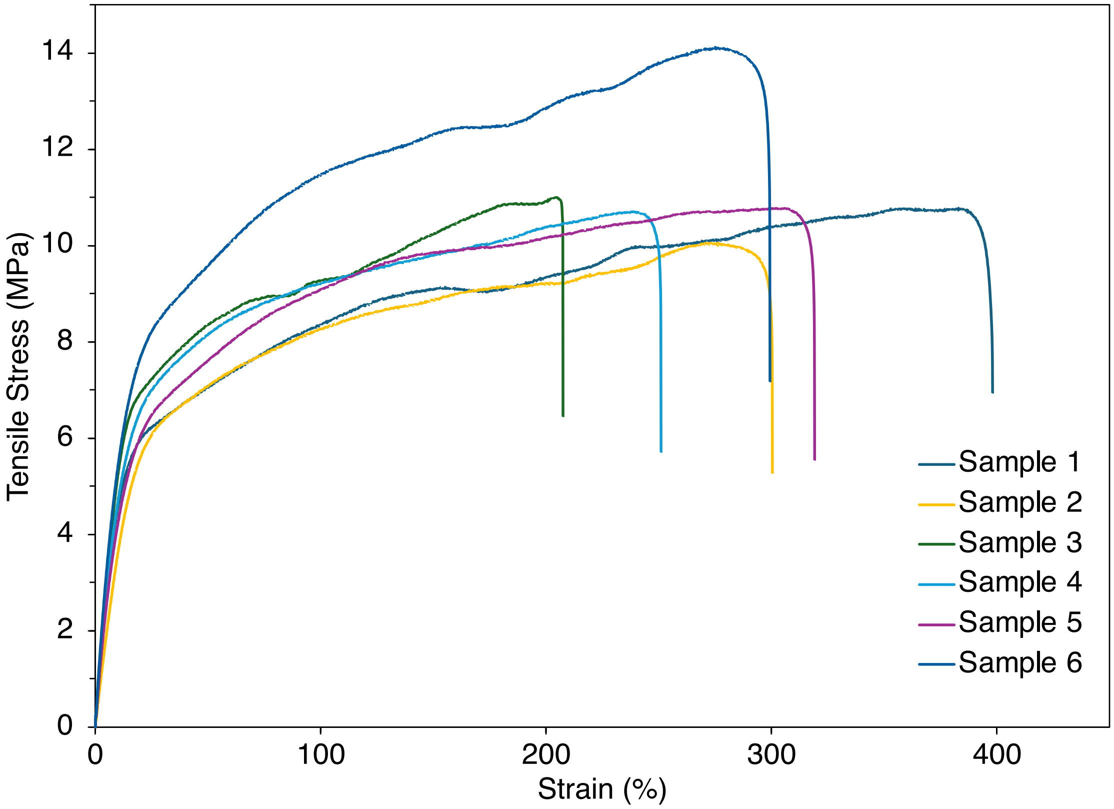

Figure S46. Stress-strain curves for polymer **2a** conducted at a strain rate of 50 mm min^{-1}.

Table S7. Summary of results of tensile tests for polymer **2a** conducted at a strain rate of 50 mm min^{-1}.

Sample	tensile stress at max load (MPa)	Young's Modulus (MPa)	tensile strain (extension) at break (%)	toughness (MJ/m^3)
**1**	10.8	65.9	429.0	35.1
**2**	10.1	39.1	317.8	24.8
**3**	11.0	57.3	207.4	16.7
**4**	10.7	60.1	250.9	22.6
**5**	10.6	52.8	319.1	29.4
**6**	13.9	66.9	299.2	34.6

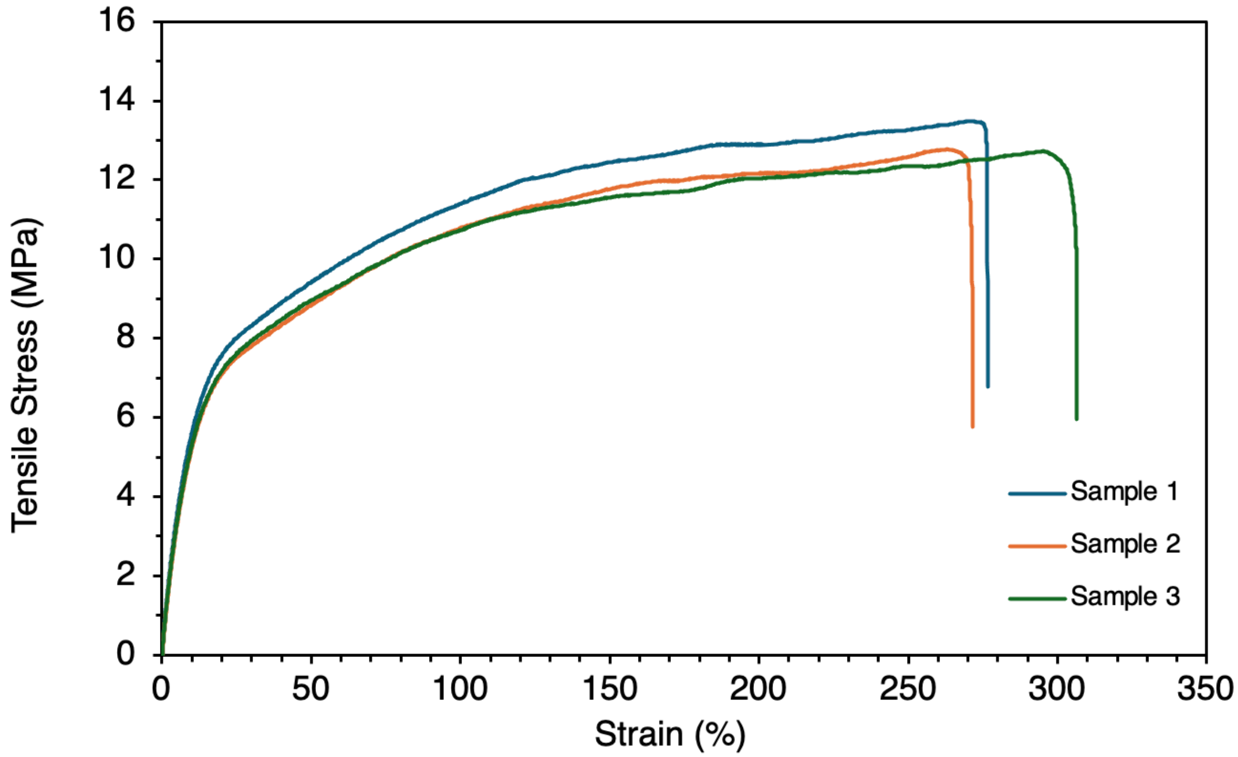

Figure S47. Stress-strain curves for polymer **2a** conducted at a strain rate of 10 mm min^{-1}.

Table S8. Summary of results of tensile tests for polymer **2a** conducted at a strain rate of 10 mm min^{-1}.

Sample	tensile stress at max load (MPa)	Young's Modulus (MPa)	tensile strain (extension) at break (%)	toughness (MJ/m^3)
**1**	13.5	77.3	276.5	31.3
**2**	12.8	69.6	271.5	28.8
**3**	12.7	71.4	306.5	33.0

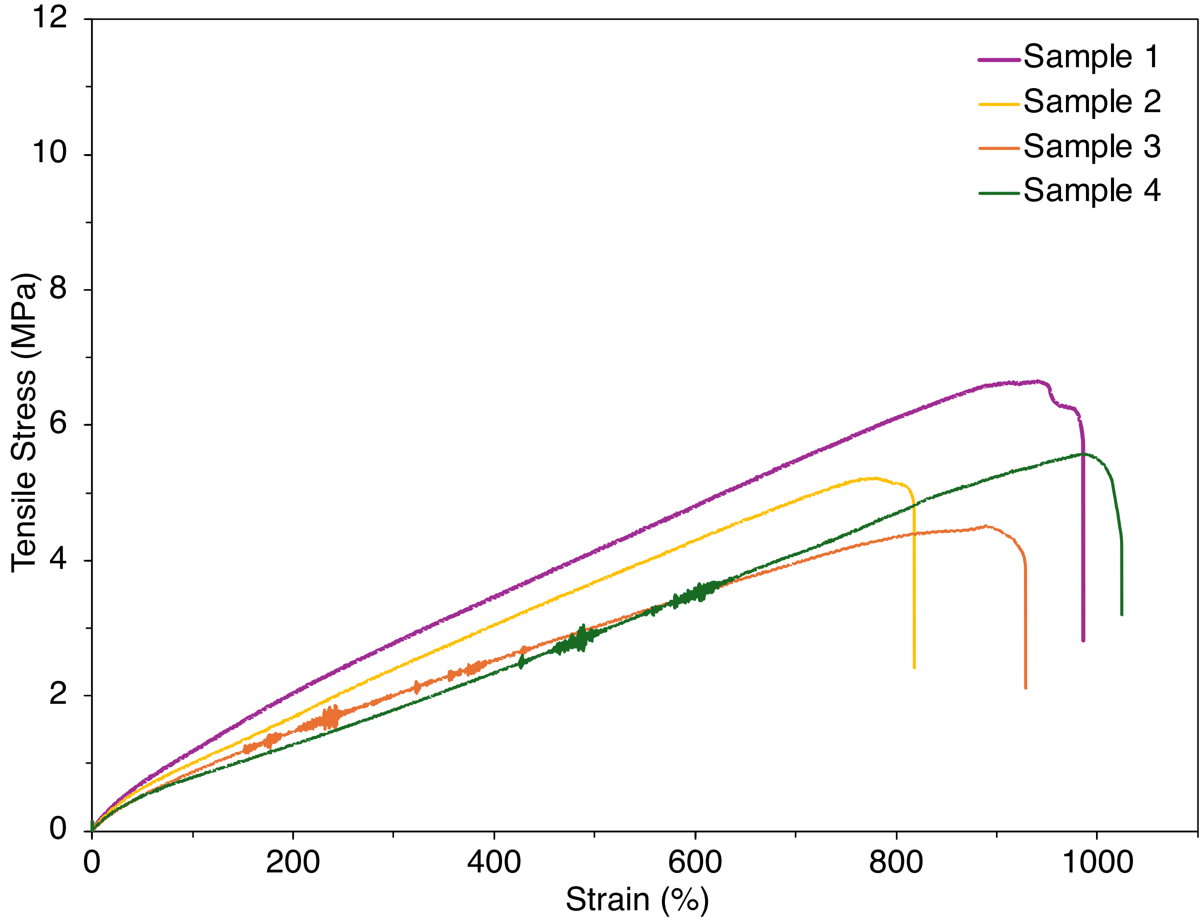

Figure S48. Stress-strain curves for polymer **4** conducted at a strain rate of 50 mm min^{-1}.

Table S9. Summary of results of tensile tests for polymer **4** conducted at a strain rate of 50 mm min^{-1}.

Sample	tensile stress at max load (MPa)	Young's Modulus (MPa)	tensile strain (extension) at break (%)	toughness (MJ/m^3)
**1**	6.7	1.1	986.0	39.0
**2**	5.2	1.0	818.0	24.6
**3**	4.5	0.8	928.7	25.3
**4**	5.6	0.6	1024.3	30.8

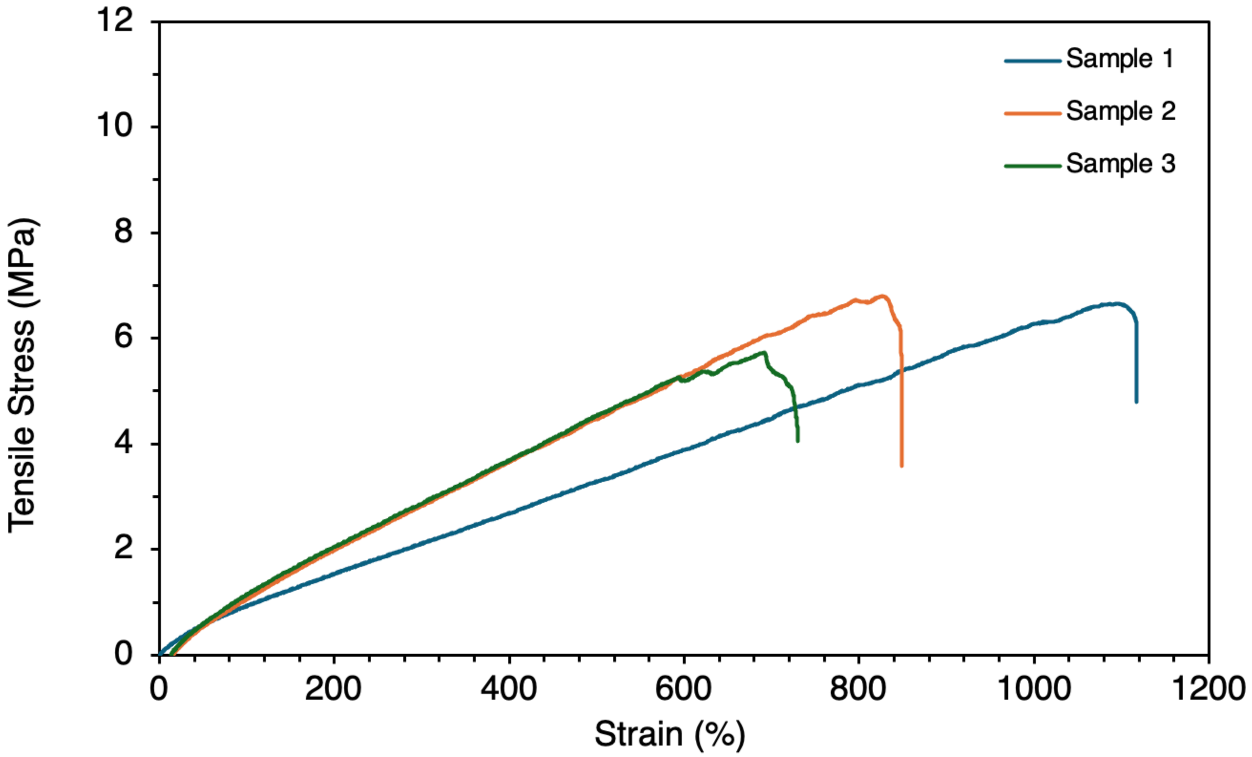

Figure S49. Stress-strain curves for polymer **4** conducted at a strain rate of 10 mm min^{-1}.

Table S10. Summary of results of tensile tests for polymer **4** conducted at a strain rate of 10 mm min^{-1}.

Sample	tensile stress at max load (MPa)	Young's Modulus (MPa)	tensile strain (extension) at break (%)	toughness (MJ/m^3)
**1**	6.7	0.8	1120.6	40.5
**2**	6.8	1.1	850.2	32.0
**3**	5.7	1.3	732.3	23.9

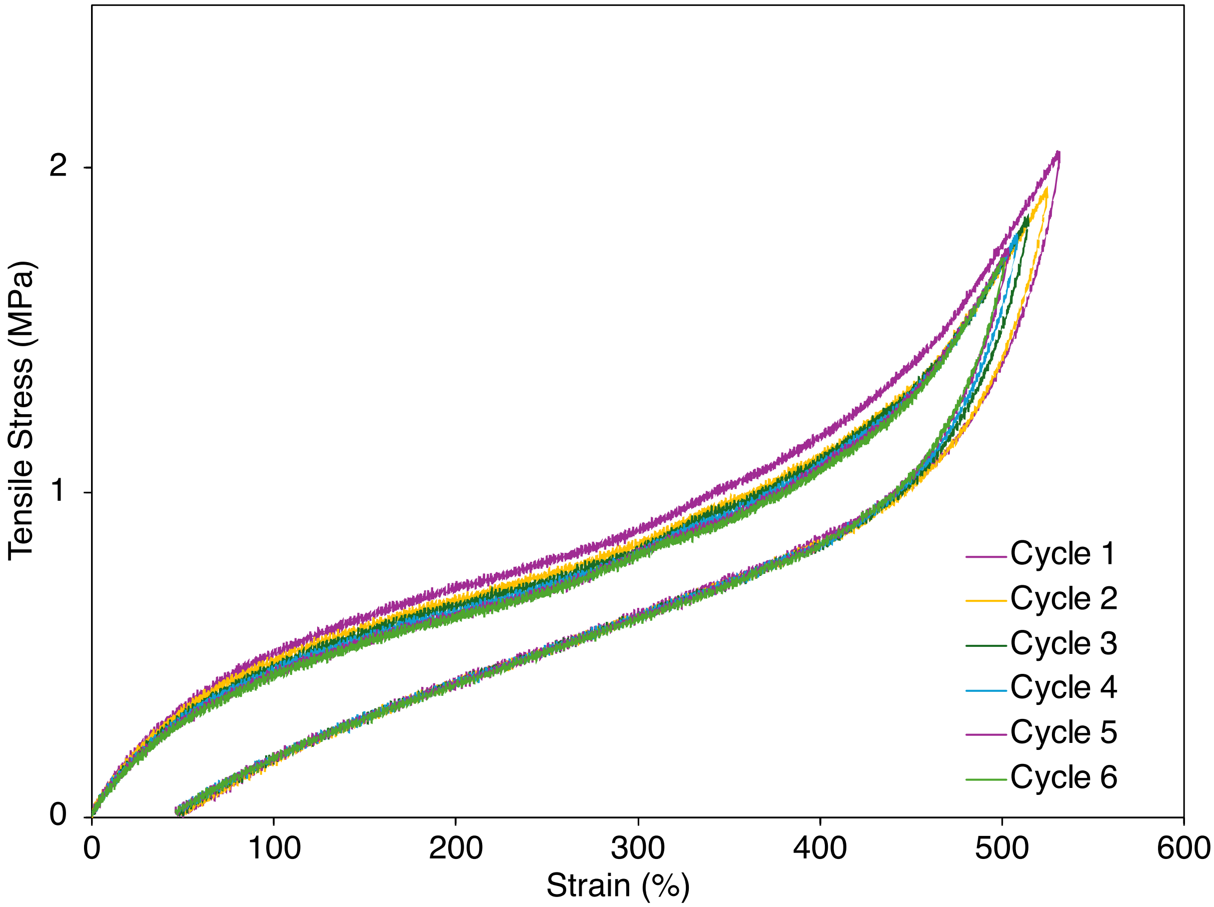

Figure S50. Elastic hysteresis curves of polymer **4** conducted at a strain rate of 50 mm min^{-1}.

Table S11. Summary of results of elastic hysteresis experiments

Entry	Cycle	SR[a] (%)
1	Cycle 1	91.2
2	Cycle 2	90.8
3	Cycle 3	91.1
4	Cycle 4	90.8
5	Cycle 5	90.9
6	Cycle 6	90.9

[a]Strain recovery, SR, determined using the equation $100(\varepsilon_a - \varepsilon_r)/\varepsilon_a$, where ε_a = applied strain and ε_r = strain at zero load.[11]

Additional DSC Experiments

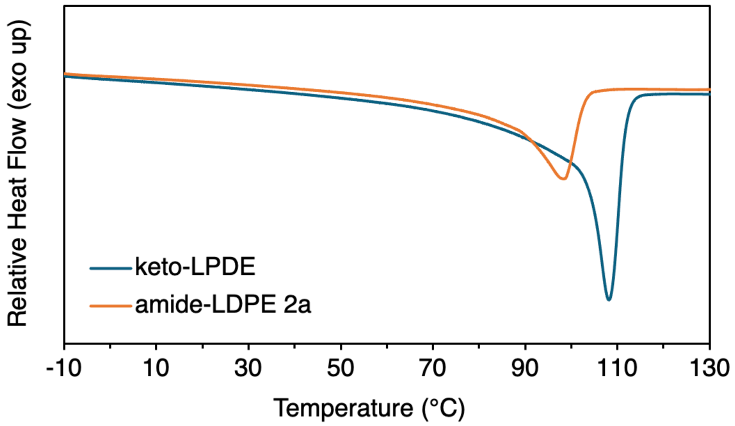

Figure S51. DSC of *keto*-LDPE (blue, T_m = 108.3 °C) and *amide*-LDPE **2a** (orange, T_m = 98.7 °C).

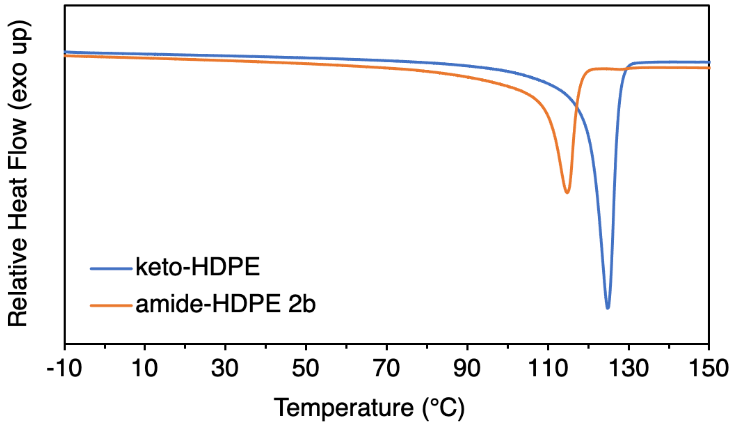

Figure S52. DSC of *keto*-HDPE (blue, T_m = 124.8 °C) and *amide*-HDPE **2b** (orange, T_m = 114.8 °C).

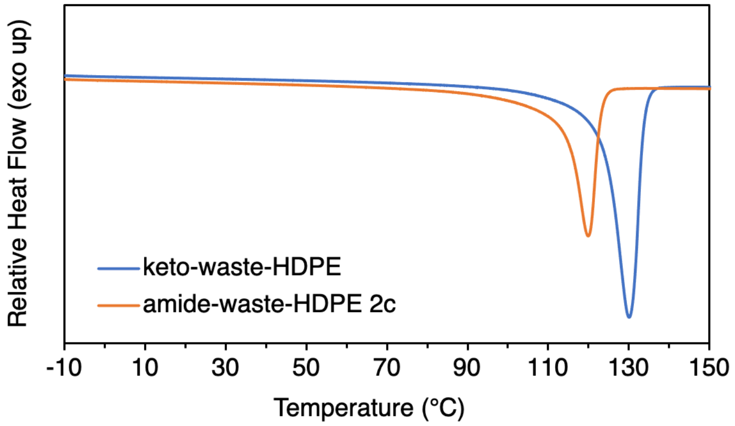

Figure S53. DSC of *keto*-*waste*-HDPE (blue, T_m = 130.1 °C) and *amide*-*waste*-HDPE **2c** (orange, T_m = 119.9 °C).

Table S12. Summary of results of DSC experiments

Entry	Sample	T_m (°C)
1	*keto*-LDPE	108.3
2	*amide*-LDPE **2a**	98.7
3	*keto*-HDPE	124.8
4	*amide*-HDPE **2b**	114.8
5	*keto-waste*-HDPE	130.1
6	*amide-waste*-HDPE **2c**	119.9

References

1 L. Chen, K. G. Malollari, A. Uliana, D. Sanchez, P. B. Messersmith and J. F. Hartwig, *Chem*, 2021, **7**, 137.

2 J. X. Shi, N. R. Ciccia, S. Pal, D. D. Kim, J. N. Brunn, C. Lizandara-Pueyo, M. Ernst, A. M. Haydl, P. B. Messersmith, B. A. Helms and J. F. Hartwig, *J. Am. Chem. Soc.*, 2023, **145**, 21527.

3 D. Lorenzo, A. Romero, L. Del-Arco and A. Santos, *Industrial & Engineering Chemistry Research*, 2019, **58**, 11878.

4 J. Moran, J. Y. Pfeiffer, S. I. Gorelsky and A. M. Beauchemin, *Org. Lett.*, 2009, **11**, 1895.

5 W. Zhou, P. Neumann, M. Al Batal, F. Rominger, A. S. K. Hashmi and T. Schaub, *ChemSusChem*, 2021, **14**, 4176.

6 E. Balaraman, B. Gnanaprakasam, L. J. W. Shimon and D. Milstein, *J. Am. Chem. Soc.*, 2010, **132**, 16756.

7 J. Zhang, G. Leitus, Y. Ben-David and D. Milstein, *J. Am. Chem. Soc.*, 2005, **127**, 10840.

8 T. Miura, I. E. Held, S. Oishi, M. Naruto and S. Saito, *Tetrahedron Lett.*, 2013, **54**, 2674.

9 T. Miura, M. Naruto, K. Toda, T. Shimomura and S. Saito, *Sci. Rep.*, 2017, **7**, 1586.

10 I. D. Tomlinson, J. C. May, R. A. Harris, K. M. Buck, S. J. Rosenthal, J. A. McLean and D. M. Hercules, *Polymer*, 2022, **254**, 125069.

11 K. S. O'Connor, A. Watts, T. Vaidya, A. M. LaPointe, M. A. Hillmyer and G. W. Coates, *Macromol.*, 2016, **49**, 6743.
